# Supplementary material for: LGG-1/GABARAP lipidation is not required for autophagy and development in Caenorhabditis elegans
Source: eLife. 2023 Jul 3;12:e85748. doi: 10.7554/eLife.85748 (PMC10338037; doi:10.7554/eLife.85748)
Supplement: Figure 1—source data 1. [file elife-85748-fig1-data1.zip › Figure1-Source_Data1/D-L/figure1-KL-Comptage LGG-1.pdf]

LGG-1 puncta early embryo

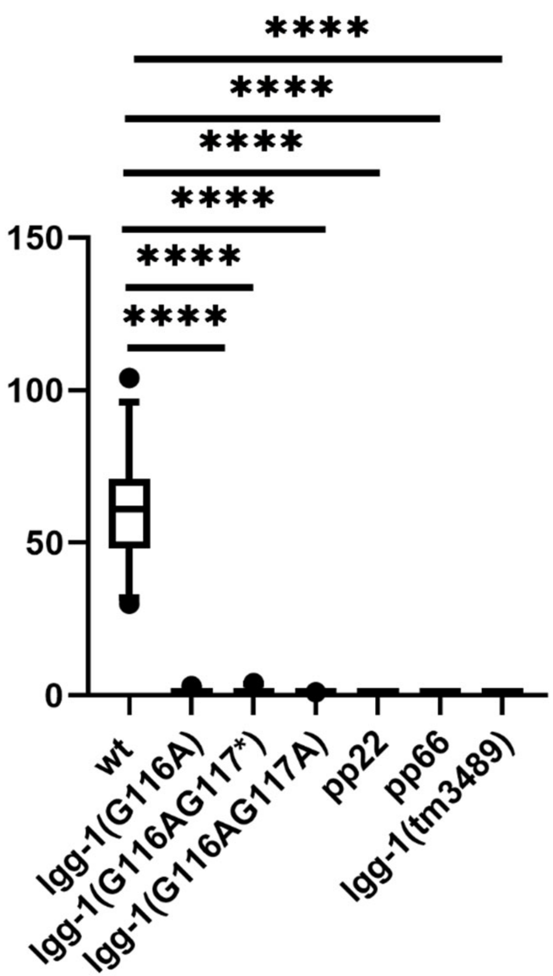

LGG-1 puncta late embryo

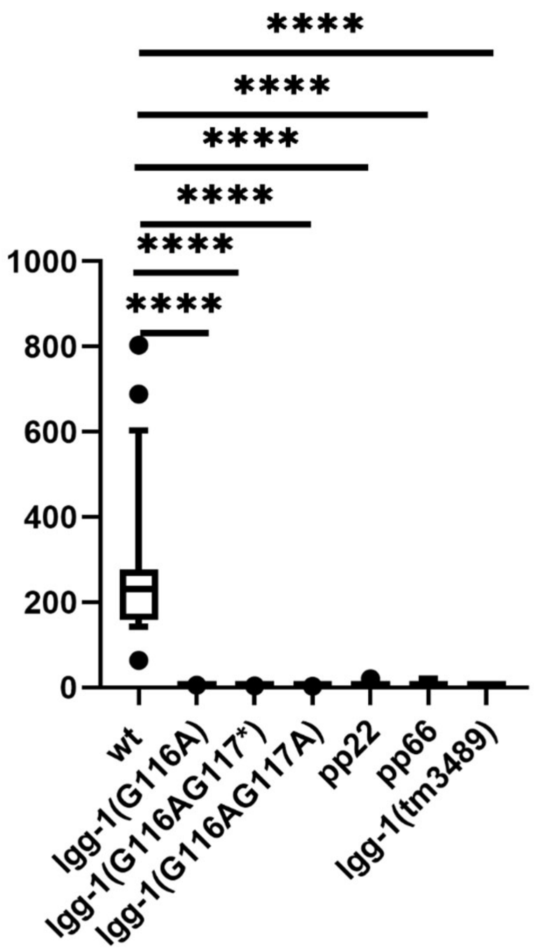

Test man Whitney p value 0,05

Table Analyzed	manip de 2018,12,20
Column B	atg4,1
vs.	vs,
Column A	N2
Unpaired t test with Welch's correction	
P value	0,0081
P value summary	**
Significantly different (P < 0.05)?	Yes
One- or two-tailed P value?	Two-tailed
Welch-corrected t, df	t=3,417, df=8,696

Table Analyzed	manip de 2018,12,20
Column C	lgg-1(G116A)
vs.	vs,
Column B	atg4,1
Unpaired t test with Welch's correction	
P value	0,0002
P value summary	***
Significantly different (P < 0.05)?	Yes
One- or two-tailed P value?	Two-tailed
Welch-corrected t, df	t=9,544, df=5,000

Table Analyzed	manip de 2018,12,20
Column C	lgg-1(G116A)
vs.	vs,
Column A	N2
Unpaired t test with Welch's correction	
P value	<0,0001
P value summary	****
Significantly different (P < 0.05)?	Yes
One- or two-tailed P value?	Two-tailed
Welch-corrected t, df	t=20,54, df=5,000

Complément Figure 1- Graph number of LGG-1 puncta in embryos

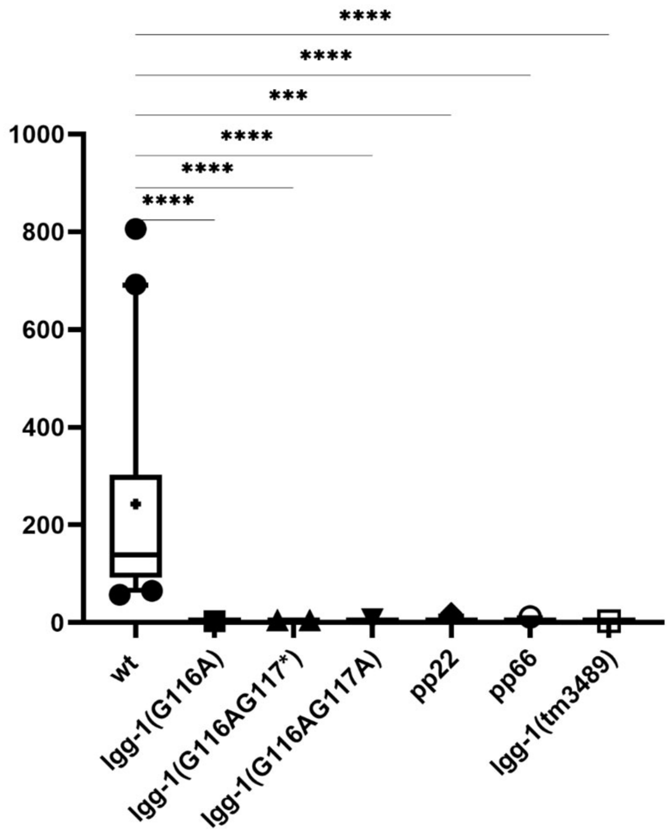

10-90 percentile
+ is mean

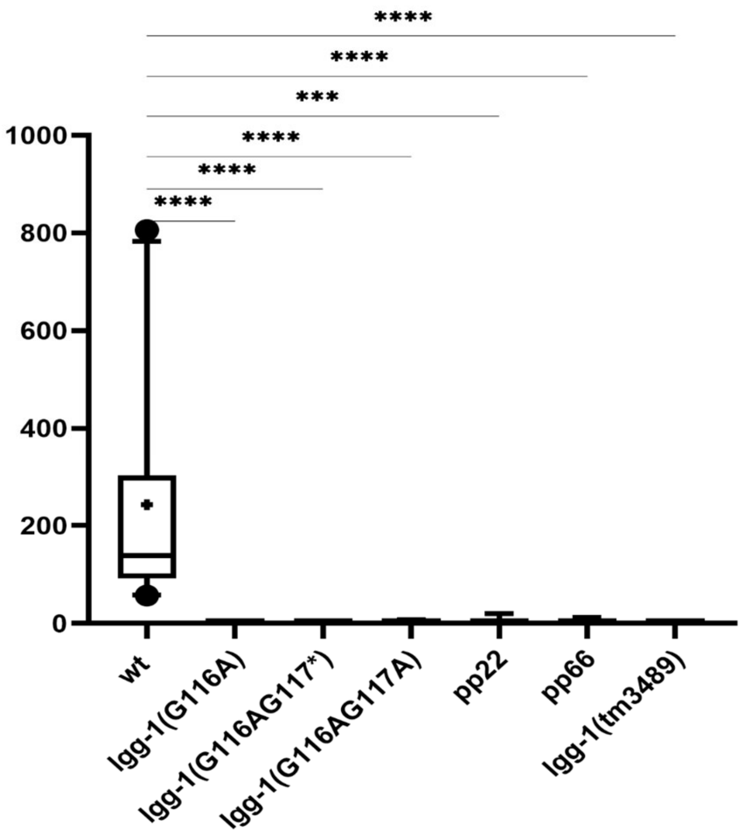

5-95 percentile
+ is mean

Kruskall Wallis test

Dunn's multiple comparisons test	Mean rank diff,	Significant?	Summary	Adjusted P Value	
wt vs. lgg-1(G116A)	64,47	Yes	****	<0,0001	A-B
wt vs. lgg-1(G116AG117*)	44,86	Yes	****	<0,0001	A-C
wt vs. lgg-1(G116AG117A)	63,93	Yes	****	<0,0001	A-D
wt vs. pp22	43,93	Yes	***	0,0010	A-E
wt vs. pp66	59,50	Yes	****	<0,0001	A-F
wt vs. lgg-1(tm3489)	71,71	Yes	****	<0,0001	A-G
lgg-1(G116A) vs. lgg-1(G116AG117*)	-19,62	No	ns	>0,9999	B-C
lgg-1(G116A) vs. lgg-1(G116AG117A)	-0,5437	No	ns	>0,9999	B-D
lgg-1(G116A) vs. pp22	-20,54	No	ns	>0,9999	B-E
lgg-1(G116A) vs. pp66	-4,972	No	ns	>0,9999	B-F
lgg-1(G116A) vs. lgg-1(tm3489)	7,236	No	ns	>0,9999	B-G
lgg-1(G116AG117*) vs. lgg-1(G116AG117A)	19,07	No	ns	>0,9999	C-D
lgg-1(G116AG117*) vs. pp22	-0,9286	No	ns	>0,9999	C-E
lgg-1(G116AG117*) vs. pp66	14,64	No	ns	>0,9999	C-F
lgg-1(G116AG117*) vs. lgg-1(tm3489)	26,85	No	ns	0,4152	C-G
lgg-1(G116AG117A) vs. pp22	-20,00	No	ns	>0,9999	D-E
lgg-1(G116AG117A) vs. pp66	-4,429	No	ns	>0,9999	D-F
lgg-1(G116AG117A) vs. lgg-1(tm3489)	7,780	No	ns	>0,9999	D-G
pp22 vs. pp66	15,57	No	ns	>0,9999	E-F
pp22 vs. lgg-1(tm3489)	27,78	No	ns	0,5576	E-G
pp66 vs. lgg-1(tm3489)	12,21	No	ns	>0,9999	F-G
