# Supplementary material for: LGG-1/GABARAP lipidation is not required for autophagy and development in Caenorhabditis elegans
Source: eLife. 2023 Jul 3;12:e85748. doi: 10.7554/eLife.85748 (PMC10338037; doi:10.7554/eLife.85748)
Supplement: Figure 1—source data 1. [file elife-85748-fig1-data1.zip › Figure1-Source_Data1/M/Source data Fig 1 panel M.pdf]

Source data for Figure 1 M

1. N2

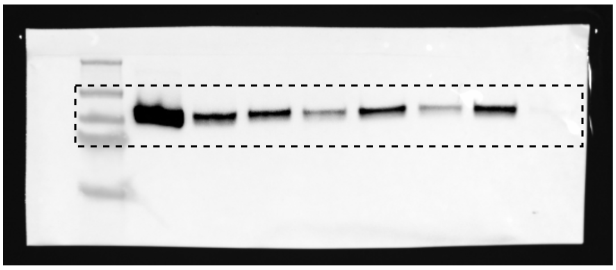
anti-CDC-48

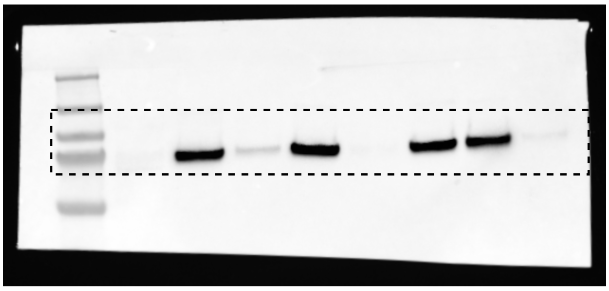
anti-SEL-1

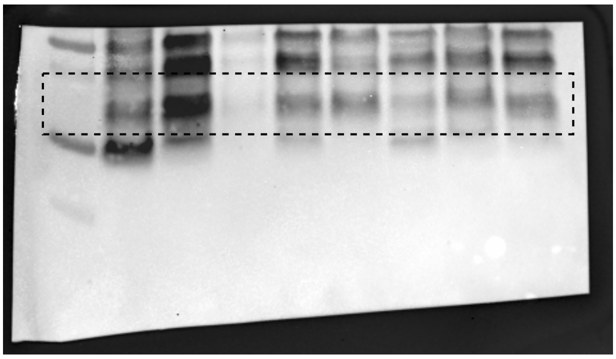
anti-LGG-2

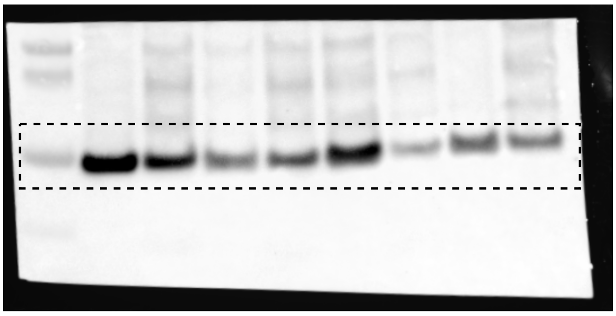
anti-LGG-1

2. lgg-1(G116A)

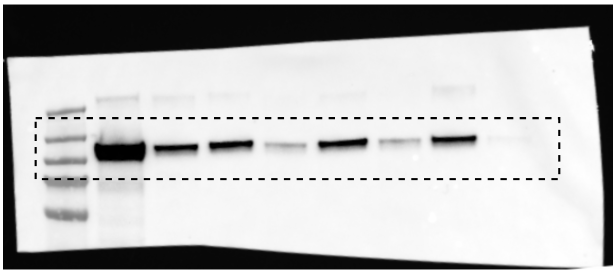
anti-CDC-48

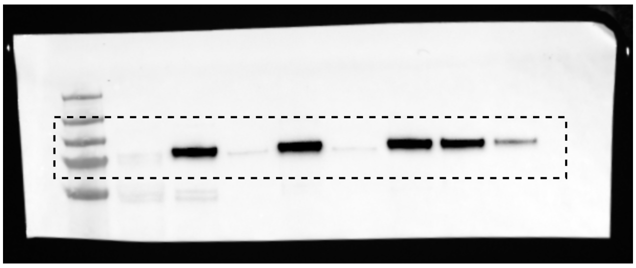
anti-SEL-1

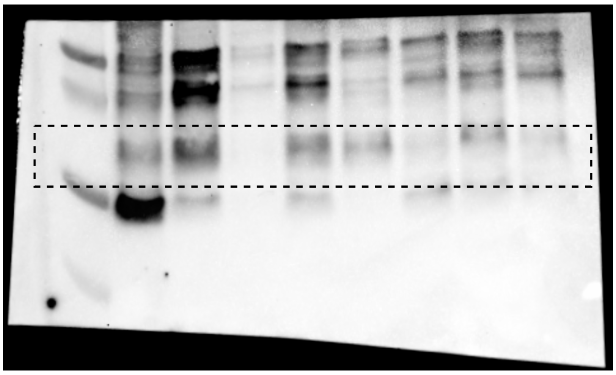
anti-LGG-2

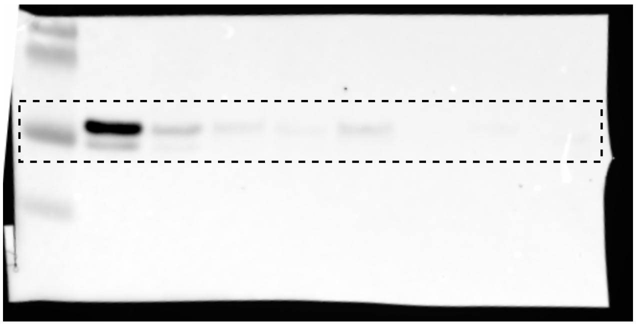
anti-LGG-1

3. lgg-1(G116G117*)

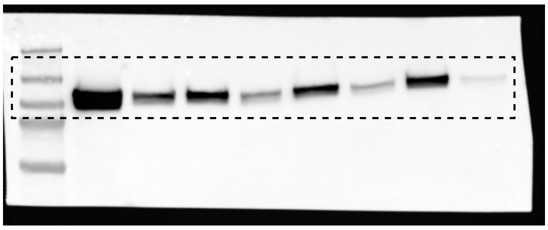

anti-CDC-48

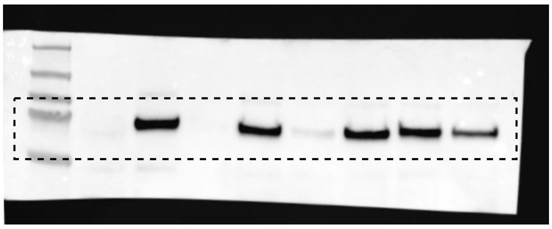

anti-SEL-1

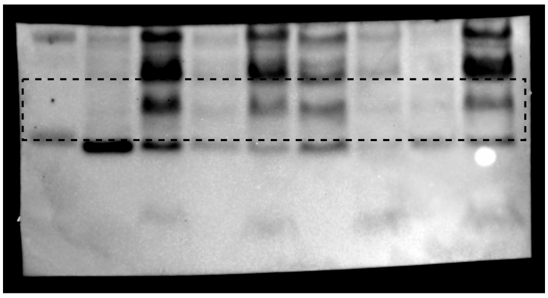

anti-LGG-2

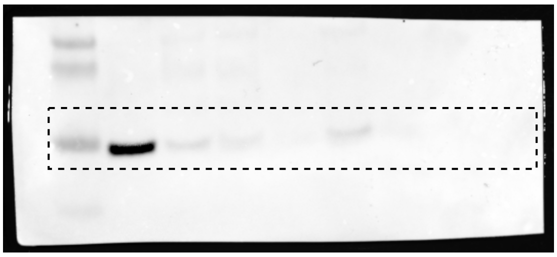

anti-LGG-1
