# Supplementary figures and images for: LGG-1/GABARAP lipidation is not required for autophagy and development in Caenorhabditis elegans (part 1 of 2)
Source: eLife. 2023 Jul 3;12:e85748. doi: 10.7554/eLife.85748 (PMC10338037; doi:10.7554/eLife.85748)

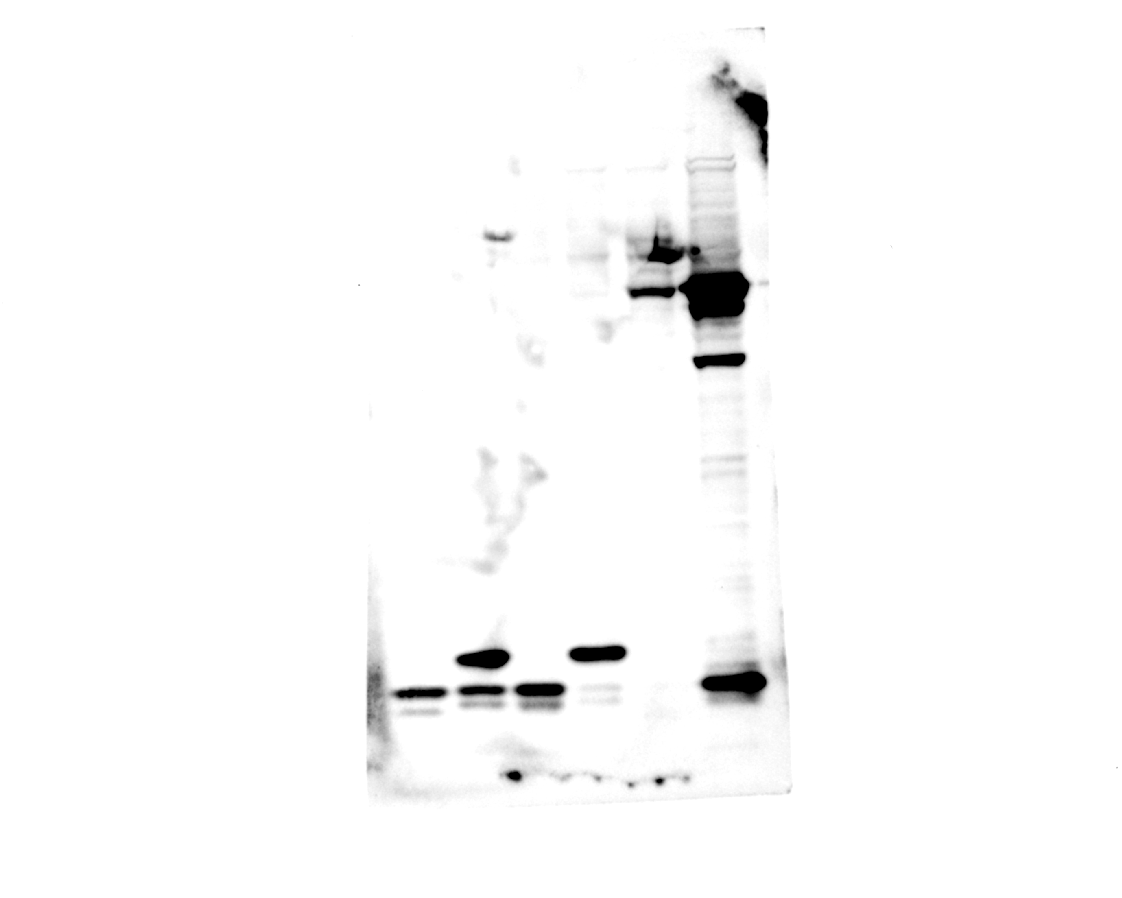

Supplement: Figure 1—source data 1. [file elife-85748-fig1-data1.zip › Figure1-Source_Data1/C/2users 2019-11-08 15h38m42s(Chemiluminescence).tif]

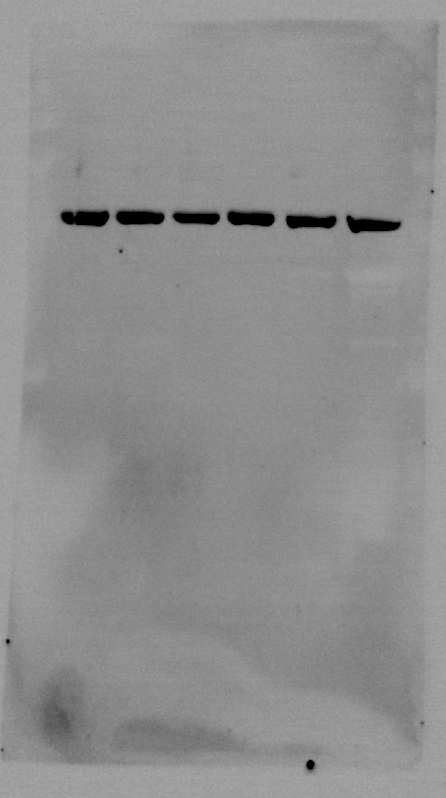

Supplement: Figure 1—source data 1. [file elife-85748-fig1-data1.zip › Figure1-Source_Data1/C/Tubulin2019-11-12 17h28m27s.jpg]

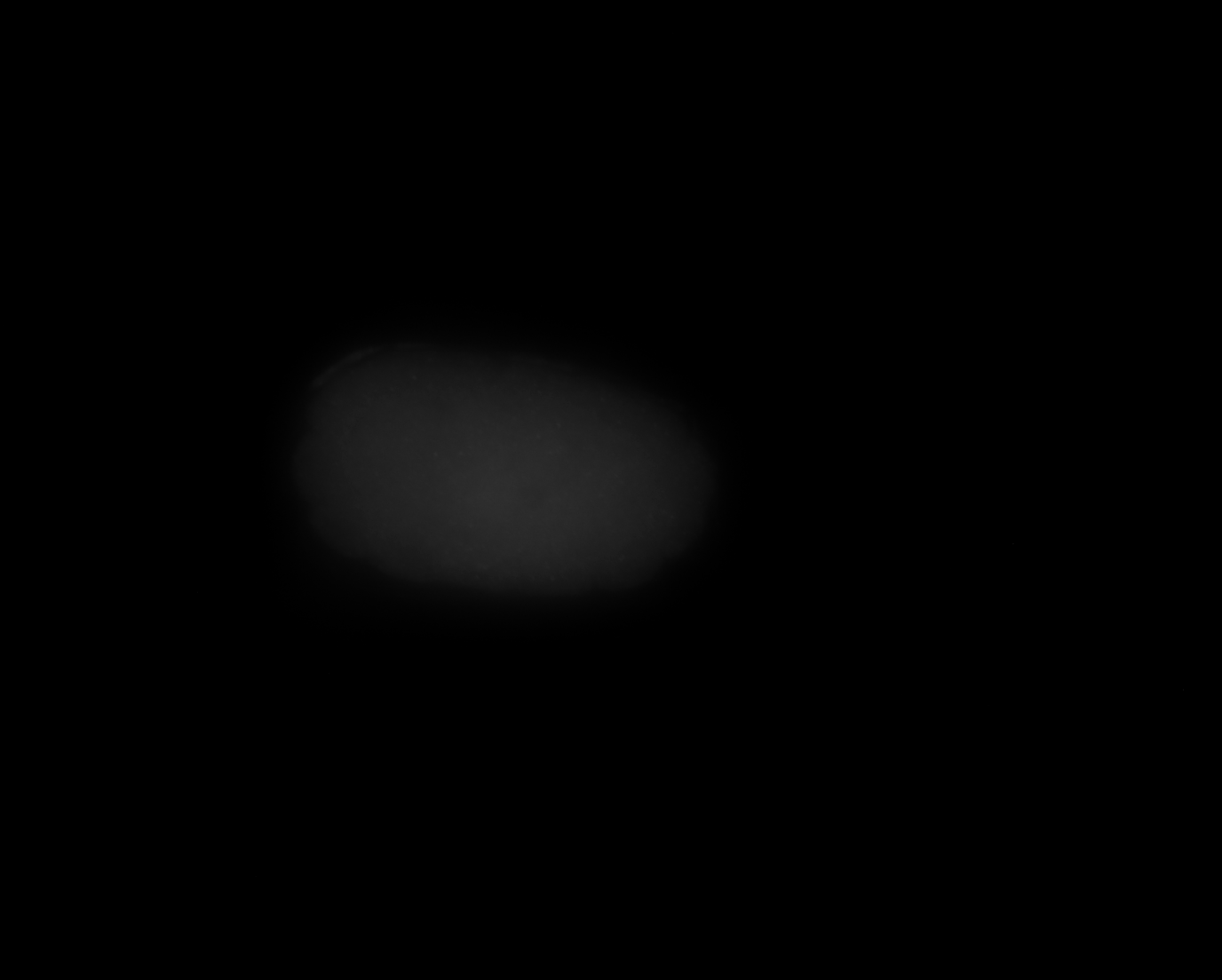

Supplement: Figure 1—source data 1. [file elife-85748-fig1-data1.zip › Figure1-Source_Data1/D-L/lgg-1(tm3489)-100C-AbLGG-1.tif]

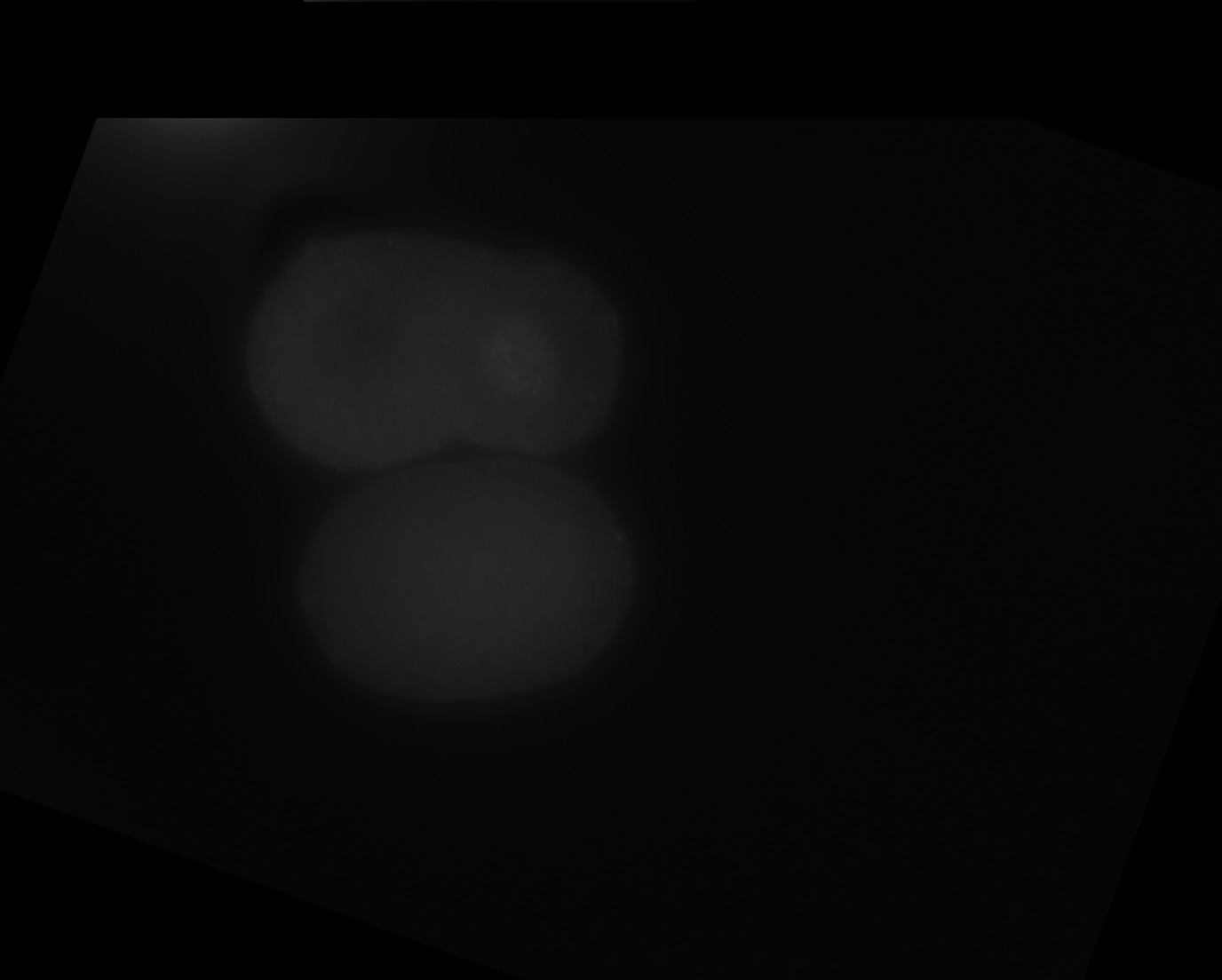

Supplement: Figure 1—source data 1. [file elife-85748-fig1-data1.zip › Figure1-Source_Data1/D-L/lgg-1(tm3489)-précoce-AbLGG-1.tif]

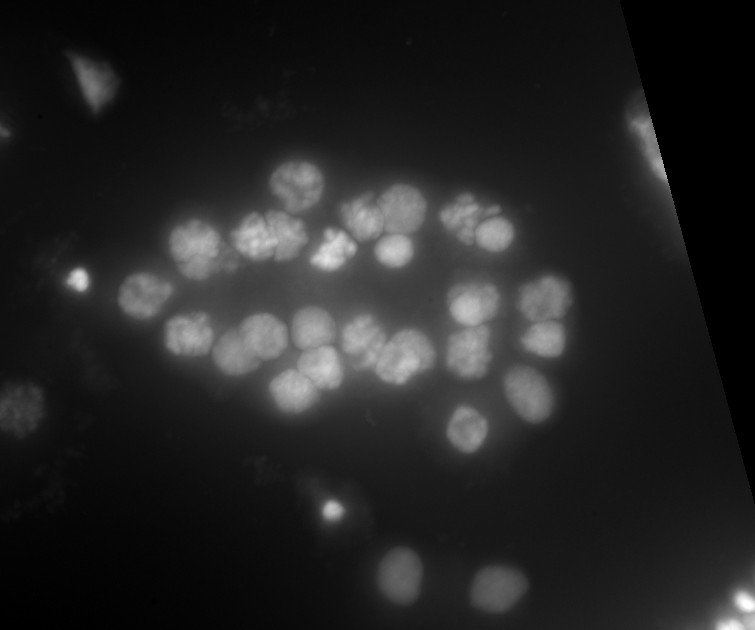

Supplement: Figure 1—source data 1. [file elife-85748-fig1-data1.zip › Figure1-Source_Data1/D-L/MAX_C1 lgg-1(tm) gray Dapi Experiment-6932-tm3489 LGG-1.tif]

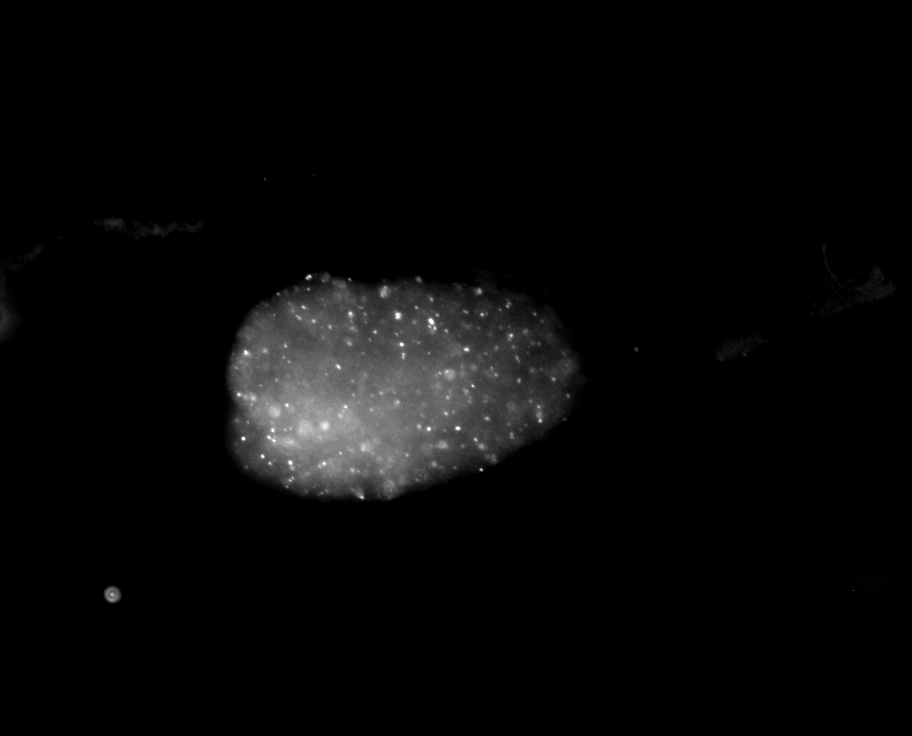

Supplement: Figure 1—source data 1. [file elife-85748-fig1-data1.zip › Figure1-Source_Data1/D-L/N2-100C-AbLGG-1.tif]

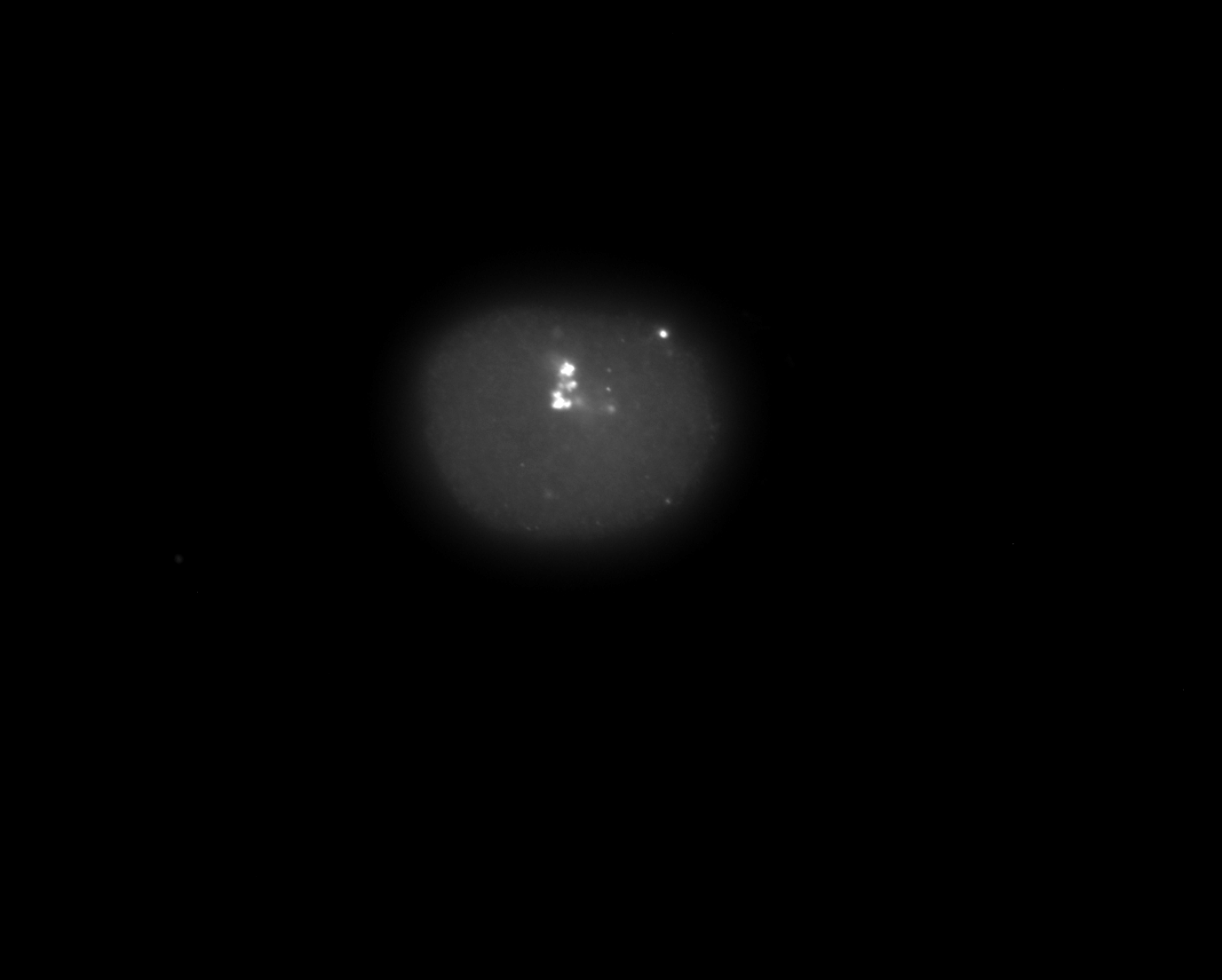

Supplement: Figure 1—source data 1. [file elife-85748-fig1-data1.zip › Figure1-Source_Data1/D-L/N2-précoce-AbLGG-1.tif]

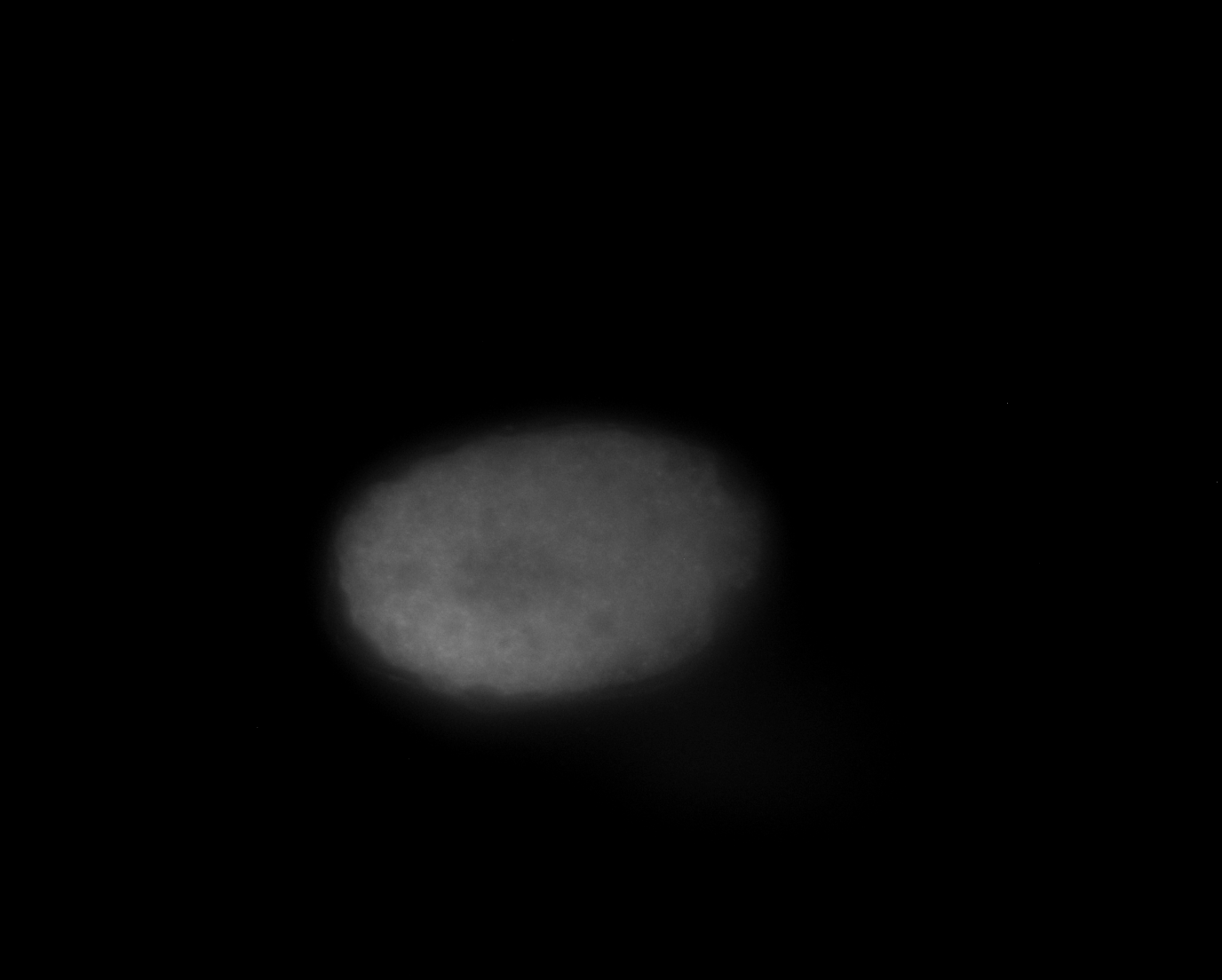

Supplement: Figure 1—source data 1. [file elife-85748-fig1-data1.zip › Figure1-Source_Data1/D-L/pp141-100C-AbLGG-1.tif]

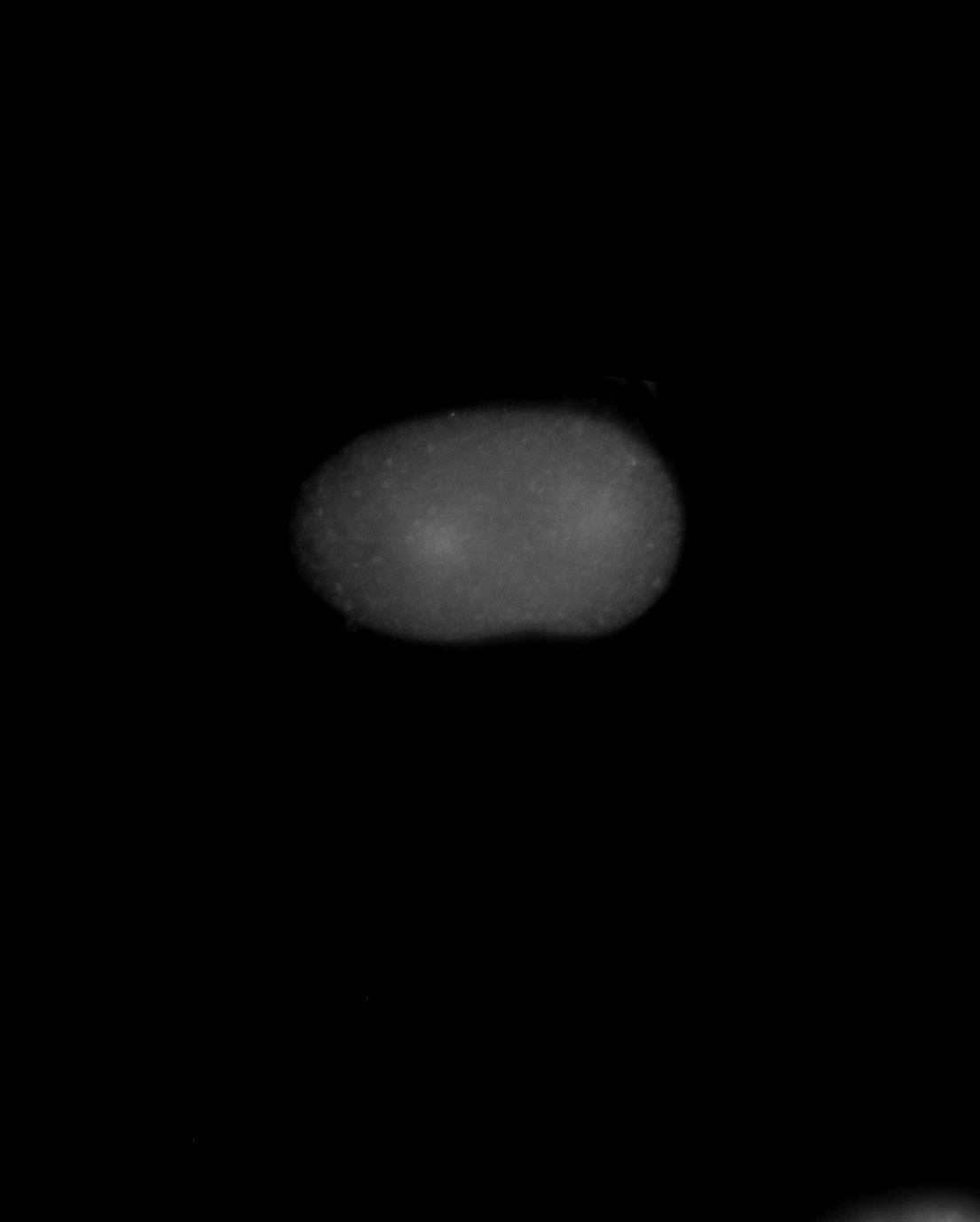

Supplement: Figure 1—source data 1. [file elife-85748-fig1-data1.zip › Figure1-Source_Data1/D-L/pp141-précoce-AbLGG-1.tif]

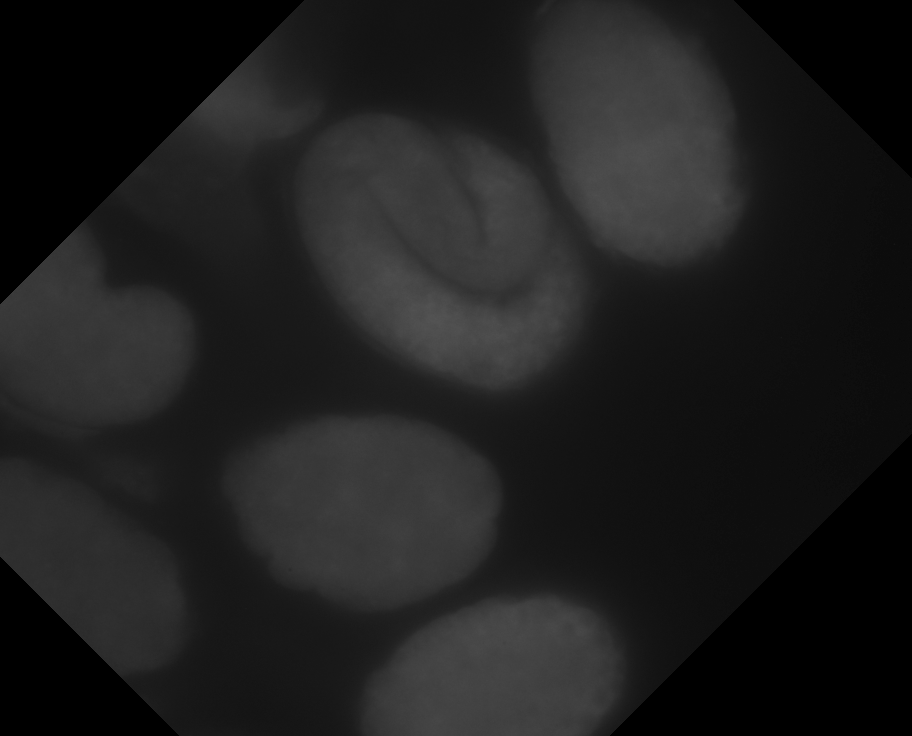

Supplement: Figure 1—source data 1. [file elife-85748-fig1-data1.zip › Figure1-Source_Data1/D-L/pp142-100C-AbLGG-1.tif]

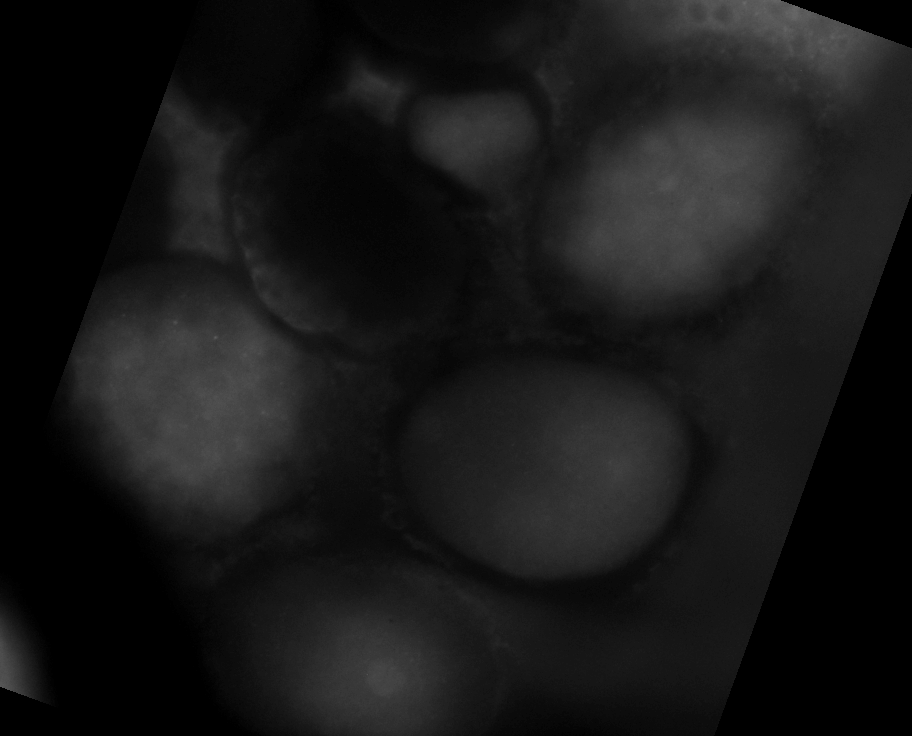

Supplement: Figure 1—source data 1. [file elife-85748-fig1-data1.zip › Figure1-Source_Data1/D-L/pp142-précoce-AbLGG-1.tif]

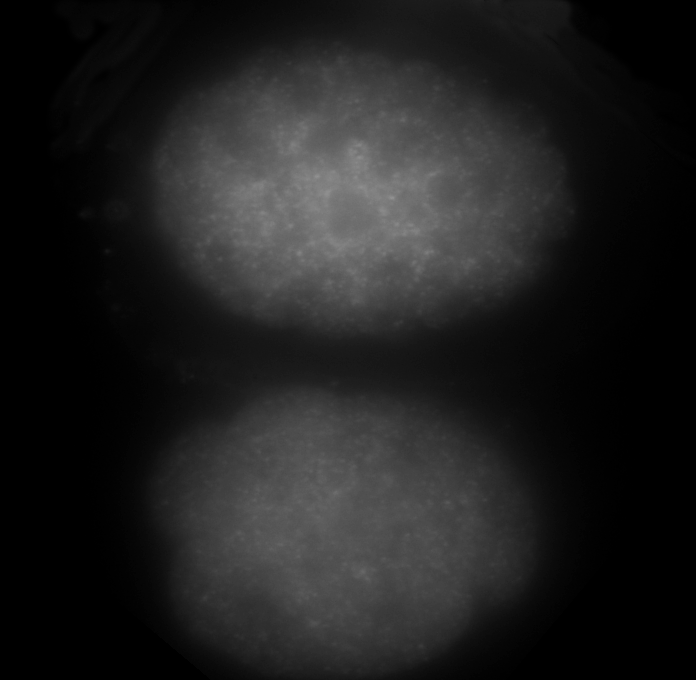

Supplement: Figure 1—source data 1. [file elife-85748-fig1-data1.zip › Figure1-Source_Data1/D-L/pp22-100C-Ab GABARAP.tif]

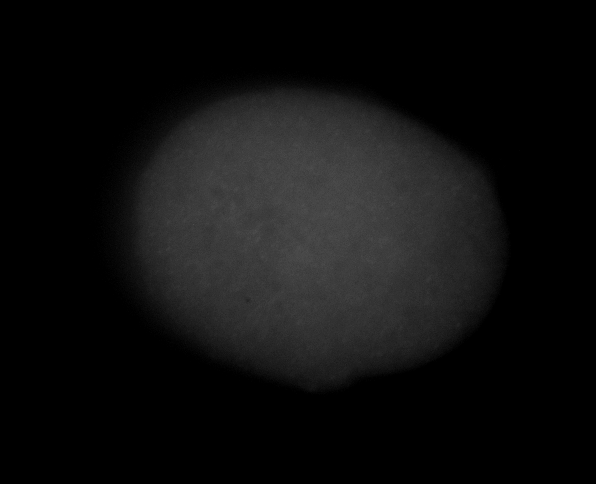

Supplement: Figure 1—source data 1. [file elife-85748-fig1-data1.zip › Figure1-Source_Data1/D-L/pp22-précoce- GABARAP.tif]

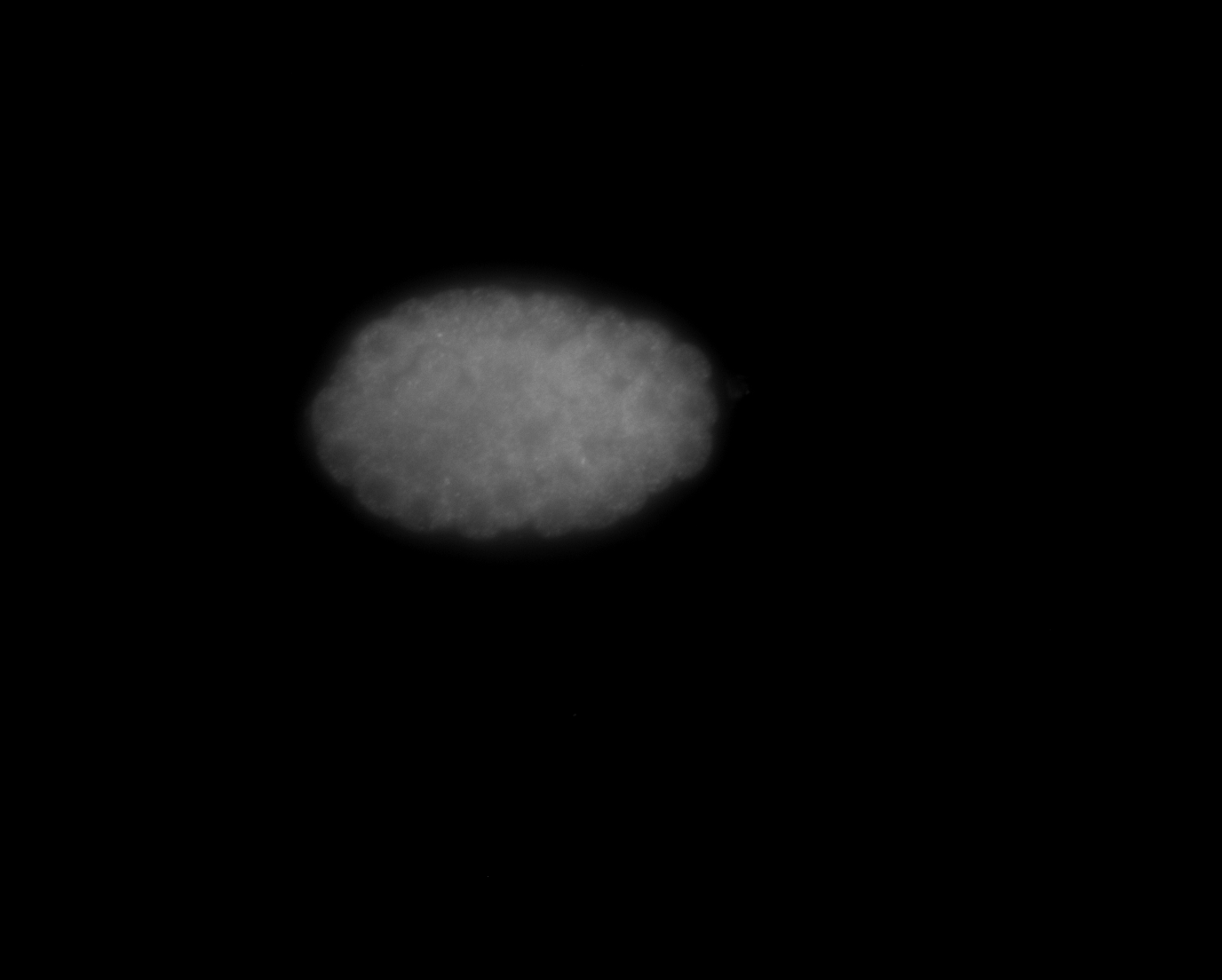

Supplement: Figure 1—source data 1. [file elife-85748-fig1-data1.zip › Figure1-Source_Data1/D-L/pp65-100C-AbLGG-1.tif]

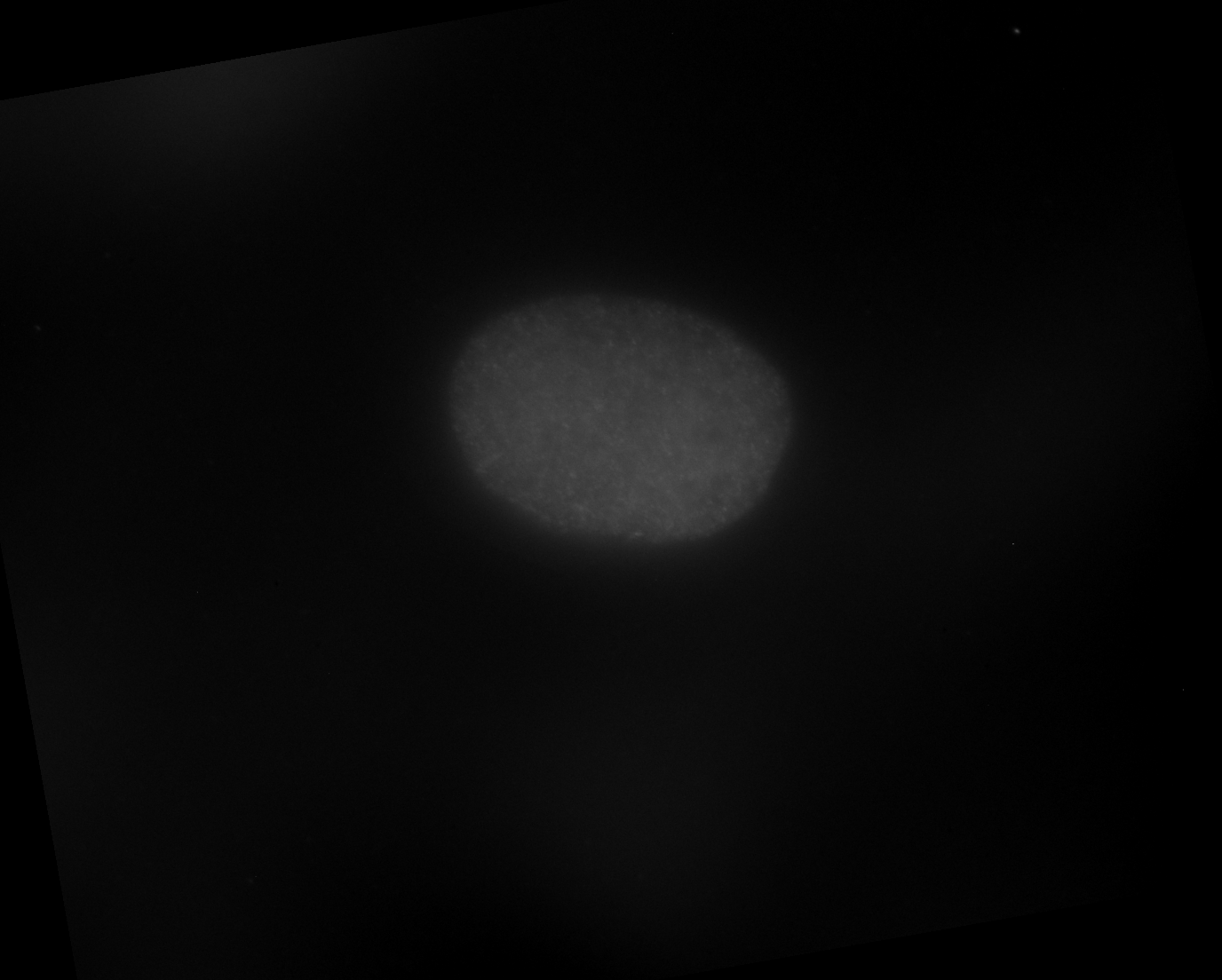

Supplement: Figure 1—source data 1. [file elife-85748-fig1-data1.zip › Figure1-Source_Data1/D-L/pp65-précoce-AbLGG-1.tif]

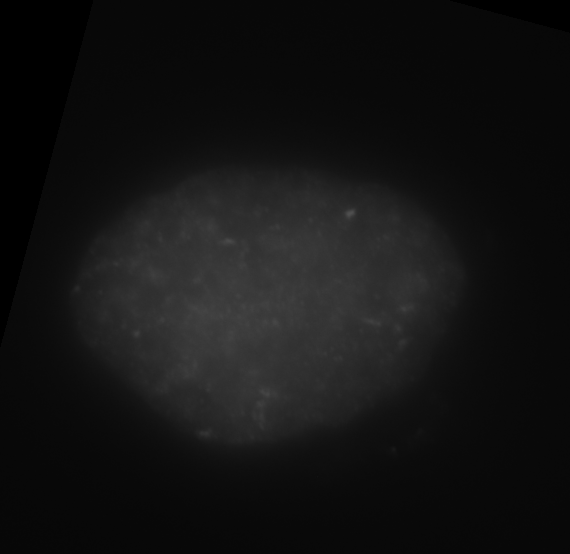

Supplement: Figure 1—source data 1. [file elife-85748-fig1-data1.zip › Figure1-Source_Data1/D-L/pp66-100C_gabarap_6-1.tif]

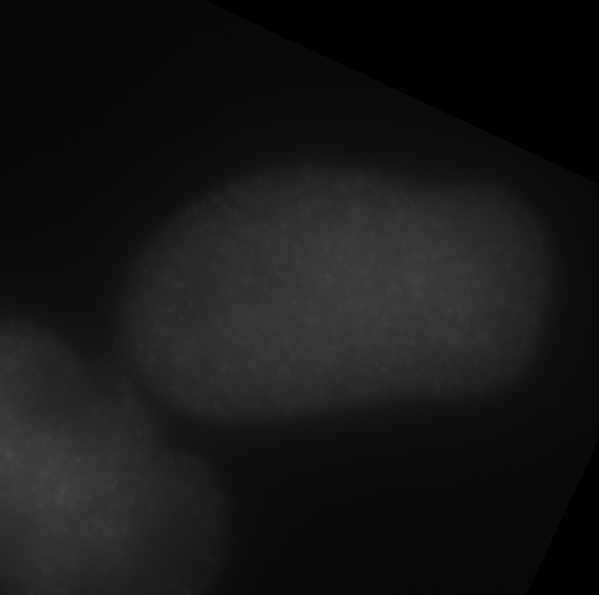

Supplement: Figure 1—source data 1. [file elife-85748-fig1-data1.zip › Figure1-Source_Data1/D-L/pp66-précoce_gabarap_3-2.tif]

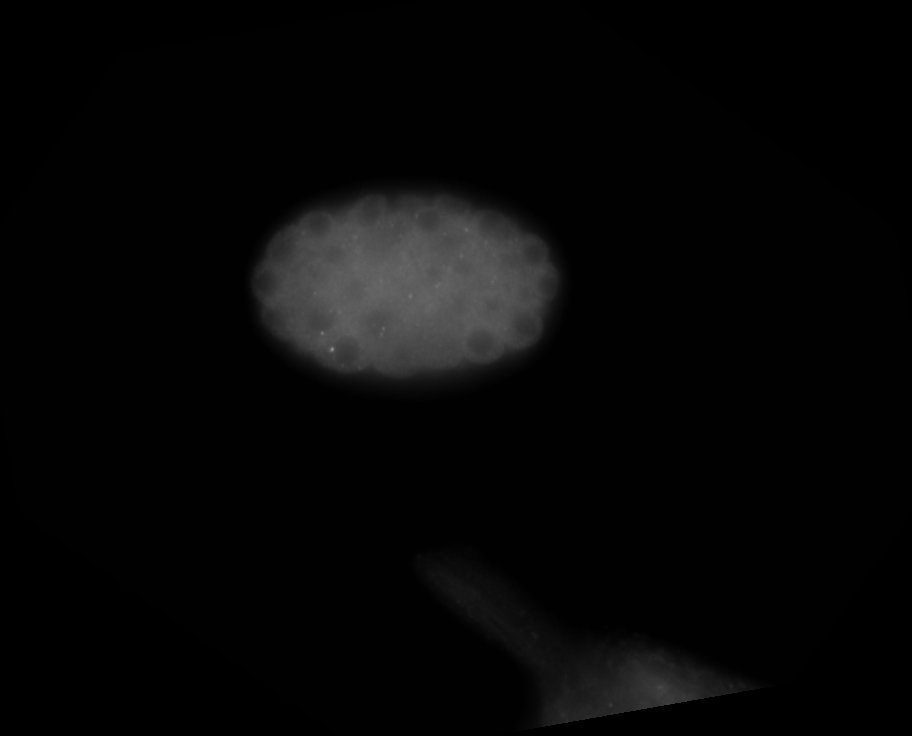

Supplement: Figure 1—figure supplement 2—source data 1. [file elife-85748-fig1-figsupp2-data1.zip › Figure1-figure-supplement2-Source_Data1/D-G/mutant atg.4.1-100C-GABARAP .tif]

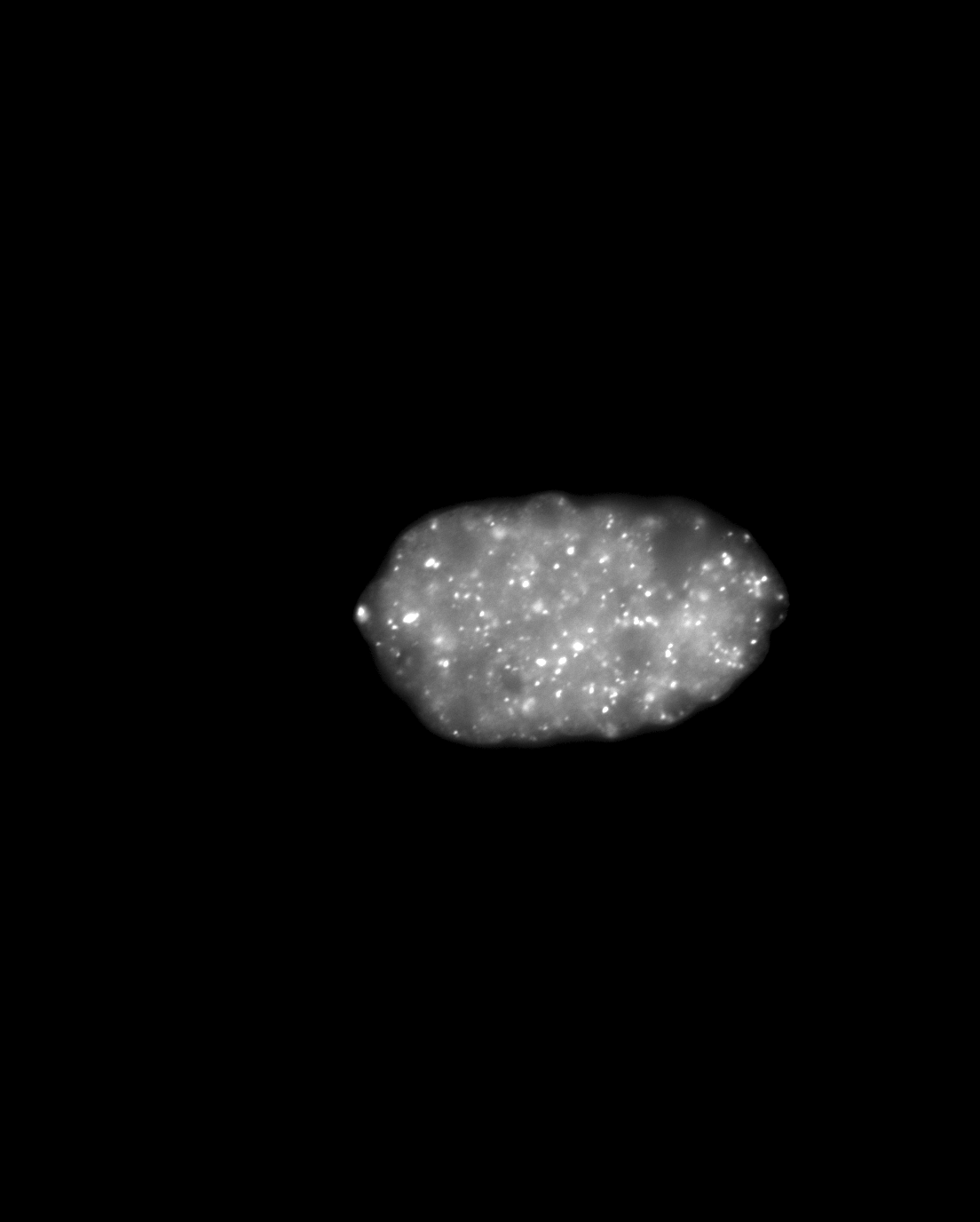

Supplement: Figure 1—figure supplement 2—source data 1. [file elife-85748-fig1-figsupp2-data1.zip › Figure1-figure-supplement2-Source_Data1/D-G/N2-RNAi epg-5.tif]

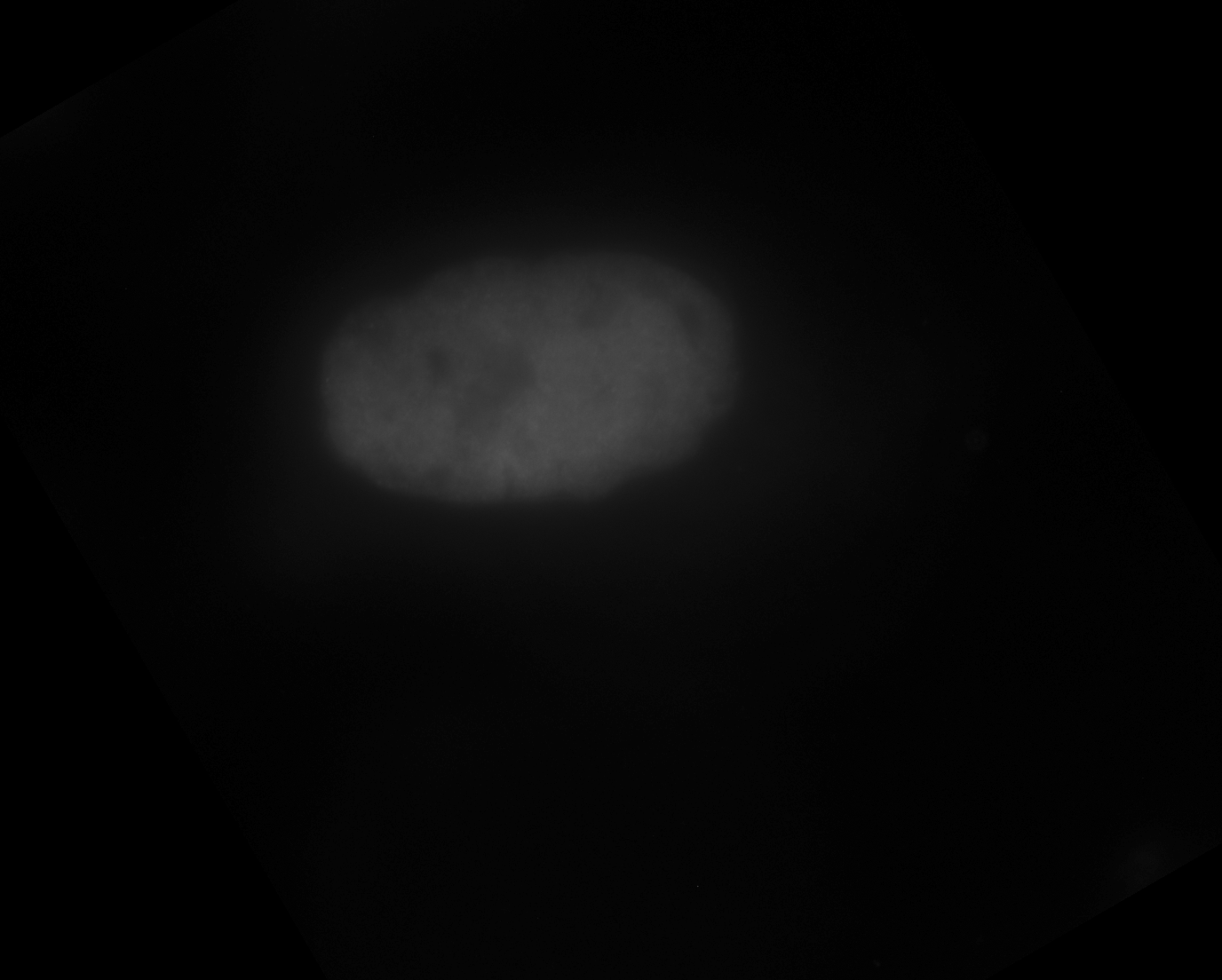

Supplement: Figure 1—figure supplement 2—source data 1. [file elife-85748-fig1-figsupp2-data1.zip › Figure1-figure-supplement2-Source_Data1/D-G/pp141-RNAi epg-5.tif]

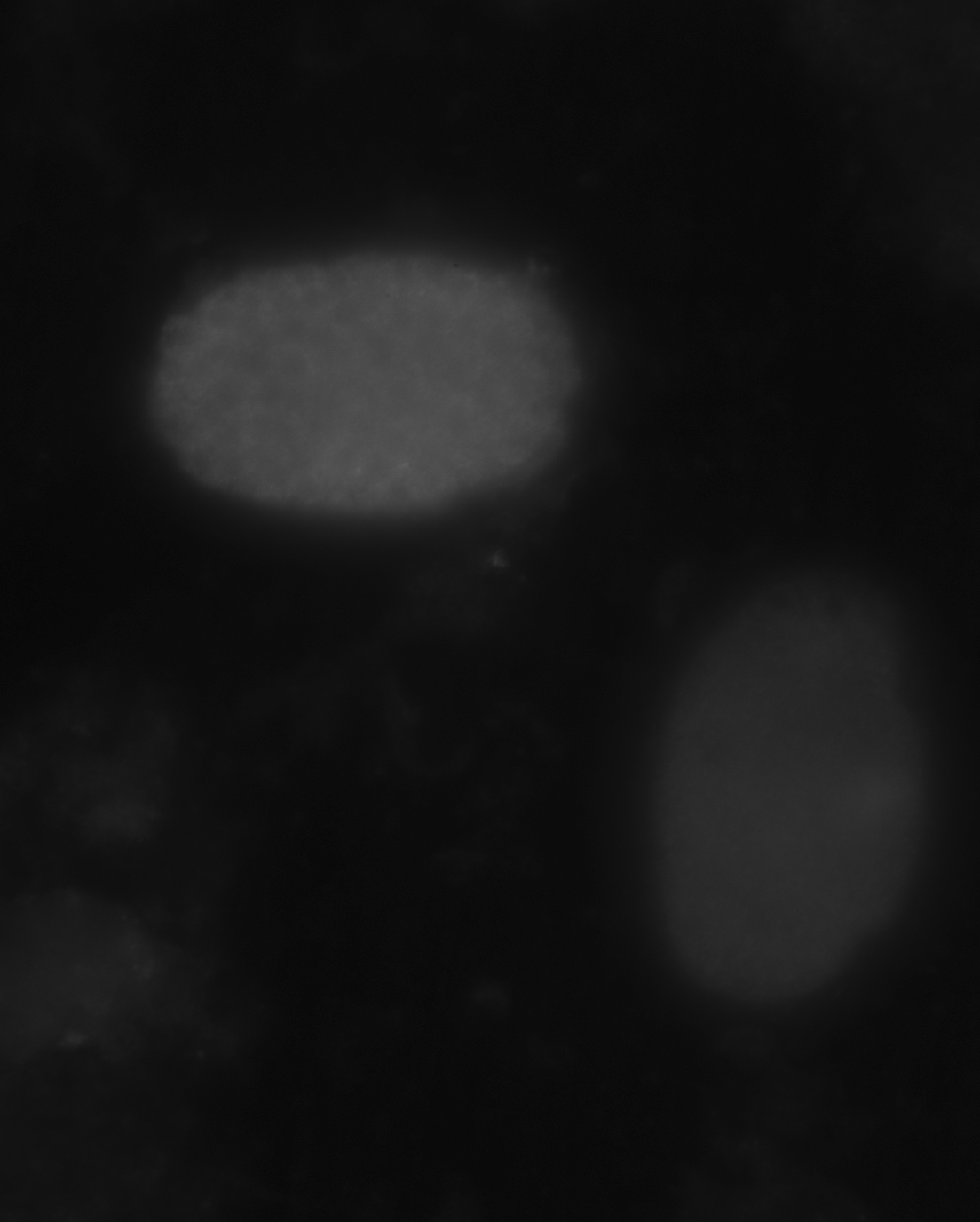

Supplement: Figure 1—figure supplement 2—source data 1. [file elife-85748-fig1-figsupp2-data1.zip › Figure1-figure-supplement2-Source_Data1/D-G/pp65-RNAi epg-5.tif]

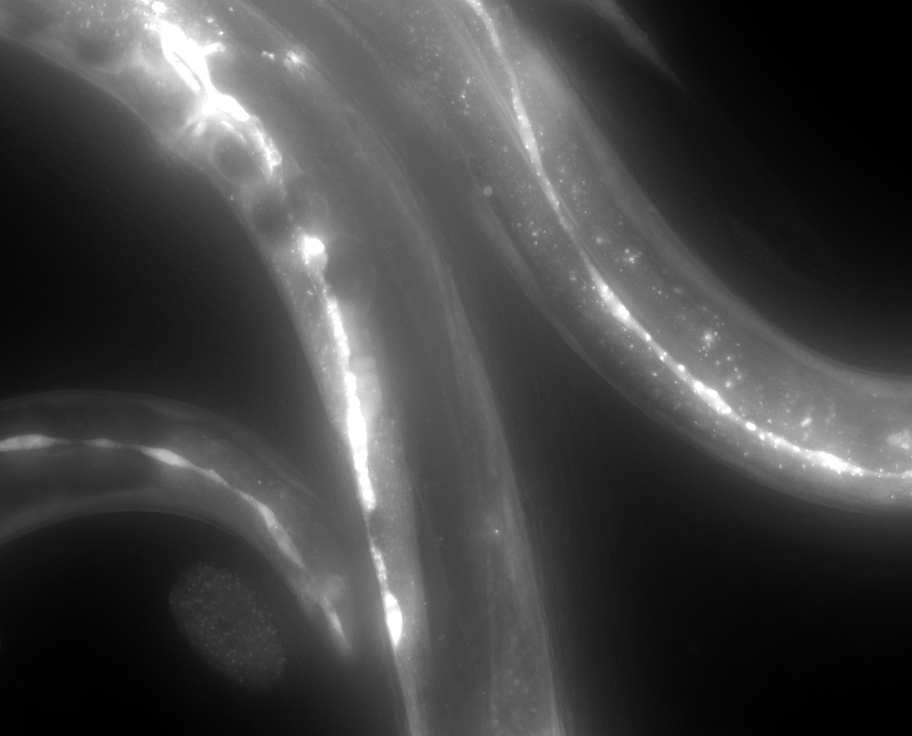

Supplement: Figure 1—figure supplement 3—source data 1. [file elife-85748-fig1-figsupp3-data1.zip › Figure1-figuresuplement3-Source_Data1/C-D/DA2123-MAX_1-15-epg5 20.tif]

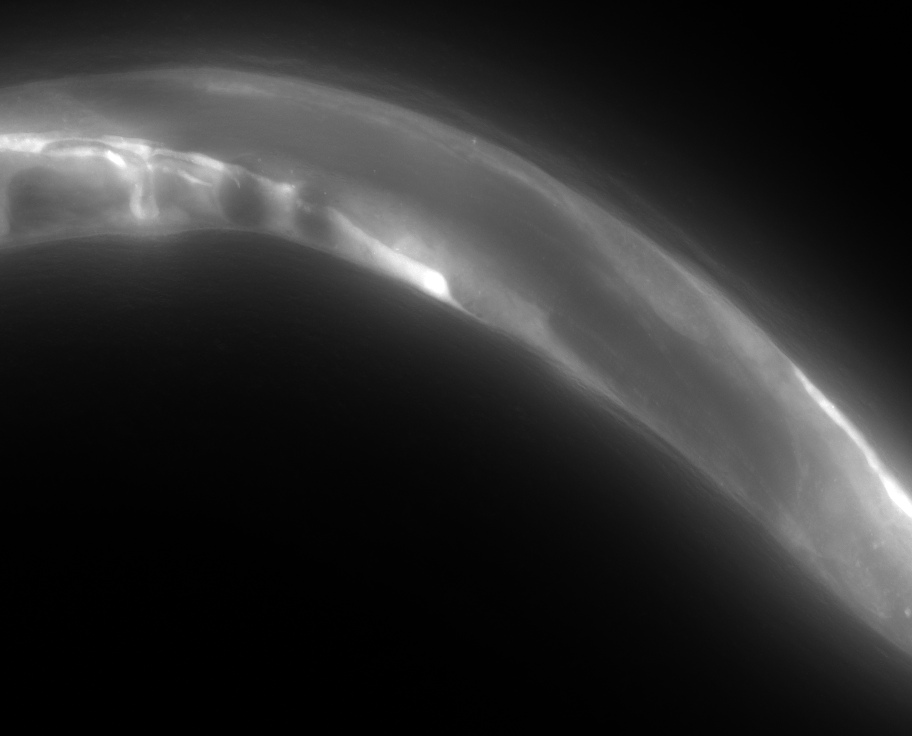

Supplement: Figure 1—figure supplement 3—source data 1. [file elife-85748-fig1-figsupp3-data1.zip › Figure1-figuresuplement3-Source_Data1/C-D/DA2123-MAX_2-16-atg3 20.tif]

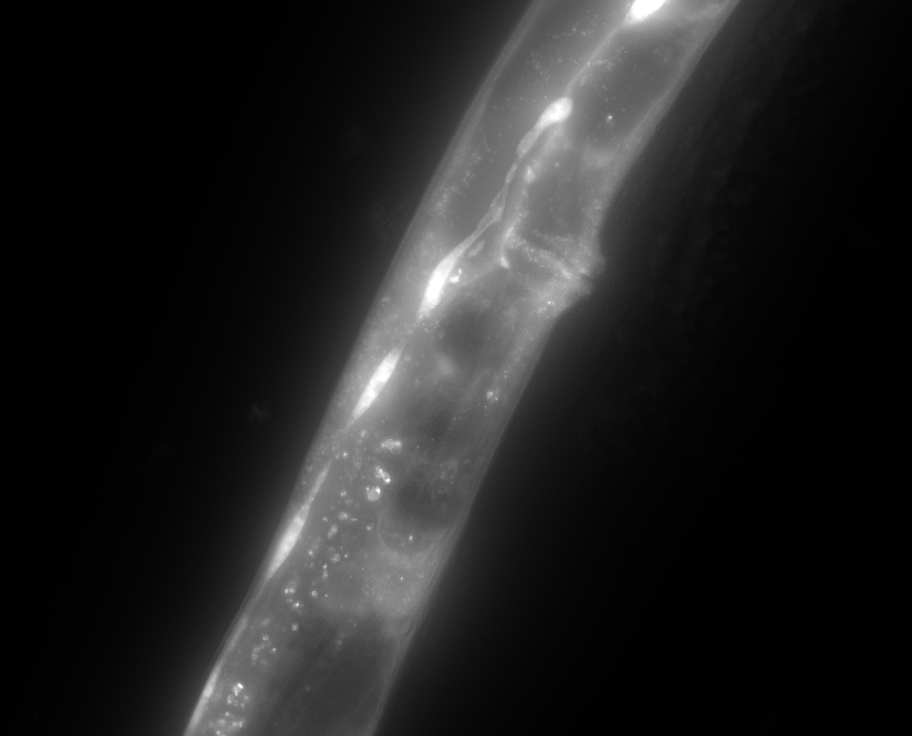

Supplement: Figure 1—figure supplement 3—source data 1. [file elife-85748-fig1-figsupp3-data1.zip › Figure1-figuresuplement3-Source_Data1/C-D/DA2123-MAX_2-16-l4440 37.tif]

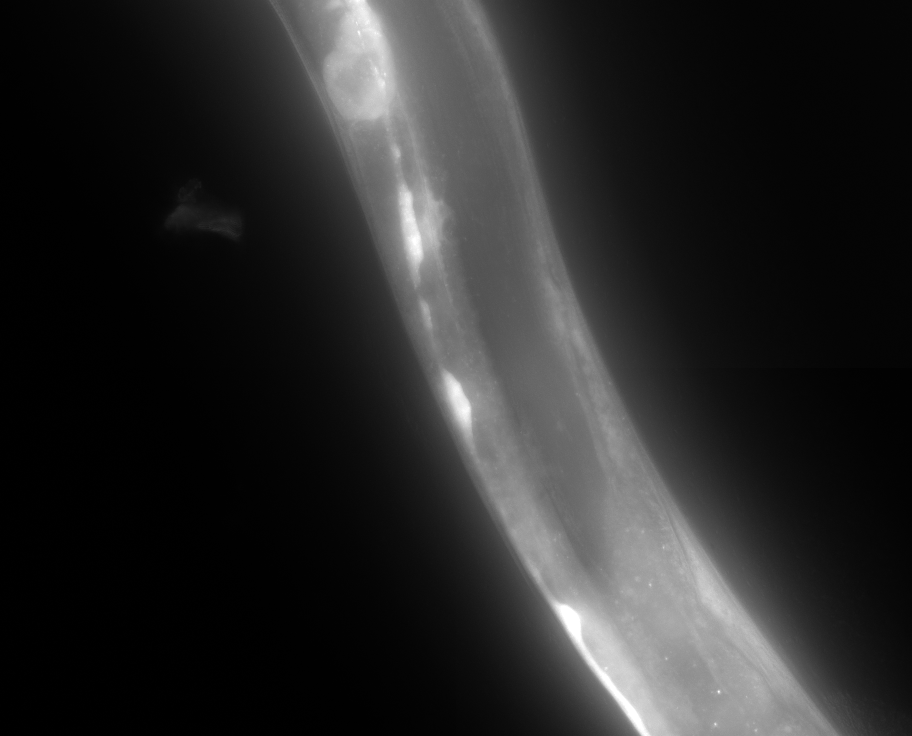

Supplement: Figure 1—figure supplement 3—source data 1. [file elife-85748-fig1-figsupp3-data1.zip › Figure1-figuresuplement3-Source_Data1/C-D/DA2123-MAX_2-17-l4440 20.tif]

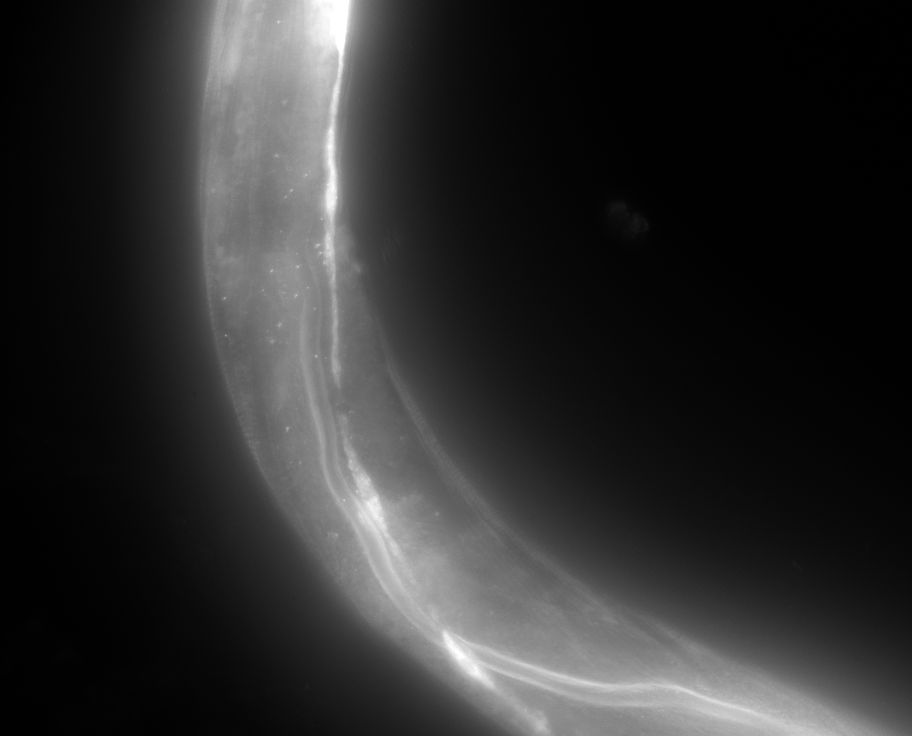

Supplement: Figure 1—figure supplement 3—source data 1. [file elife-85748-fig1-figsupp3-data1.zip › Figure1-figuresuplement3-Source_Data1/C-D/DA2123-MAX_5-16-atg7 20.tif]

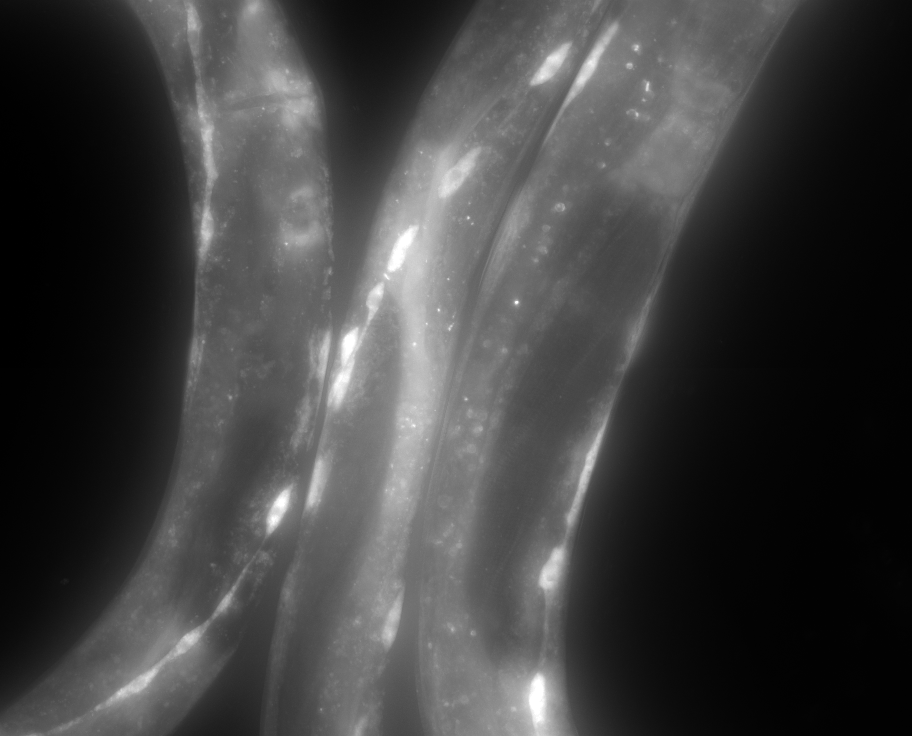

Supplement: Figure 1—figure supplement 3—source data 1. [file elife-85748-fig1-figsupp3-data1.zip › Figure1-figuresuplement3-Source_Data1/C-D/DA2123-MAX_5-20-atg7 37-1.tif]

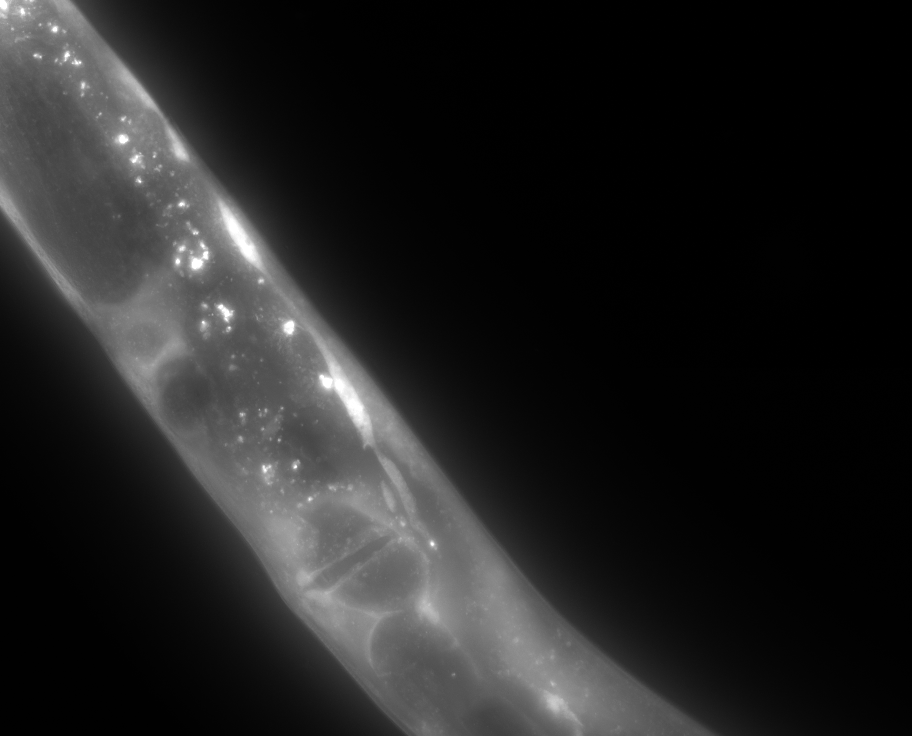

Supplement: Figure 1—figure supplement 3—source data 1. [file elife-85748-fig1-figsupp3-data1.zip › Figure1-figuresuplement3-Source_Data1/C-D/DA2123-MAX_5-20-epg5 37'.tif]

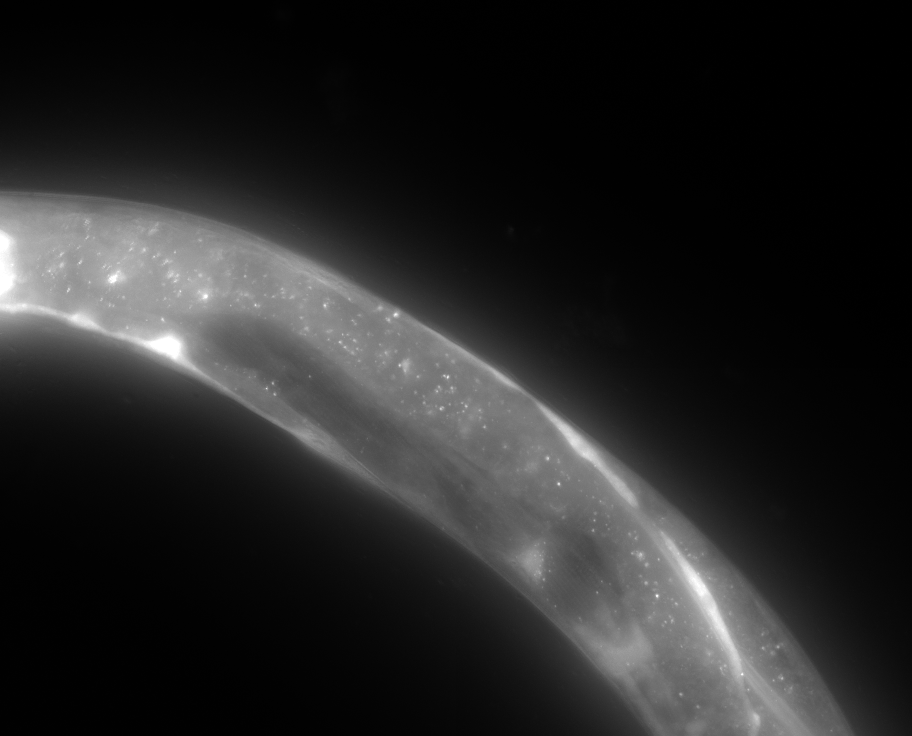

Supplement: Figure 1—figure supplement 3—source data 1. [file elife-85748-fig1-figsupp3-data1.zip › Figure1-figuresuplement3-Source_Data1/C-D/DA2123-MAX_5-20-rab 7 20.tif]

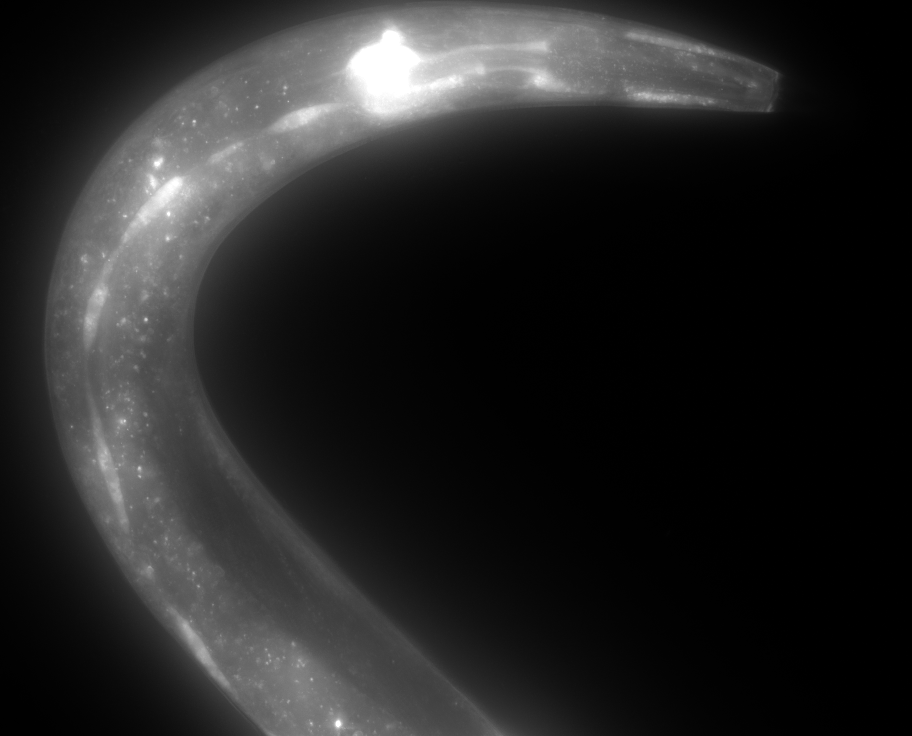

Supplement: Figure 1—figure supplement 3—source data 1. [file elife-85748-fig1-figsupp3-data1.zip › Figure1-figuresuplement3-Source_Data1/C-D/DA2123-MAX_5-20-rab7 37'.tif]

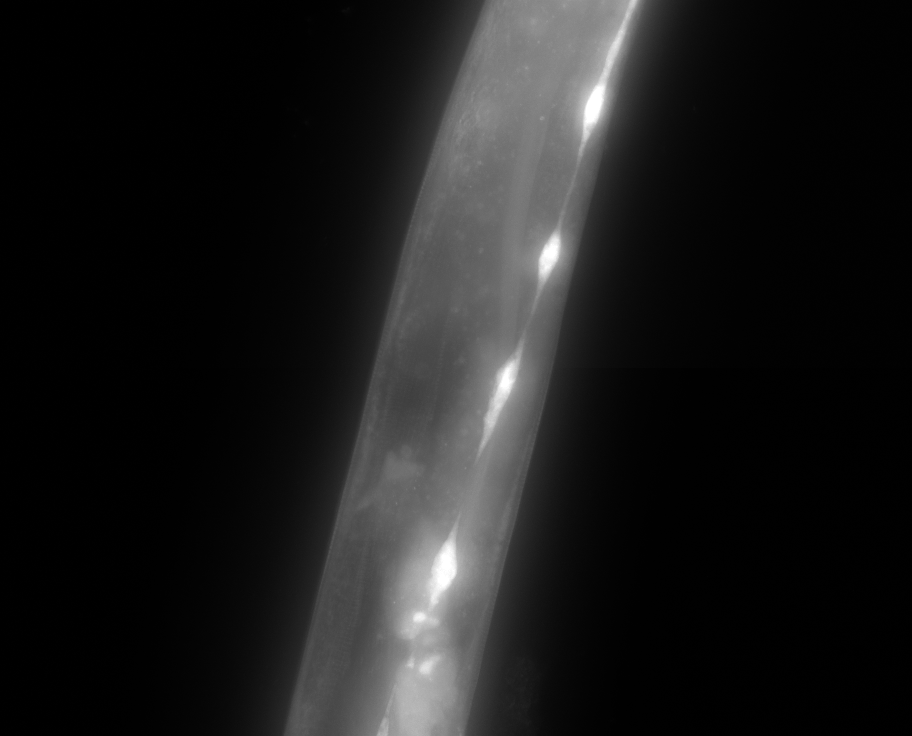

Supplement: Figure 1—figure supplement 3—source data 1. [file elife-85748-fig1-figsupp3-data1.zip › Figure1-figuresuplement3-Source_Data1/C-D/DA2123-MAX_6-20-atg3 37'.tif]

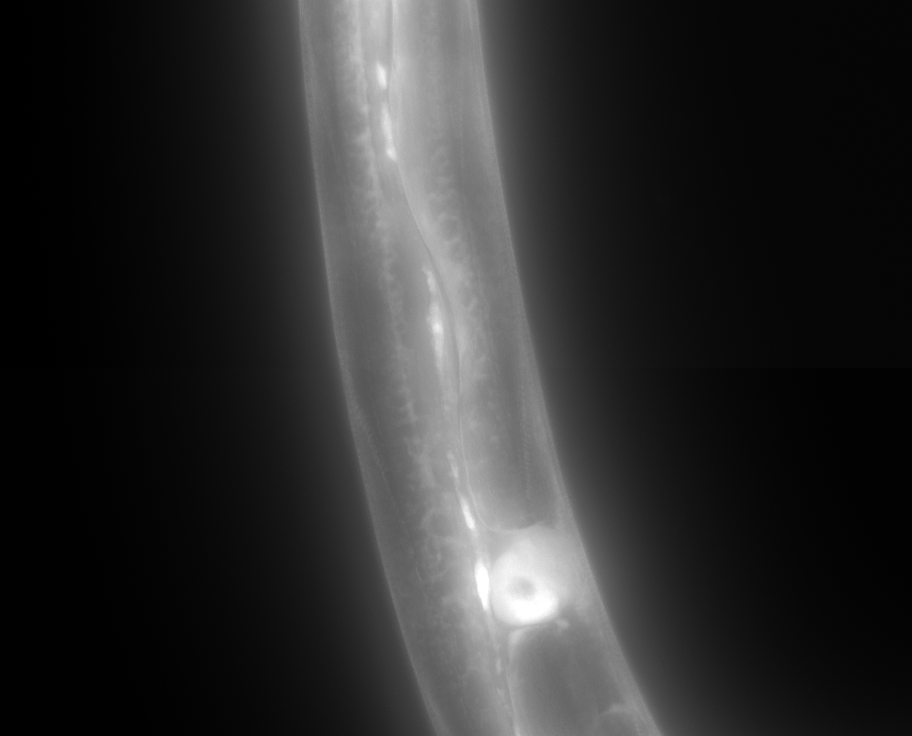

Supplement: Figure 1—figure supplement 3—source data 1. [file elife-85748-fig1-figsupp3-data1.zip › Figure1-figuresuplement3-Source_Data1/C-D/RD202-MAX_10-24-l4440 20.tif]

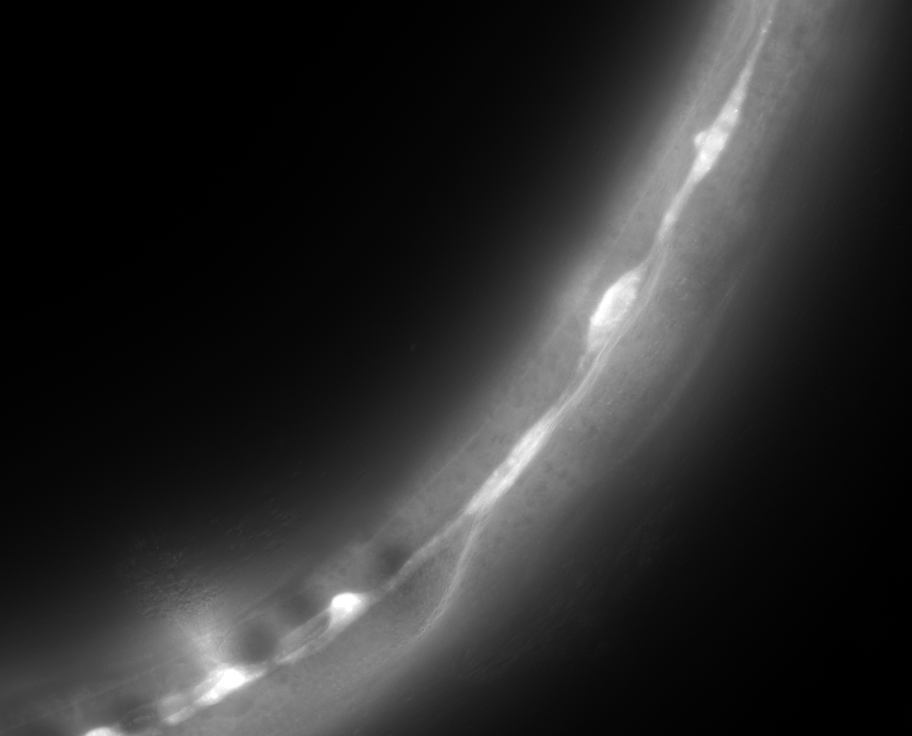

Supplement: Figure 1—figure supplement 3—source data 1. [file elife-85748-fig1-figsupp3-data1.zip › Figure1-figuresuplement3-Source_Data1/C-D/RD202-MAX_1-5-epg5 20-2.tif]

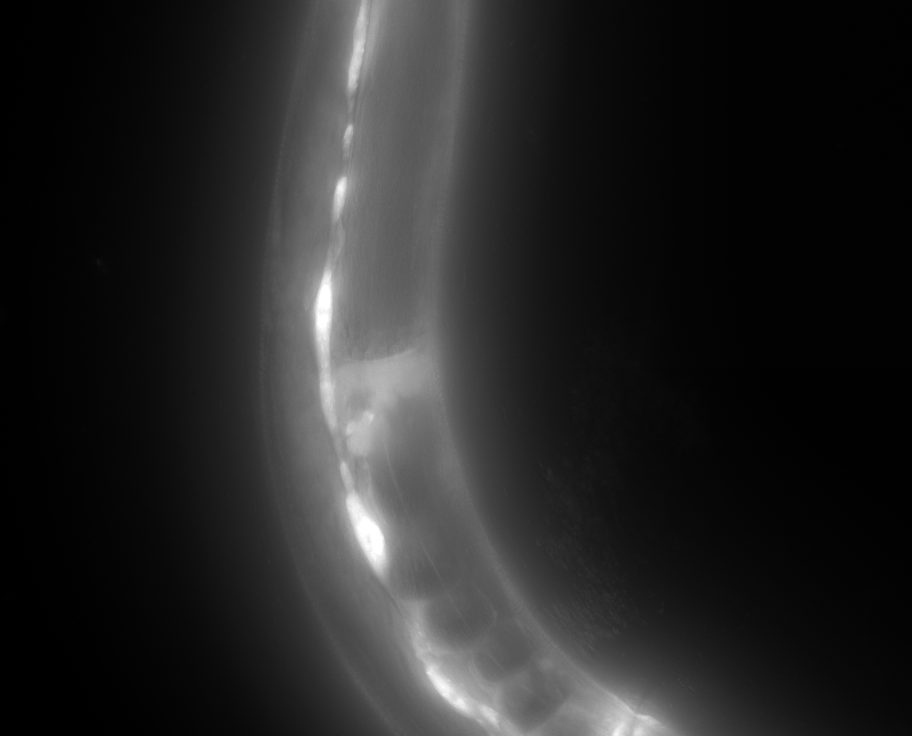

Supplement: Figure 1—figure supplement 3—source data 1. [file elife-85748-fig1-figsupp3-data1.zip › Figure1-figuresuplement3-Source_Data1/C-D/RD202-MAX_4-14-rab7 20.tif]

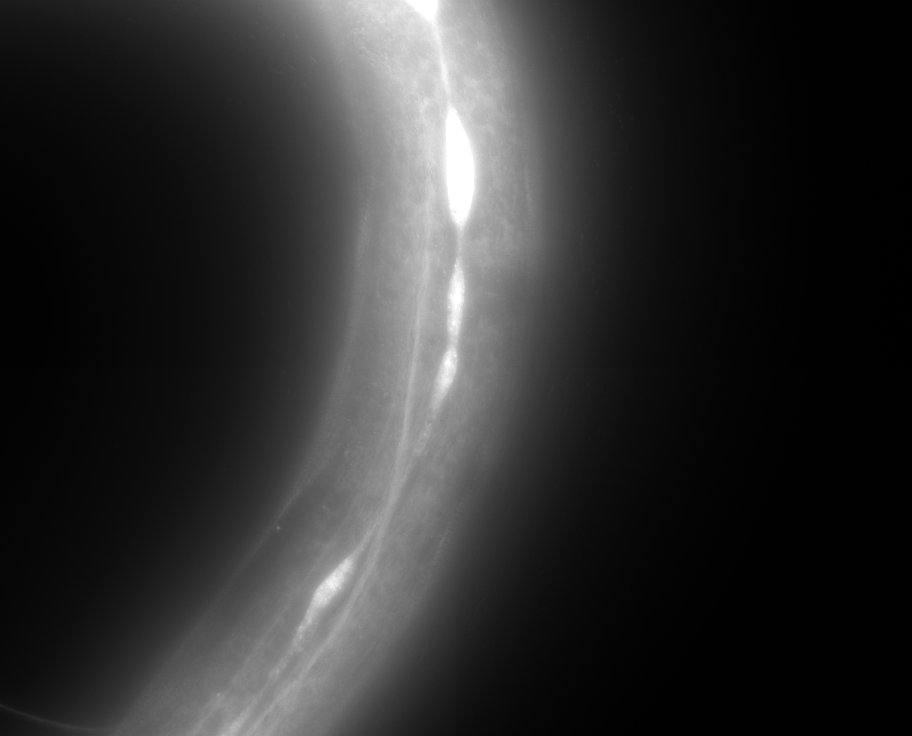

Supplement: Figure 1—figure supplement 3—source data 1. [file elife-85748-fig1-figsupp3-data1.zip › Figure1-figuresuplement3-Source_Data1/C-D/RD202-MAX_5-12-epg5 37.tif]

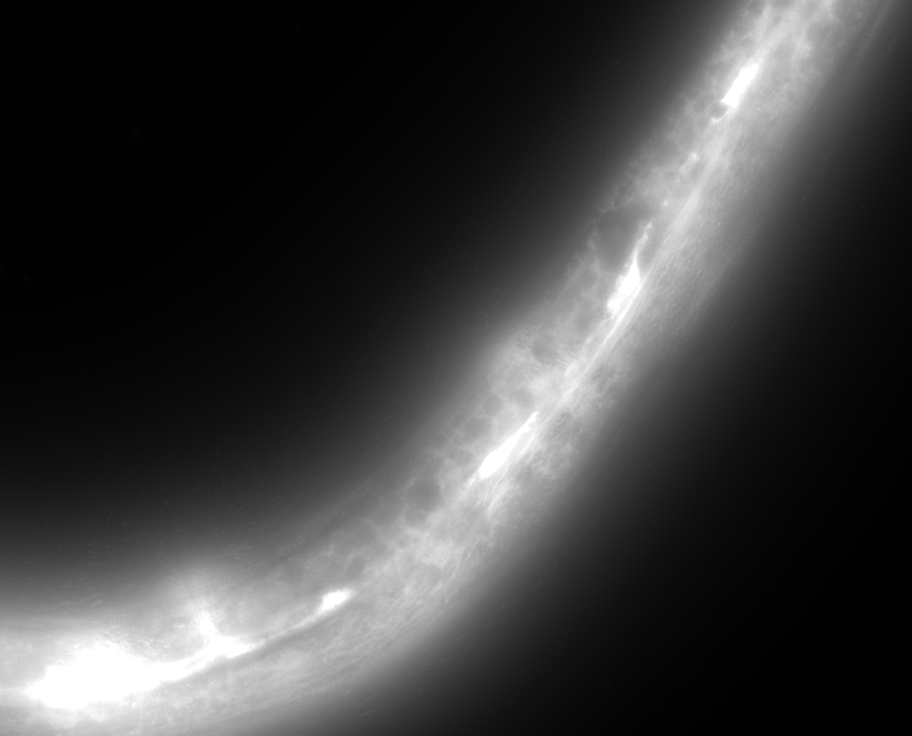

Supplement: Figure 1—figure supplement 3—source data 1. [file elife-85748-fig1-figsupp3-data1.zip › Figure1-figuresuplement3-Source_Data1/C-D/RD202-MAX_5-12-rab7 37'.tif]

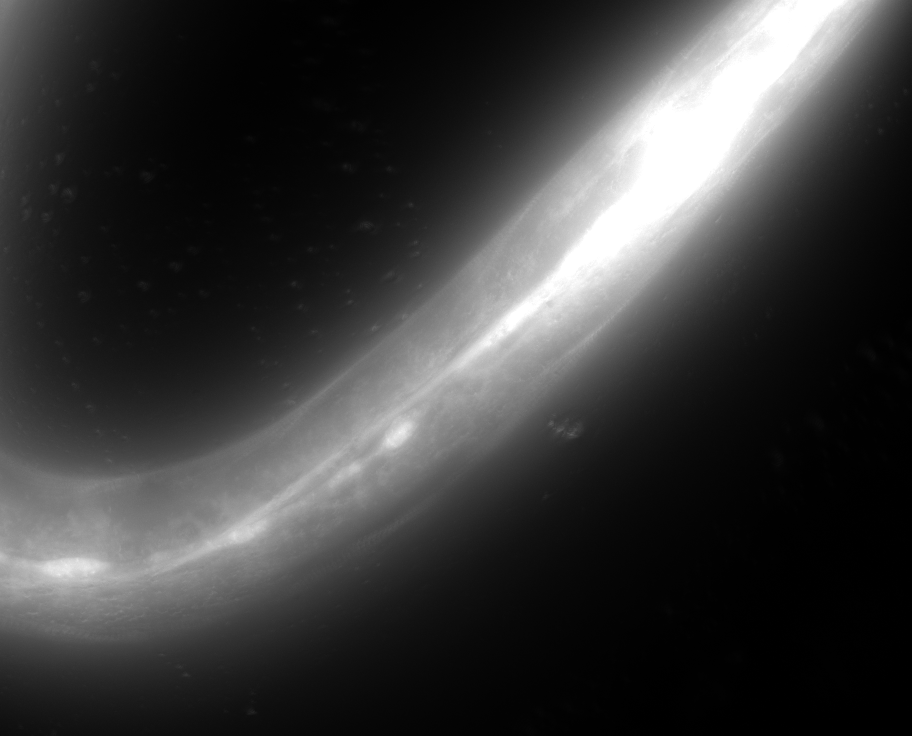

Supplement: Figure 1—figure supplement 3—source data 1. [file elife-85748-fig1-figsupp3-data1.zip › Figure1-figuresuplement3-Source_Data1/C-D/RD202-MAX_5-14-l4440 37-2.tif]

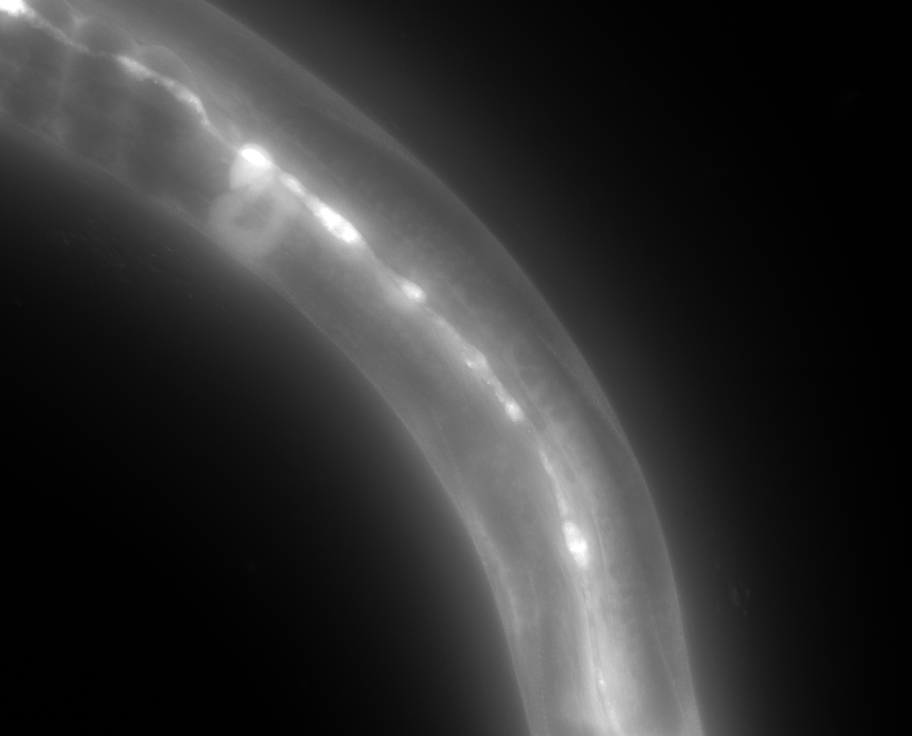

Supplement: Figure 1—figure supplement 3—source data 1. [file elife-85748-fig1-figsupp3-data1.zip › Figure1-figuresuplement3-Source_Data1/C-D/RD202-MAX_5-17-atg3 20.tif]

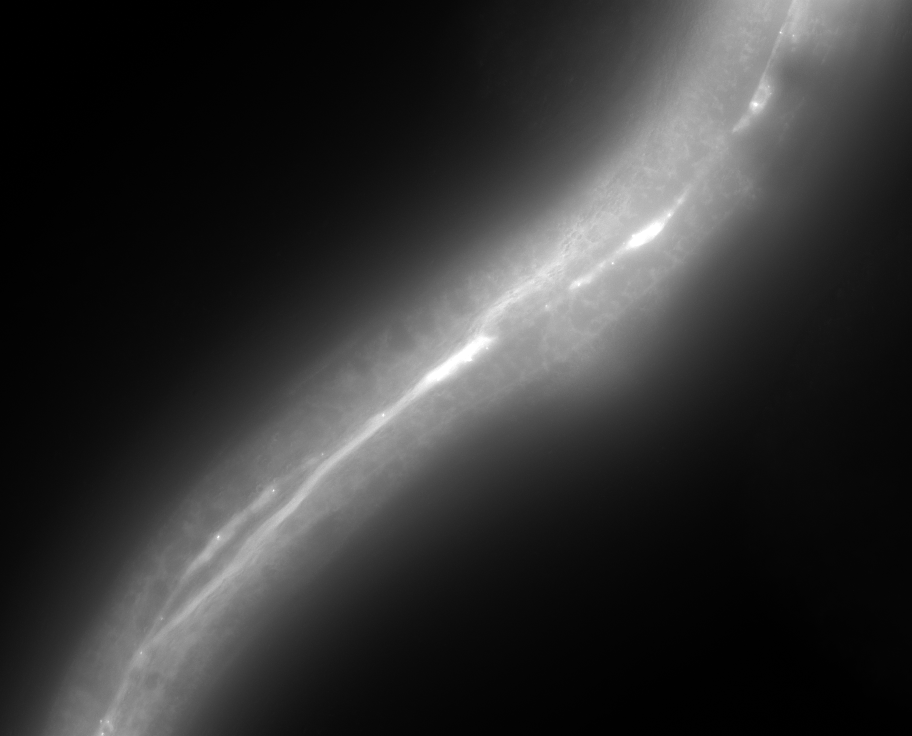

Supplement: Figure 1—figure supplement 3—source data 1. [file elife-85748-fig1-figsupp3-data1.zip › Figure1-figuresuplement3-Source_Data1/C-D/RD202-MAX_6-12-atg7 20.tif]

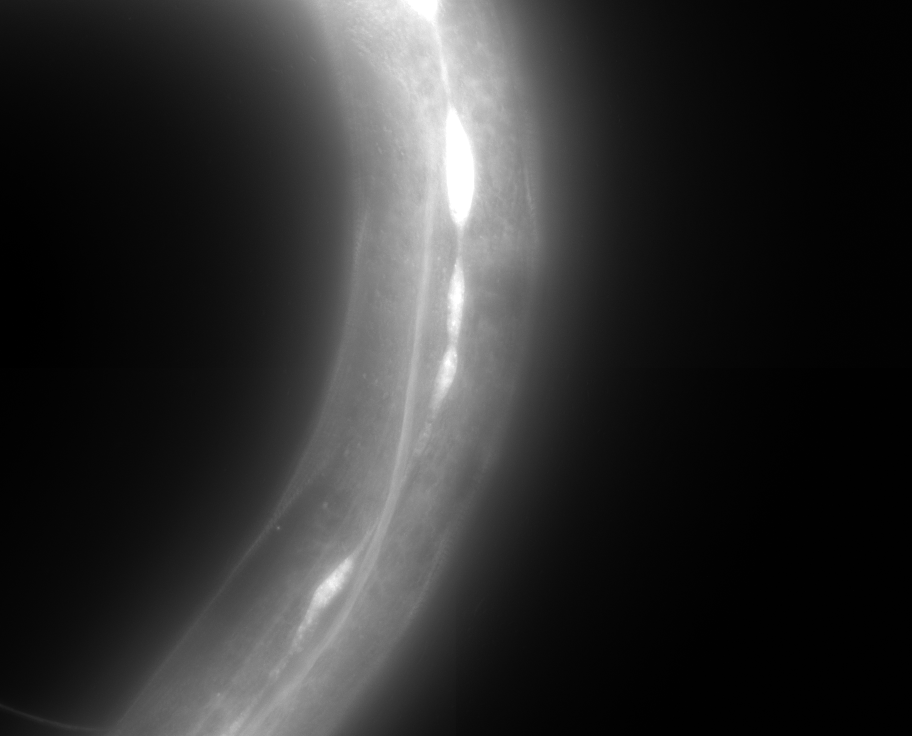

Supplement: Figure 1—figure supplement 3—source data 1. [file elife-85748-fig1-figsupp3-data1.zip › Figure1-figuresuplement3-Source_Data1/C-D/RD202-MAX_6-14-epg5 37.tif]

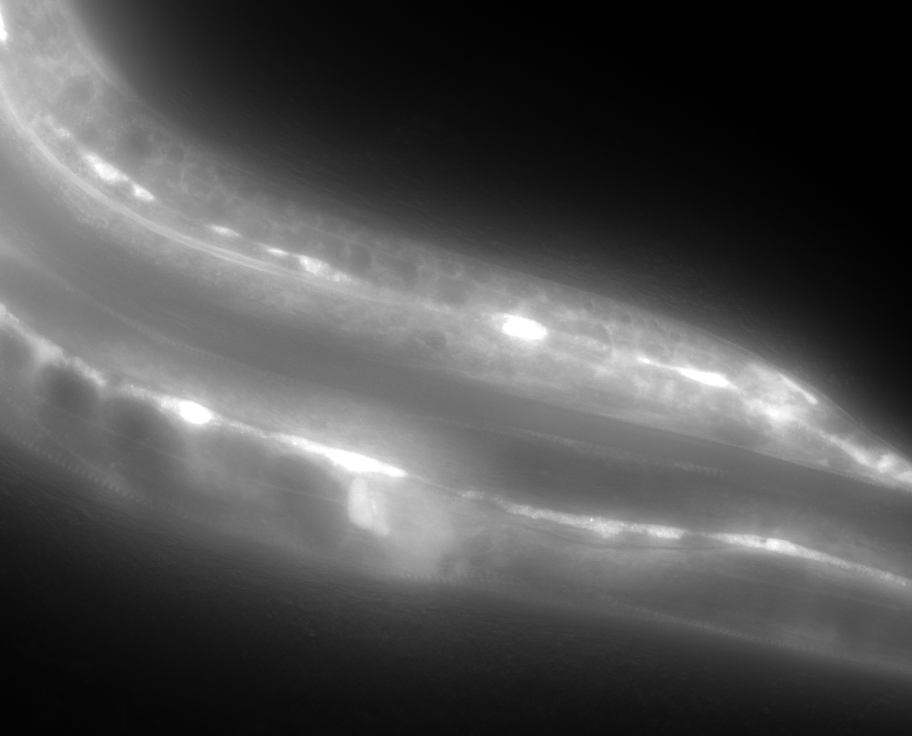

Supplement: Figure 1—figure supplement 3—source data 1. [file elife-85748-fig1-figsupp3-data1.zip › Figure1-figuresuplement3-Source_Data1/C-D/RD202-MAX_6-16-rab7 37.tif]

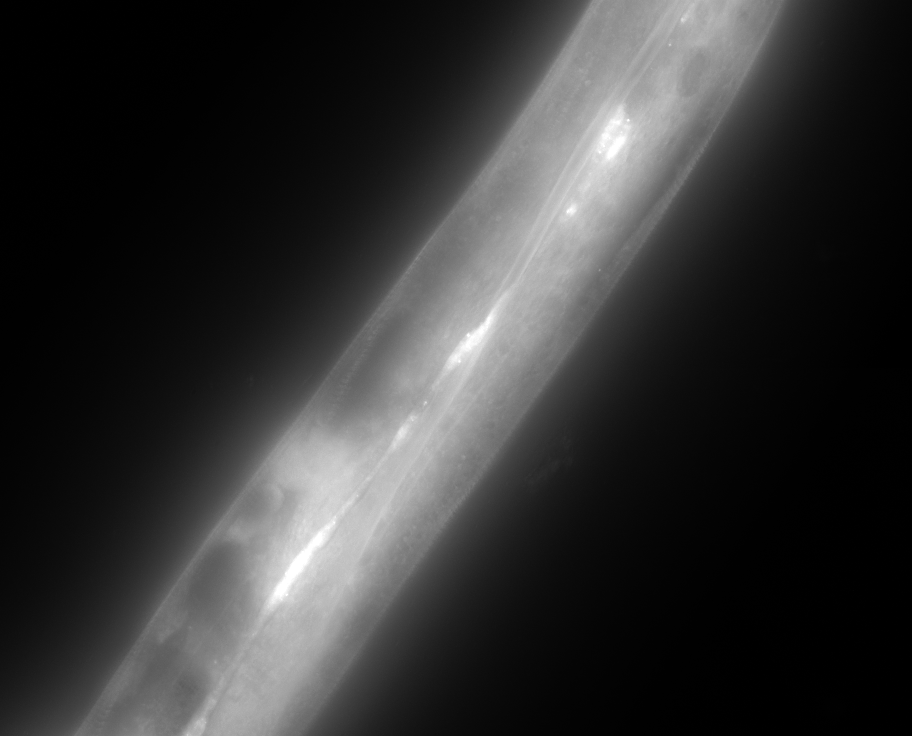

Supplement: Figure 1—figure supplement 3—source data 1. [file elife-85748-fig1-figsupp3-data1.zip › Figure1-figuresuplement3-Source_Data1/C-D/RD202-MAX_6-17-atg3 37'.tif]

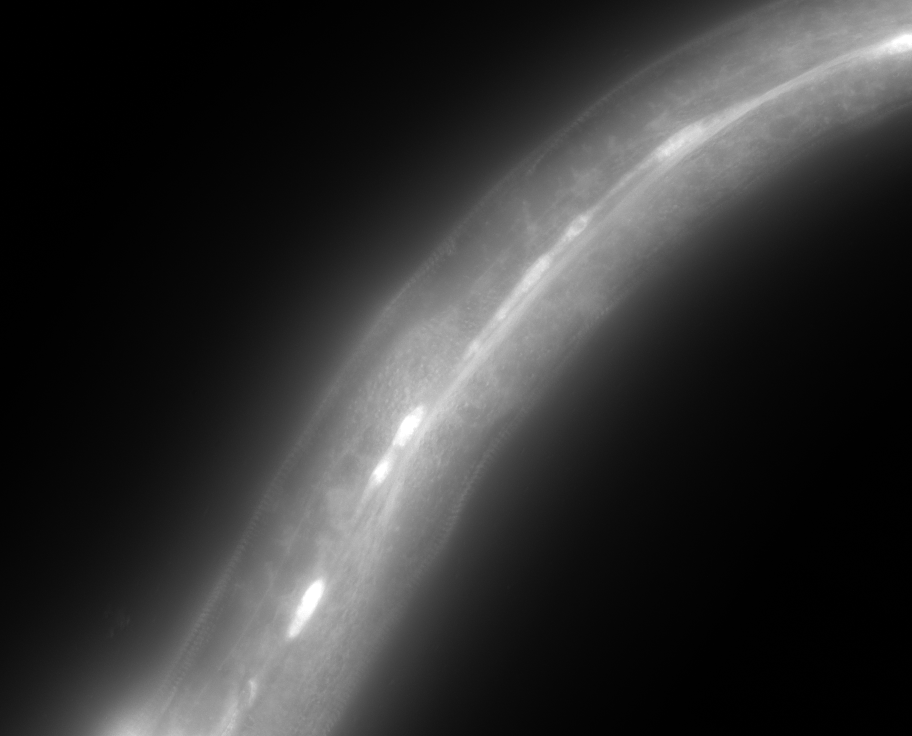

Supplement: Figure 1—figure supplement 3—source data 1. [file elife-85748-fig1-figsupp3-data1.zip › Figure1-figuresuplement3-Source_Data1/C-D/RD202-MAX_7-16-atg7 37.tif]

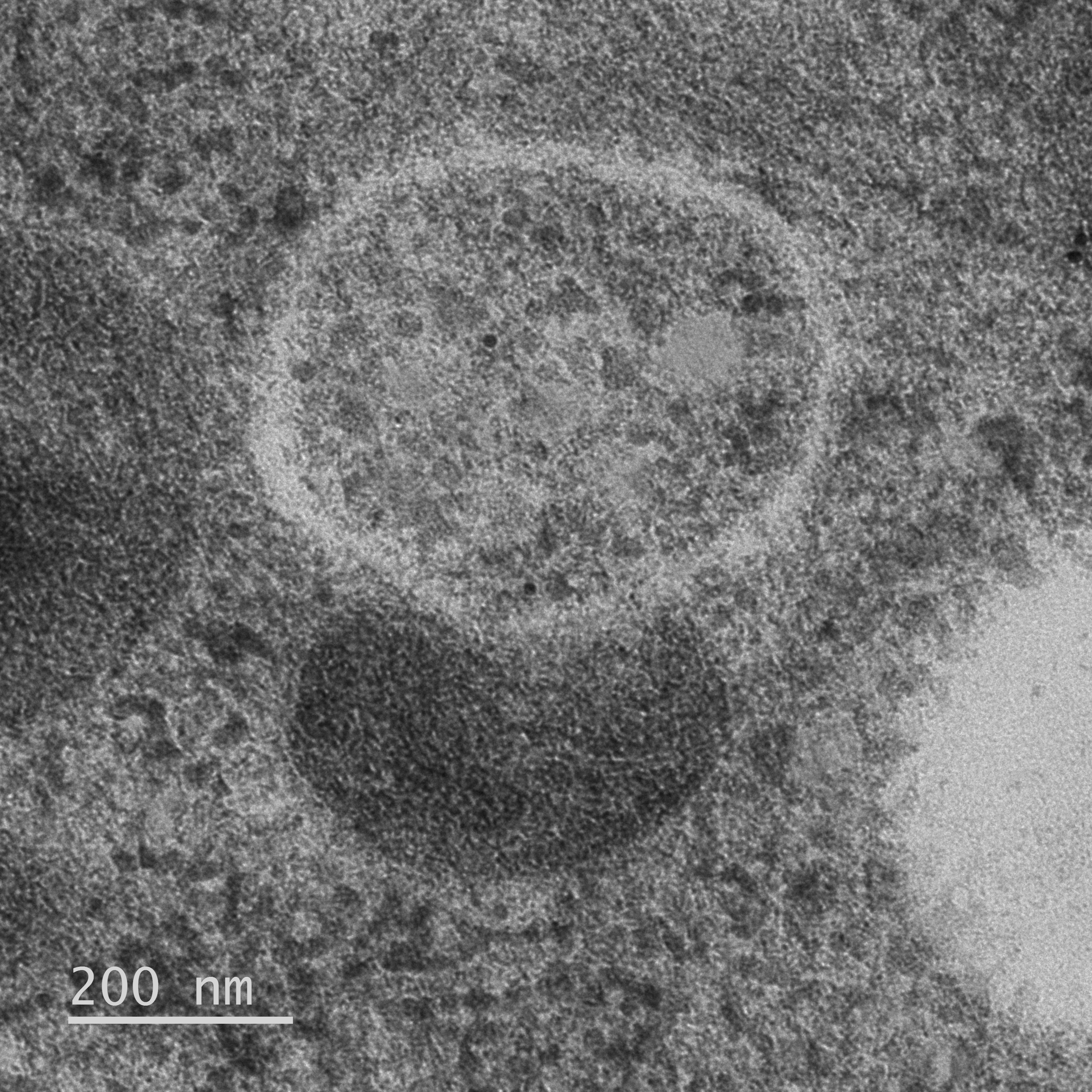

Supplement: Figure 1—figure supplement 3—source data 1. [file elife-85748-fig1-figsupp3-data1.zip › Figure1-figuresuplement3-Source_Data1/E-F/18042023_RD202_0062.tif]

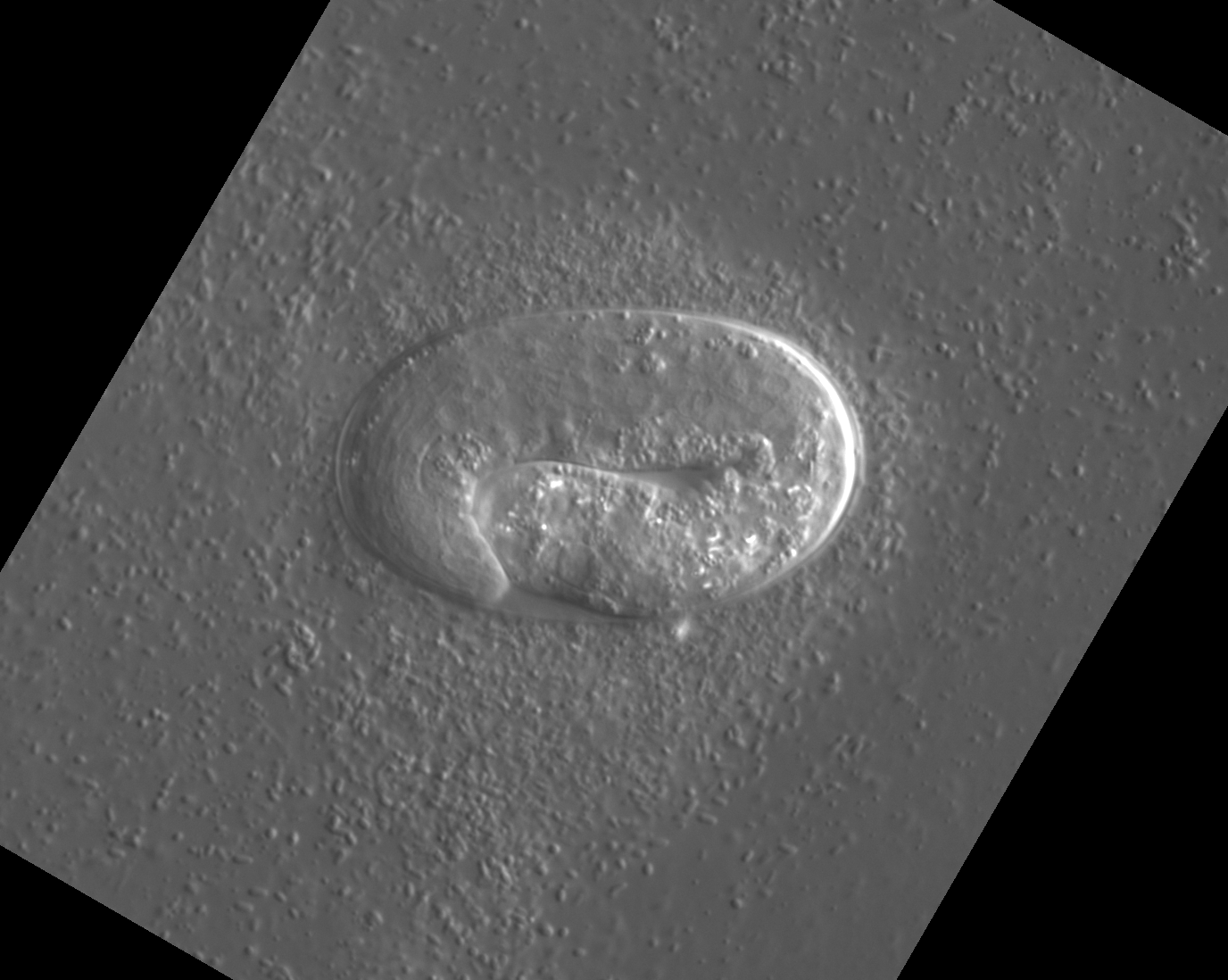

Supplement: Figure 2—source data 1. [file elife-85748-fig2-data1.zip › Figure2-Source_Data1/A-G/160221 RD367-65 embryo6 tardif.tif]

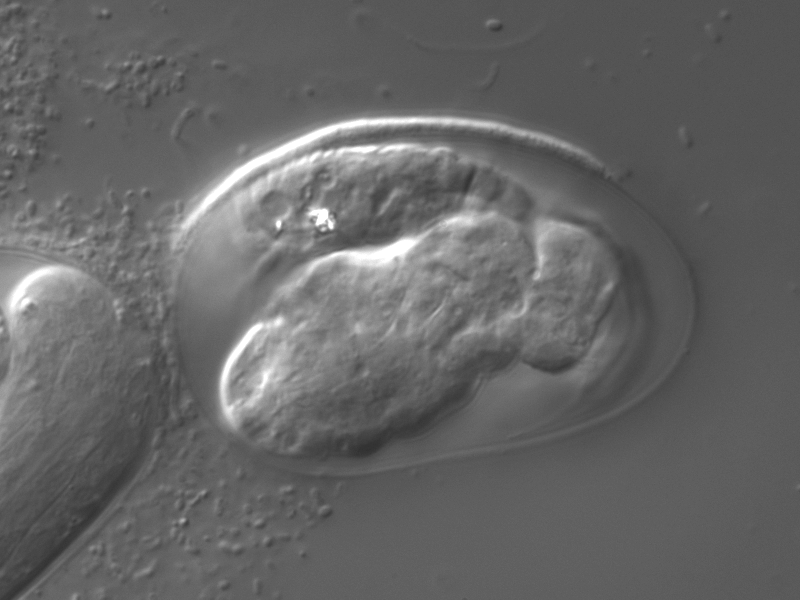

Supplement: Figure 2—source data 1. [file elife-85748-fig2-data1.zip › Figure2-Source_Data1/A-G/170222 RD363-22 embryo10- tardif choix 1.tif]

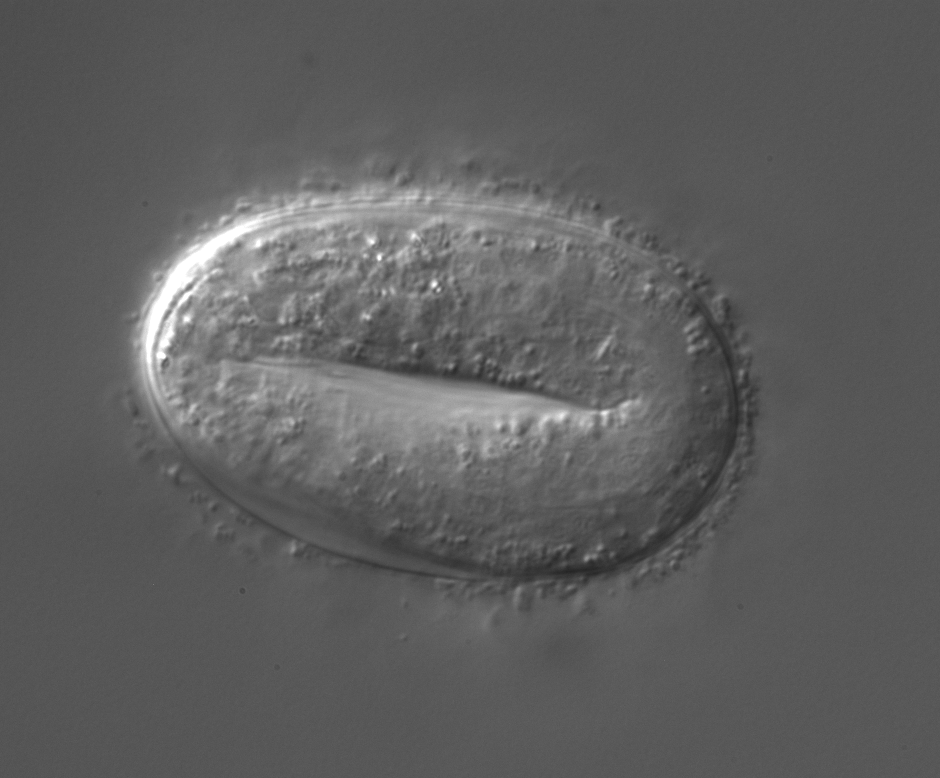

Supplement: Figure 2—source data 1. [file elife-85748-fig2-data1.zip › Figure2-Source_Data1/A-G/20161221 N2_embryon tardif.tif]

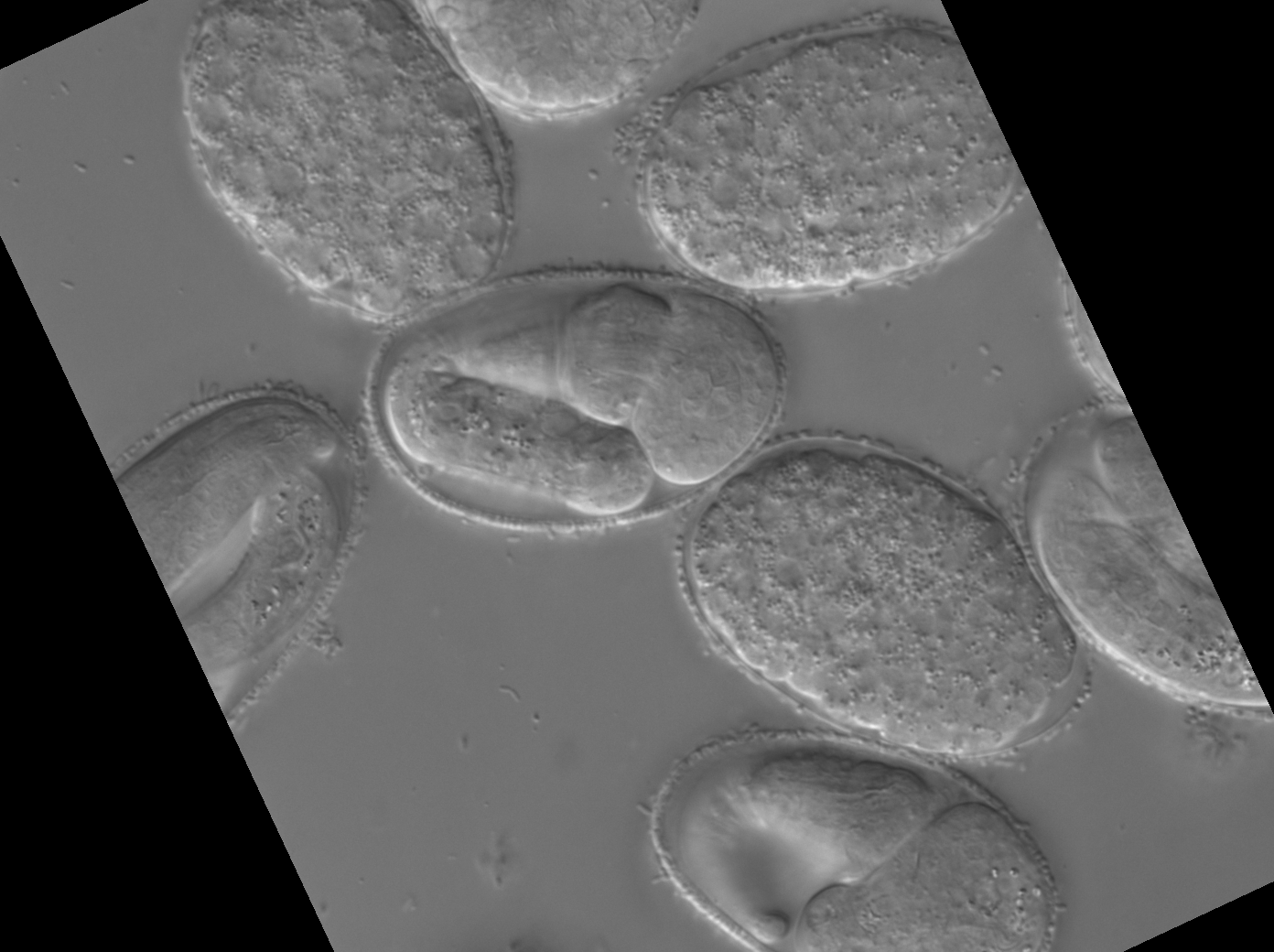

Supplement: Figure 2—source data 1. [file elife-85748-fig2-data1.zip › Figure2-Source_Data1/A-G/cl66-63X-tardif.tif]

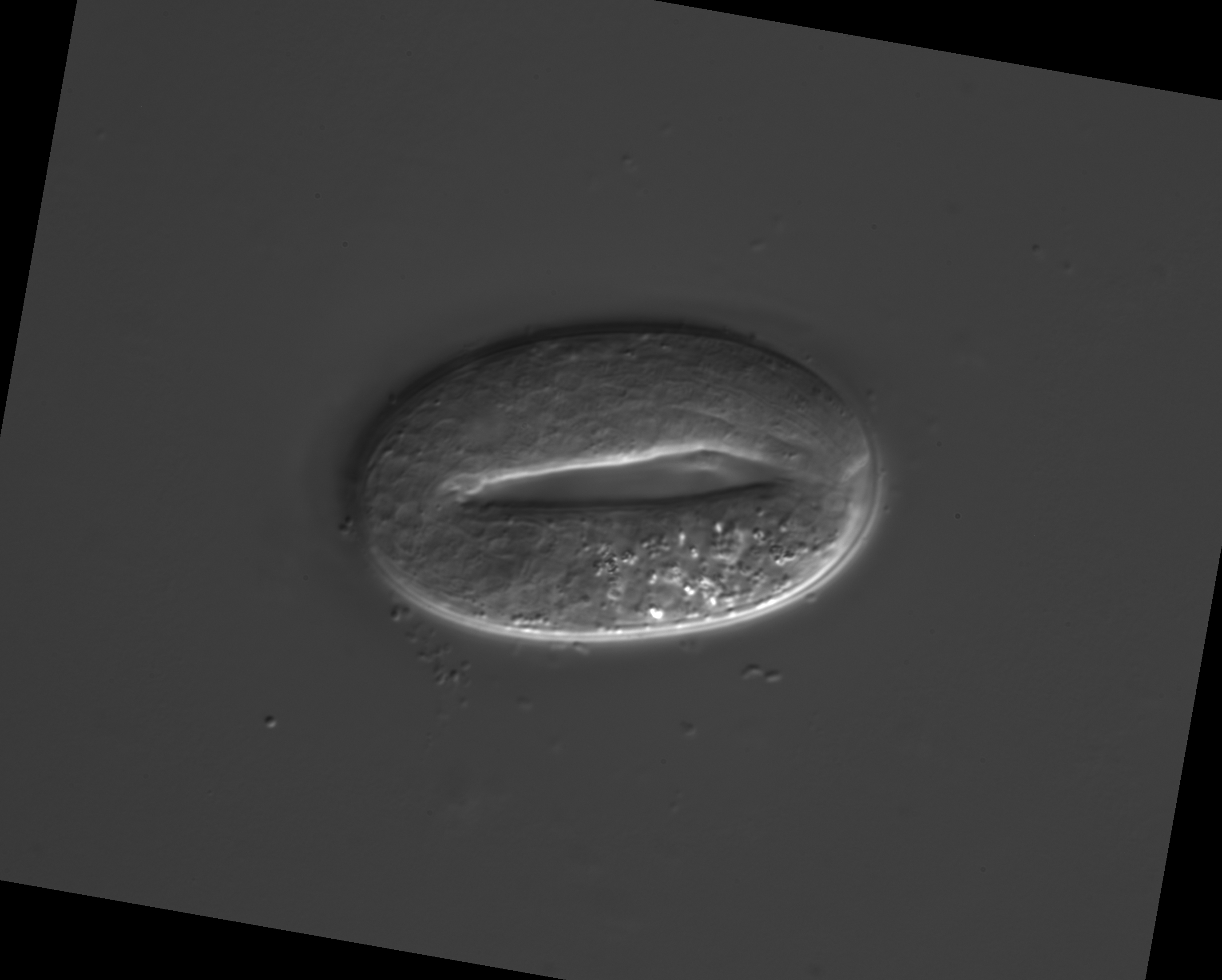

Supplement: Figure 2—source data 1. [file elife-85748-fig2-data1.zip › Figure2-Source_Data1/A-G/Snap-21895- tardif choix 1.tif]

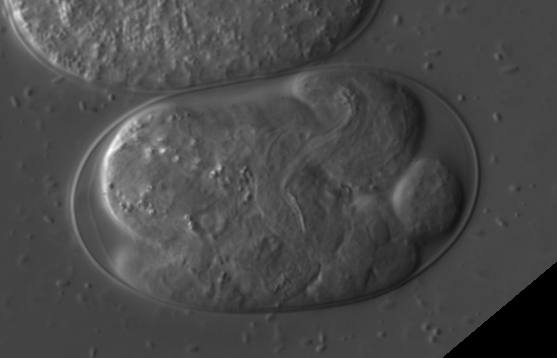

Supplement: Figure 2—source data 1. [file elife-85748-fig2-data1.zip › Figure2-Source_Data1/A-G/Snap-30383-1- tardif choix 1.tif]

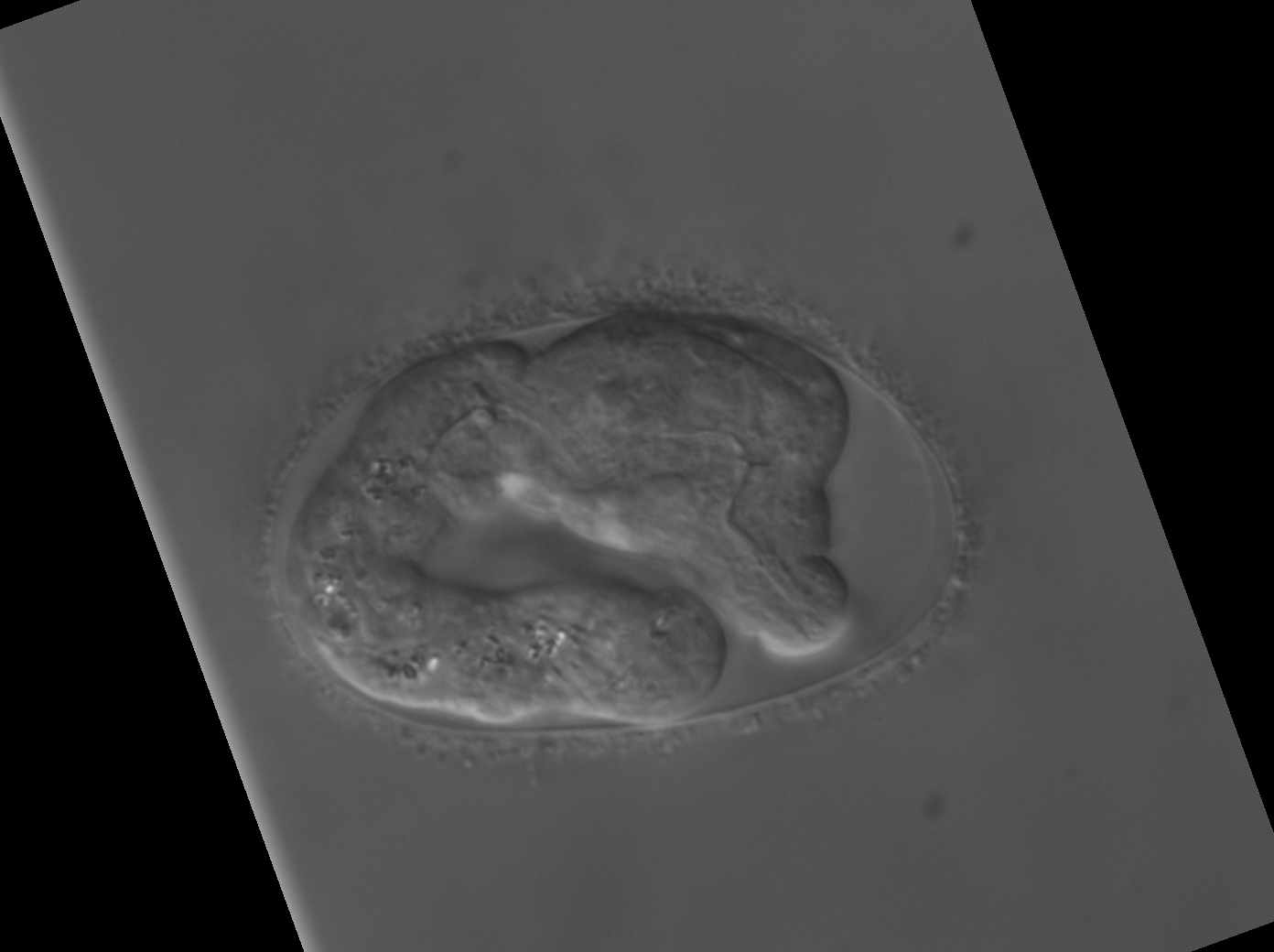

Supplement: Figure 2—source data 1. [file elife-85748-fig2-data1.zip › Figure2-Source_Data1/A-G/tm3489_100X-1 tardif.tif]

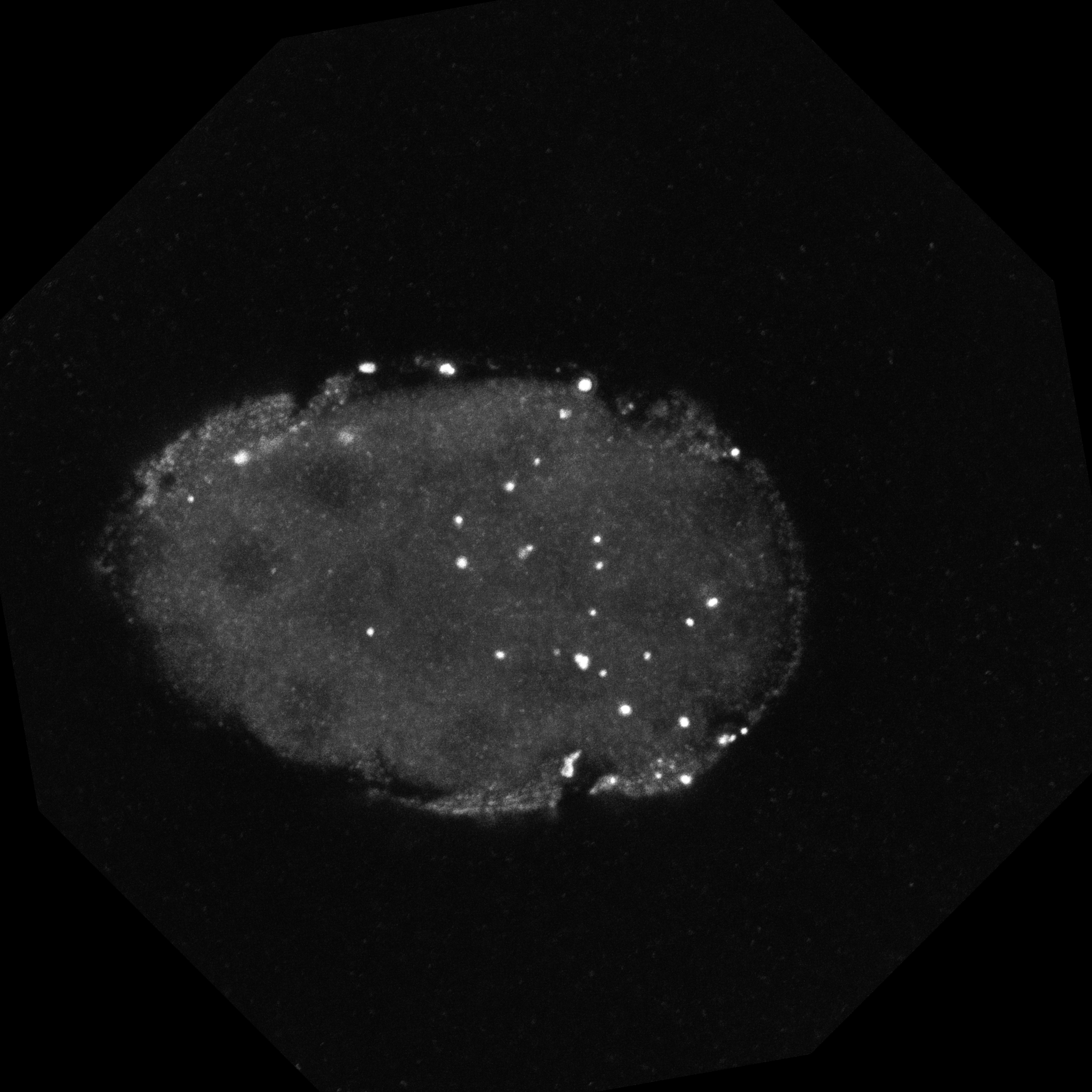

Supplement: Figure 3—source data 1. [file elife-85748-fig3-data1.zip › Figure3-Source_Data1/A-E/MAX_C1-20201007- GAstop spe11hsp6 gfp lgg-2.lif - Series028-1.tif]

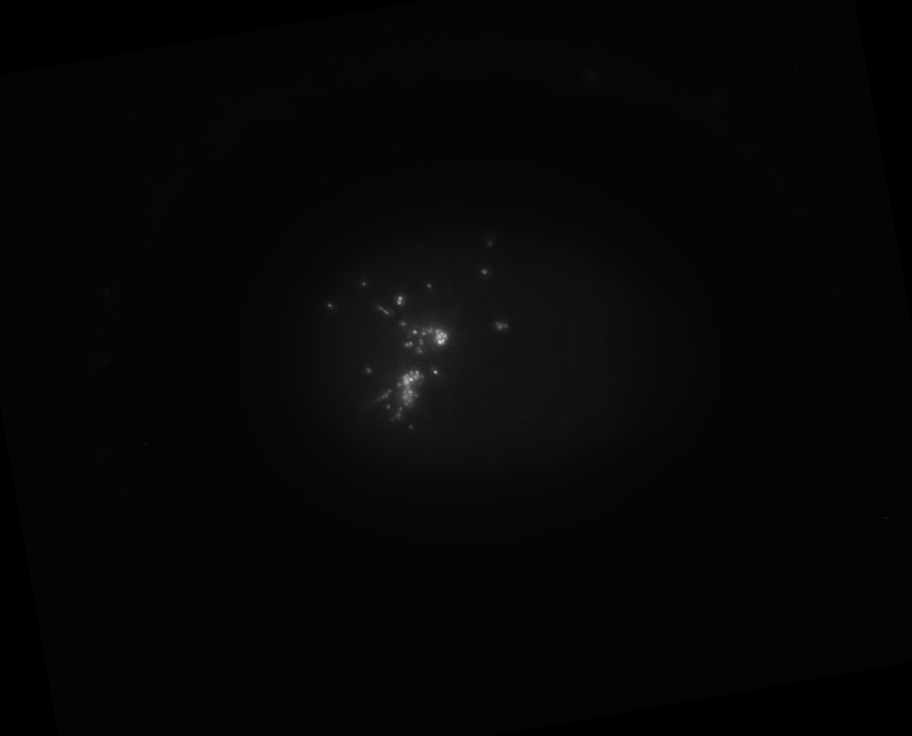

Supplement: Figure 3—source data 1. [file elife-85748-fig3-data1.zip › Figure3-Source_Data1/A-E/MAX_C1-Experiment-5890-1.tif]

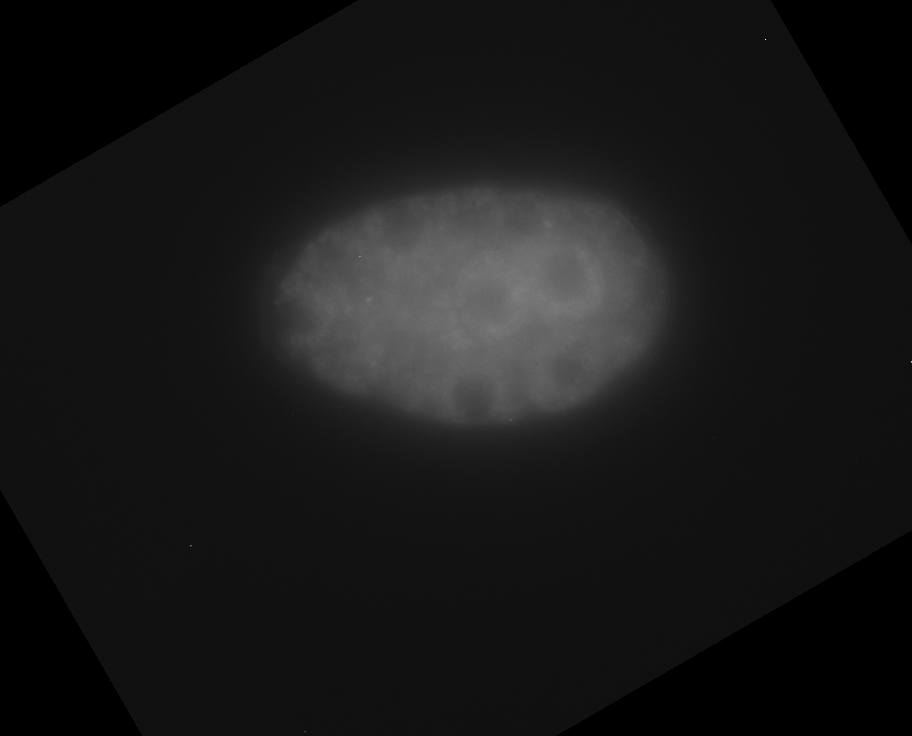

Supplement: Figure 3—source data 1. [file elife-85748-fig3-data1.zip › Figure3-Source_Data1/A-E/MAX_C1-Experiment-5952-15C.tif]

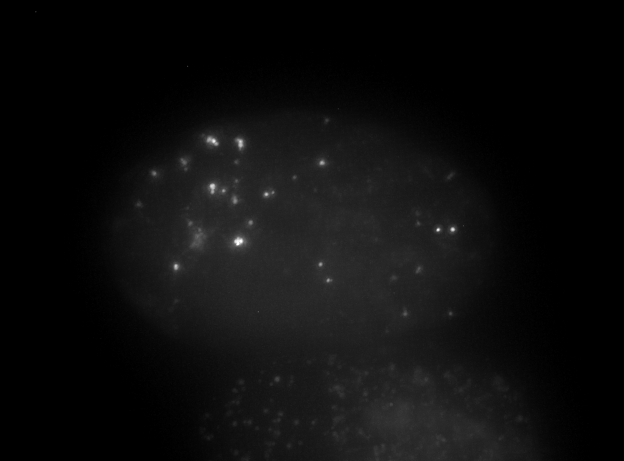

Supplement: Figure 3—source data 1. [file elife-85748-fig3-data1.zip › Figure3-Source_Data1/A-E/MAX_C1-Experiment-7394-1 crop2.tif]

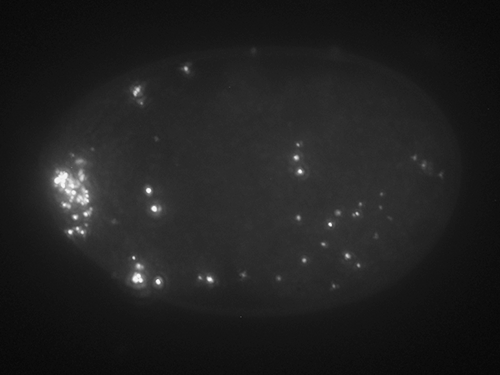

Supplement: Figure 3—source data 1. [file elife-85748-fig3-data1.zip › Figure3-Source_Data1/A-E/MAX_C1-Experiment-8645-recad-redim.tif]

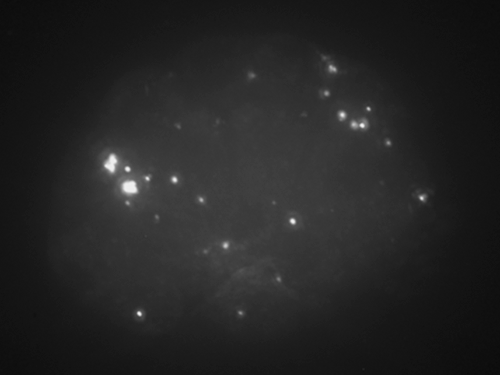

Supplement: Figure 3—source data 1. [file elife-85748-fig3-data1.zip › Figure3-Source_Data1/A-E/MAX_C1-Experiment-8658-redim-recad.tif]

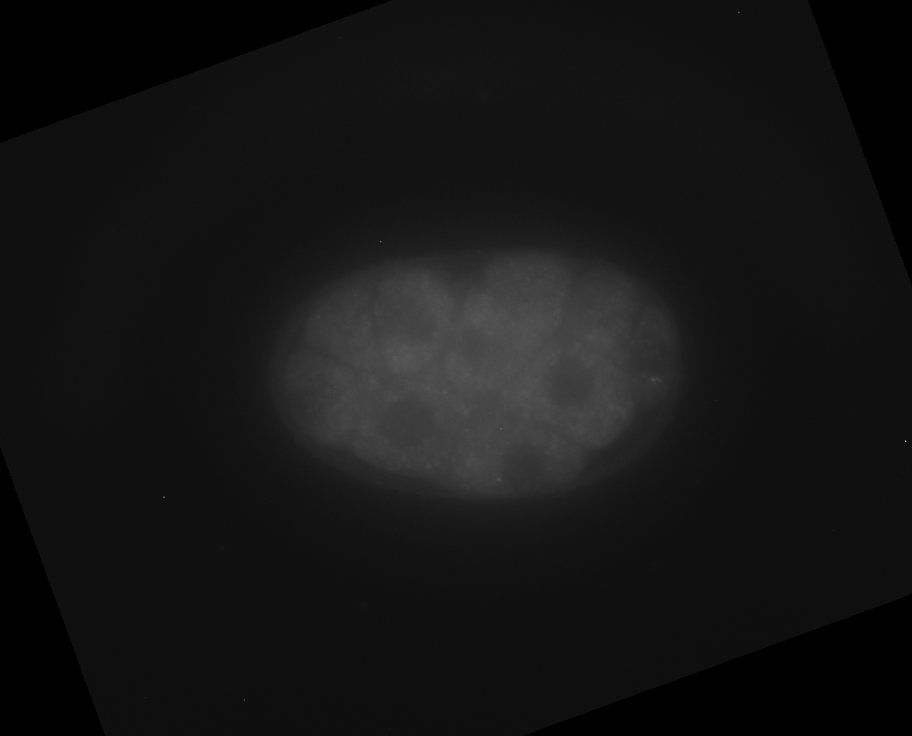

Supplement: Figure 3—source data 1. [file elife-85748-fig3-data1.zip › Figure3-Source_Data1/A-E/MAX_Experiment-5900-.tif]

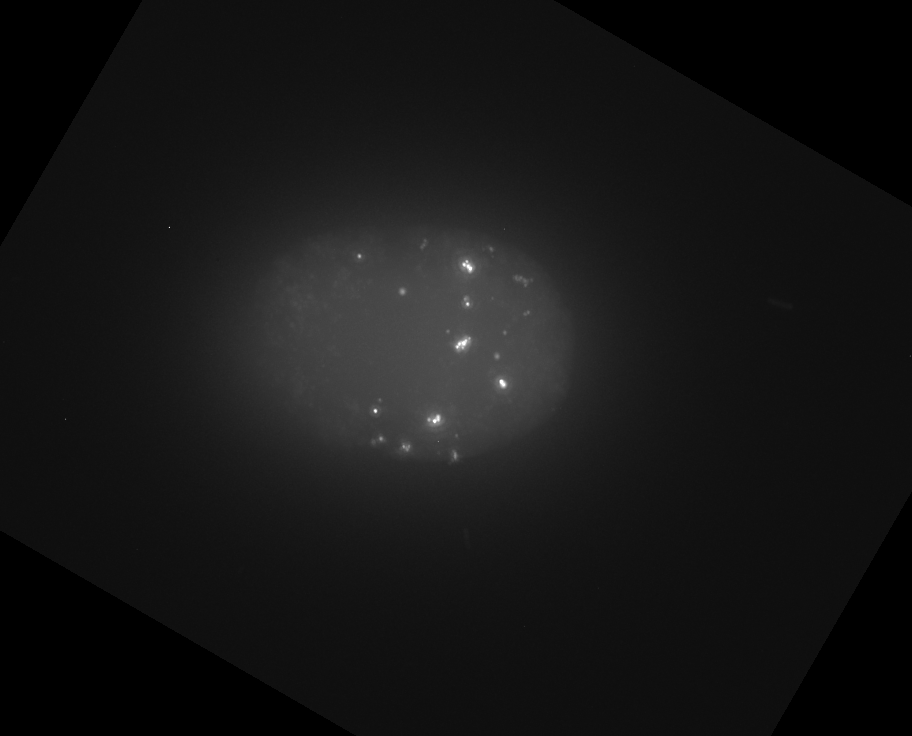

Supplement: Figure 3—source data 1. [file elife-85748-fig3-data1.zip › Figure3-Source_Data1/A-E/MAX_Experiment-5918- 1C pp65-0-4095-8bit.tif]

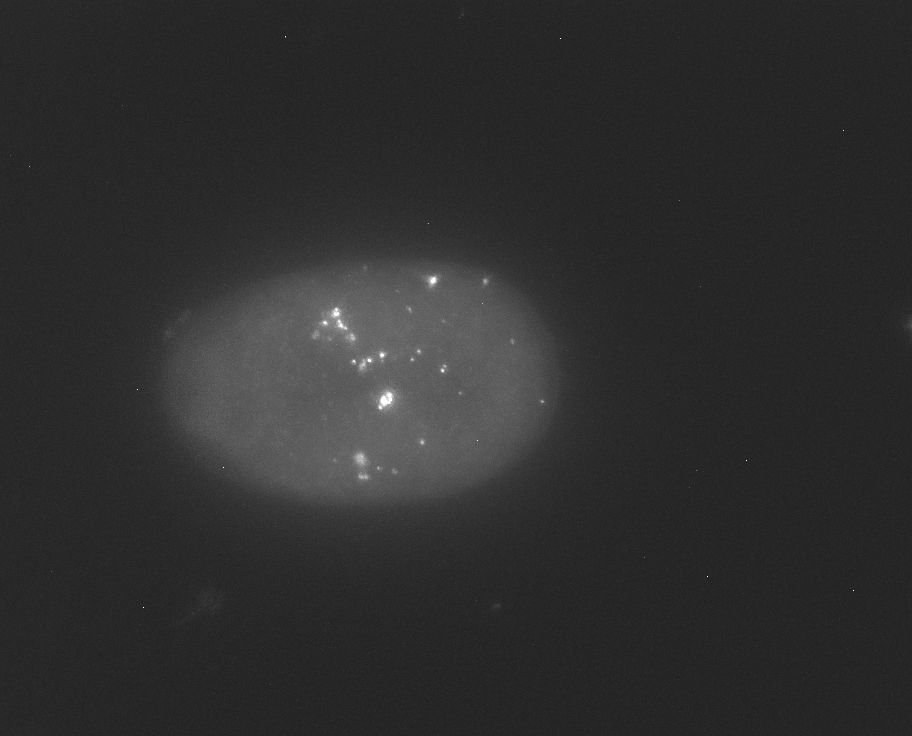

Supplement: Figure 3—source data 1. [file elife-85748-fig3-data1.zip › Figure3-Source_Data1/A-E/MAX_Experiment-7267- 8 bits.tif]

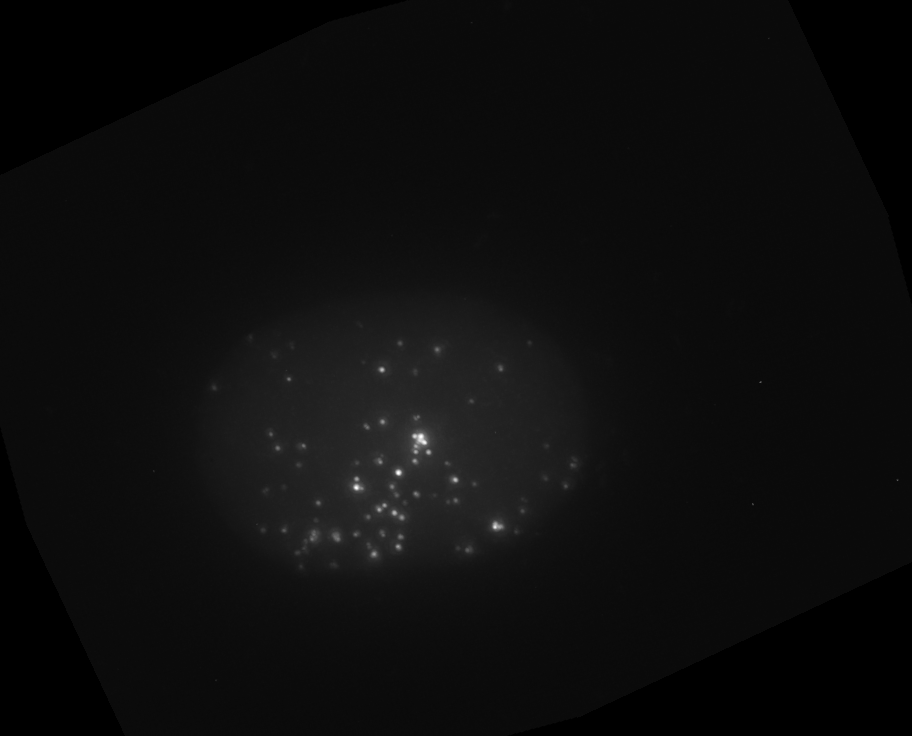

Supplement: Figure 3—source data 1. [file elife-85748-fig3-data1.zip › Figure3-Source_Data1/A-E/MAX_Experiment-7395-1.tif]

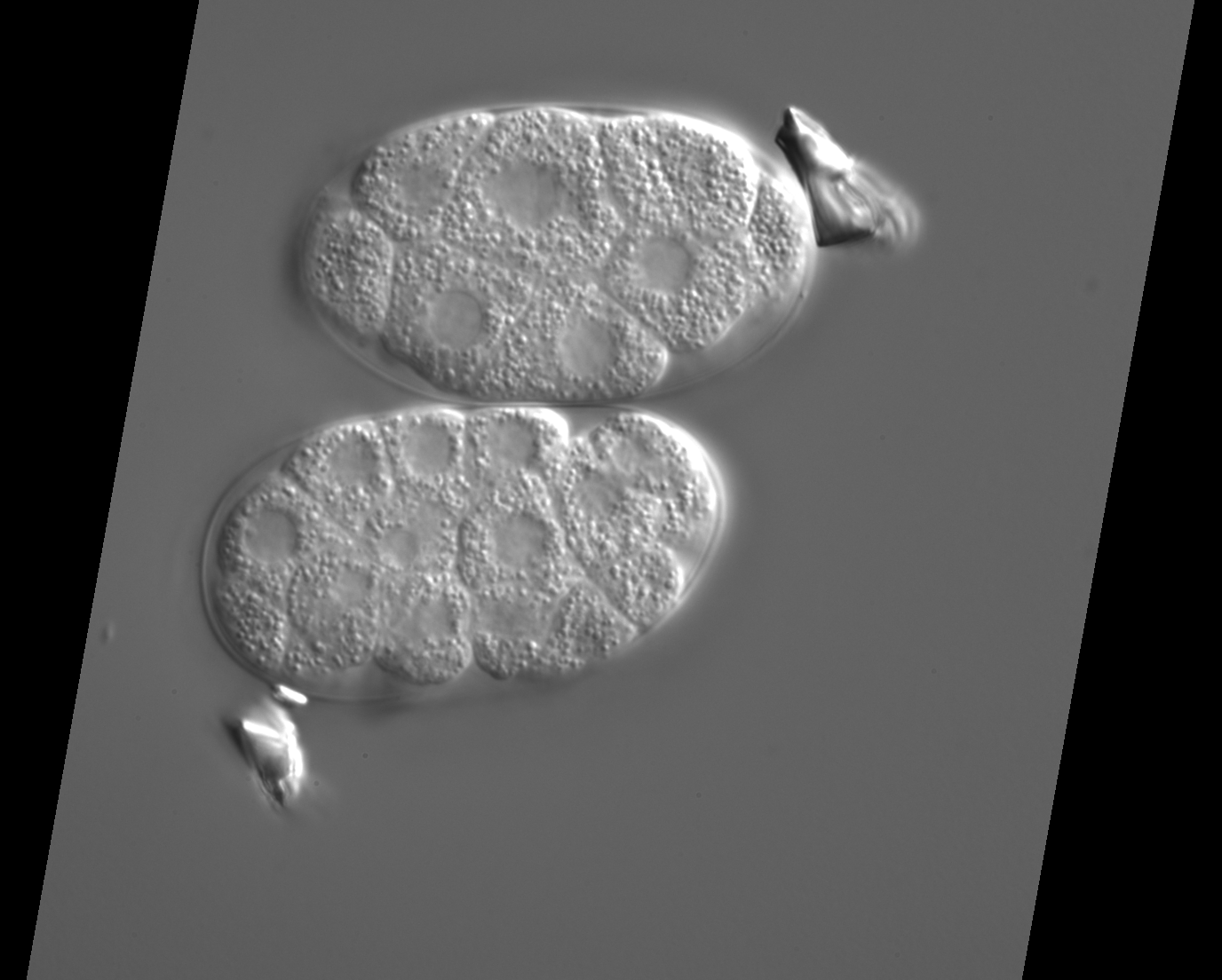

Supplement: Figure 3—figure supplement 1—source data 1. [file elife-85748-fig3-figsupp1-data1.zip › Figure3-figure-supplement1-Source_Data1/A-B/DIC- 15CExperiment-7169-1.tif]

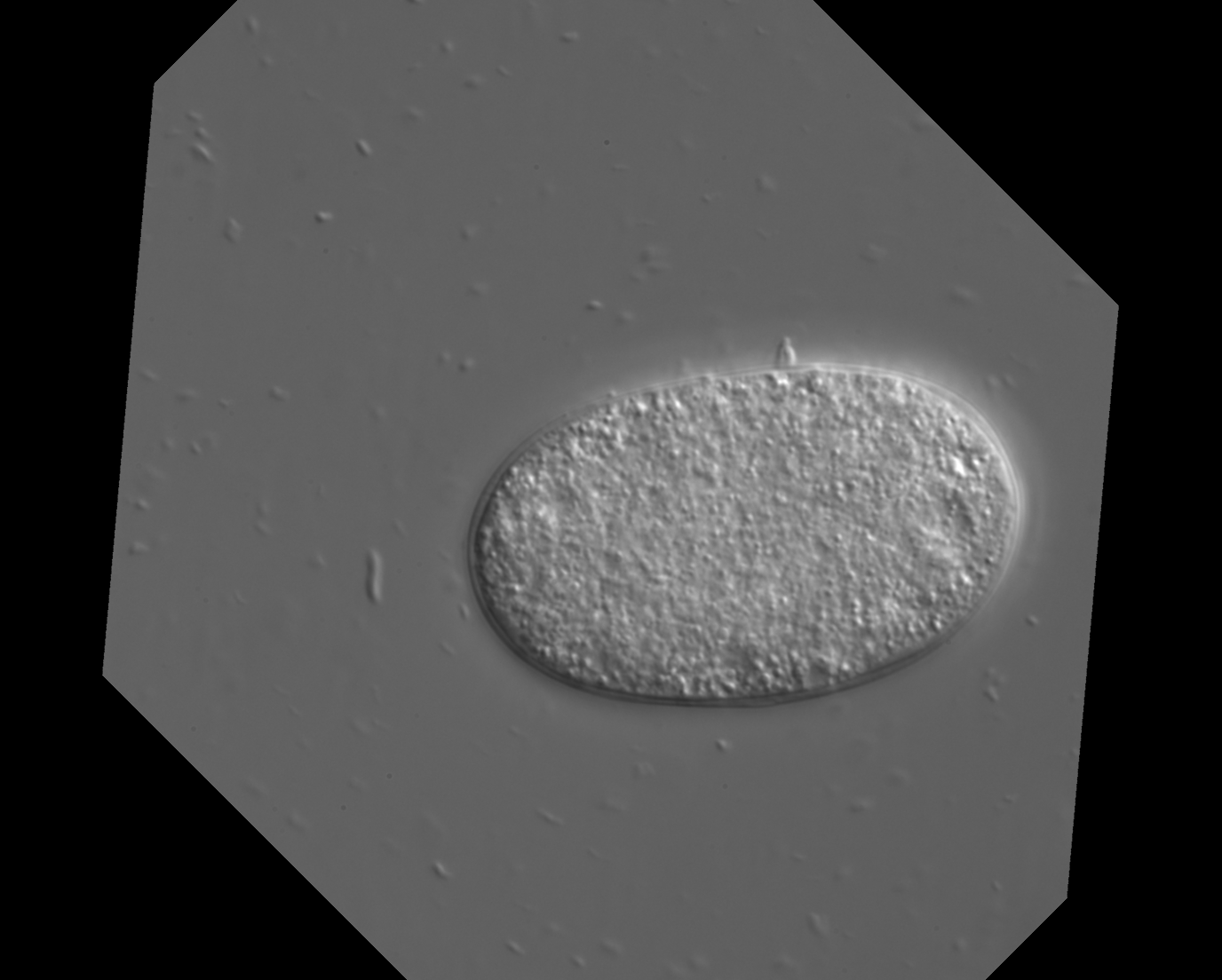

Supplement: Figure 3—figure supplement 1—source data 1. [file elife-85748-fig3-figsupp1-data1.zip › Figure3-figure-supplement1-Source_Data1/A-B/DIC C1-Experiment-7220.tif]

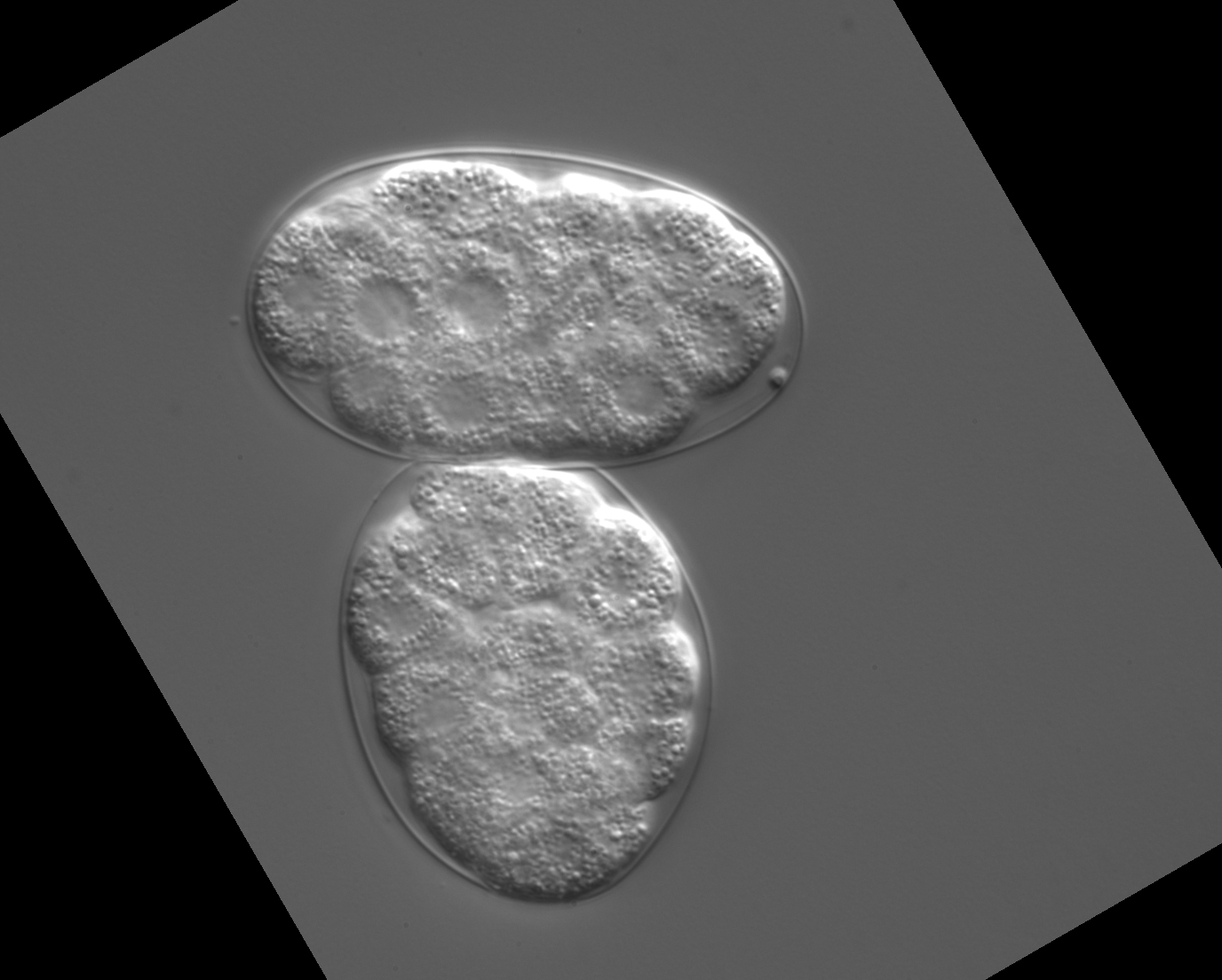

Supplement: Figure 3—figure supplement 1—source data 1. [file elife-85748-fig3-figsupp1-data1.zip › Figure3-figure-supplement1-Source_Data1/A-B/DIC_15CC1-Experiment-7252.tif]

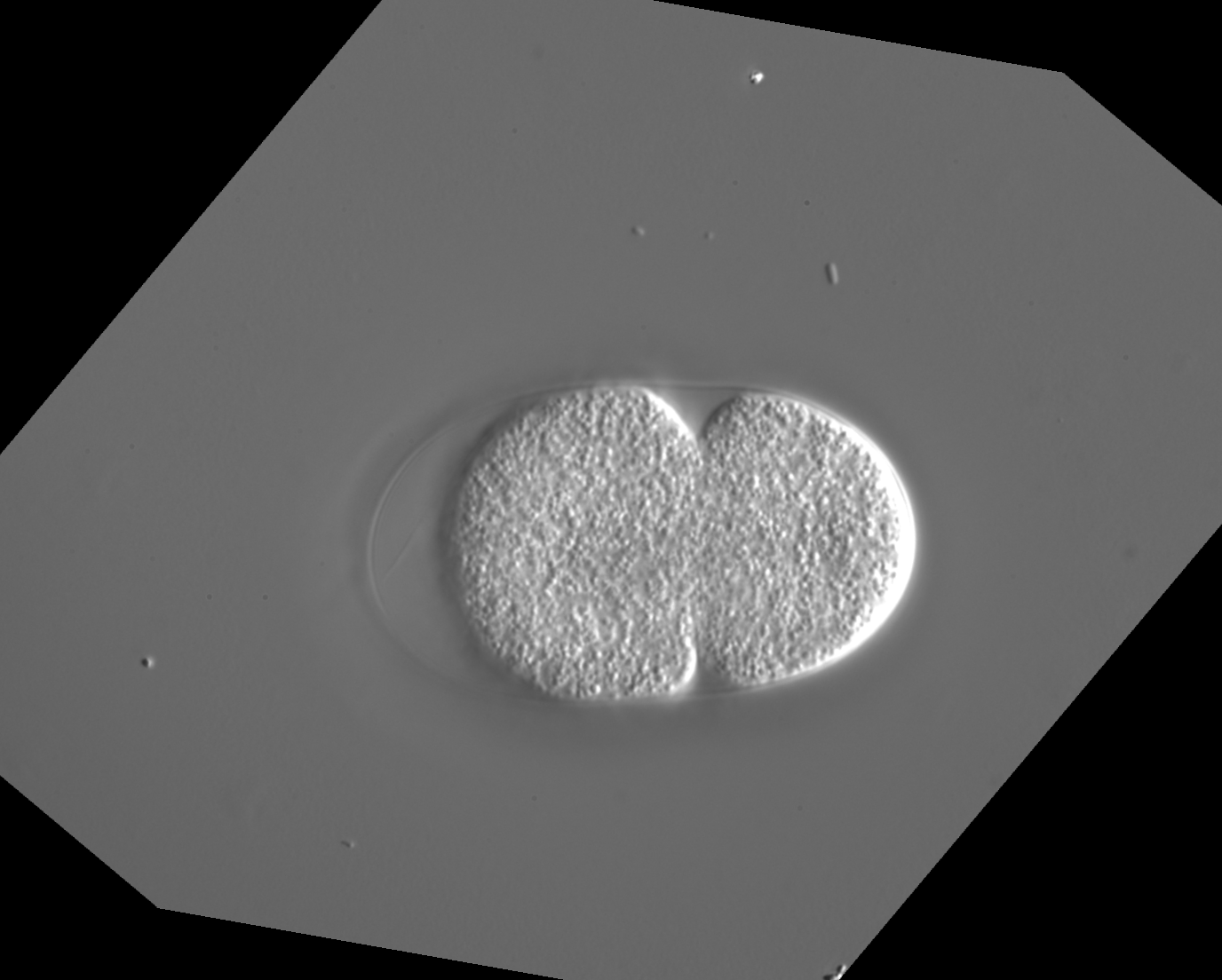

Supplement: Figure 3—figure supplement 1—source data 1. [file elife-85748-fig3-figsupp1-data1.zip › Figure3-figure-supplement1-Source_Data1/A-B/DIC_C1-Experiment-7129.tif]

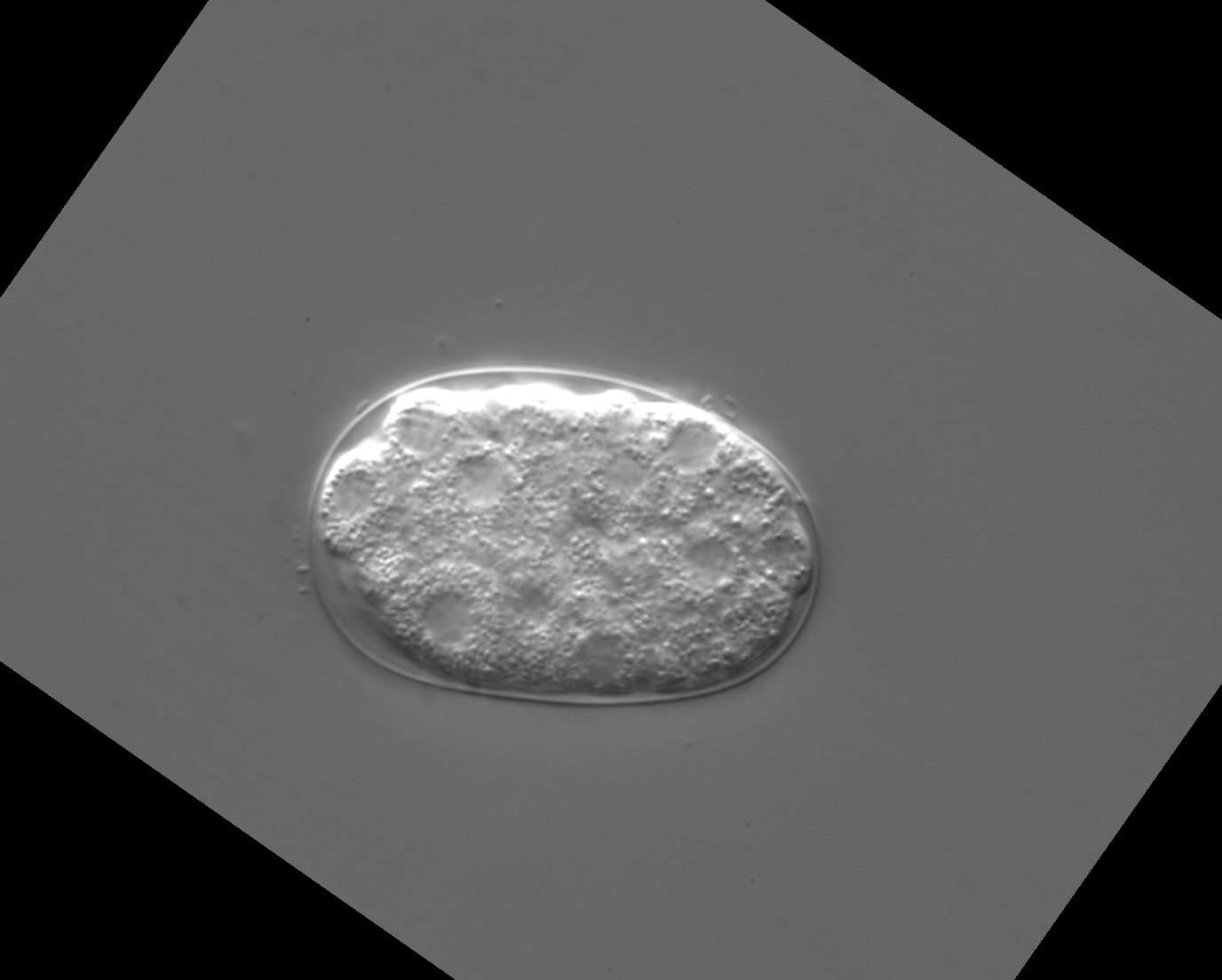

Supplement: Figure 3—figure supplement 1—source data 1. [file elife-85748-fig3-figsupp1-data1.zip › Figure3-figure-supplement1-Source_Data1/A-B/DIC_C1-Experiment-7136.tif]

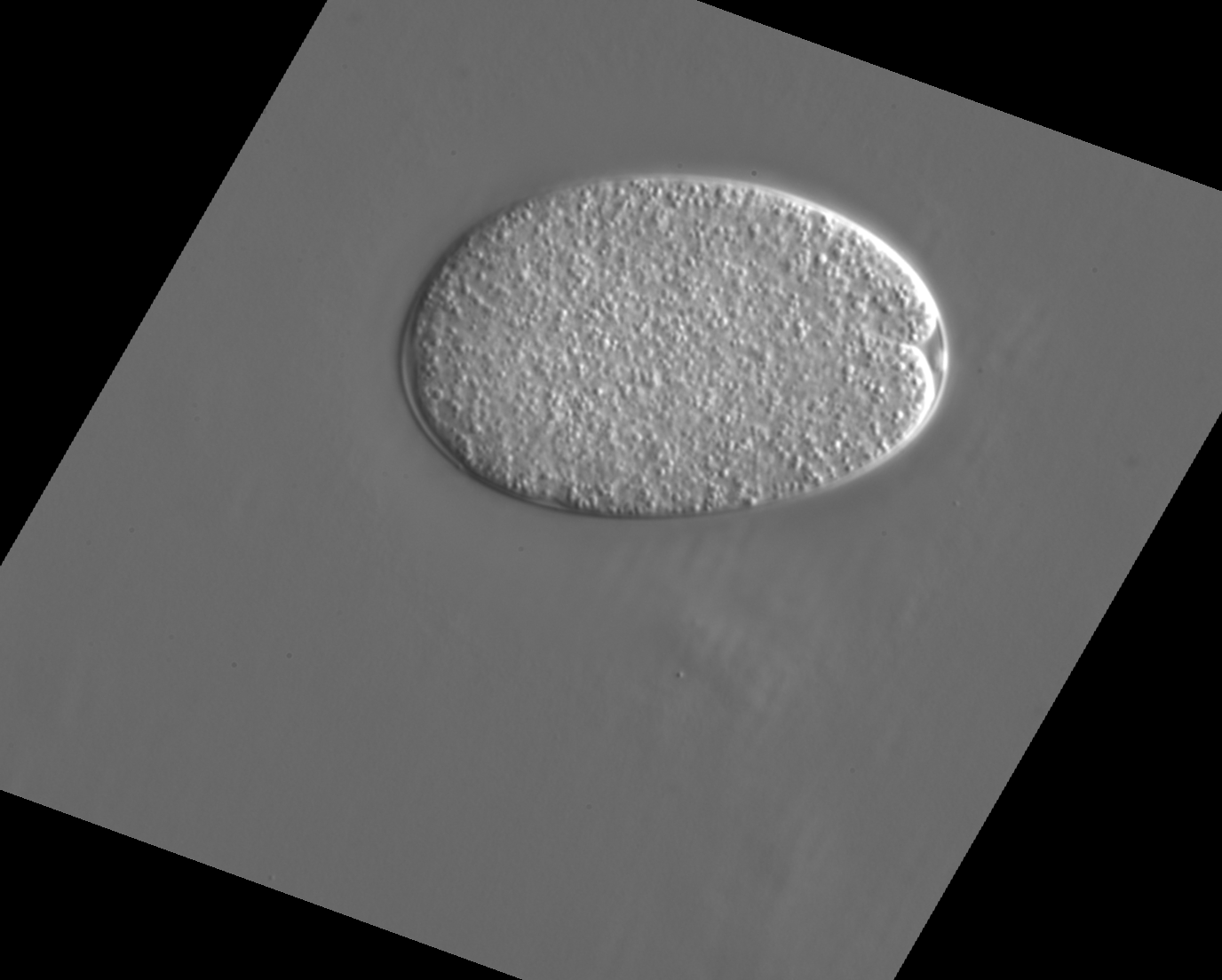

Supplement: Figure 3—figure supplement 1—source data 1. [file elife-85748-fig3-figsupp1-data1.zip › Figure3-figure-supplement1-Source_Data1/A-B/DIC_C1-Experiment-7153.tif]

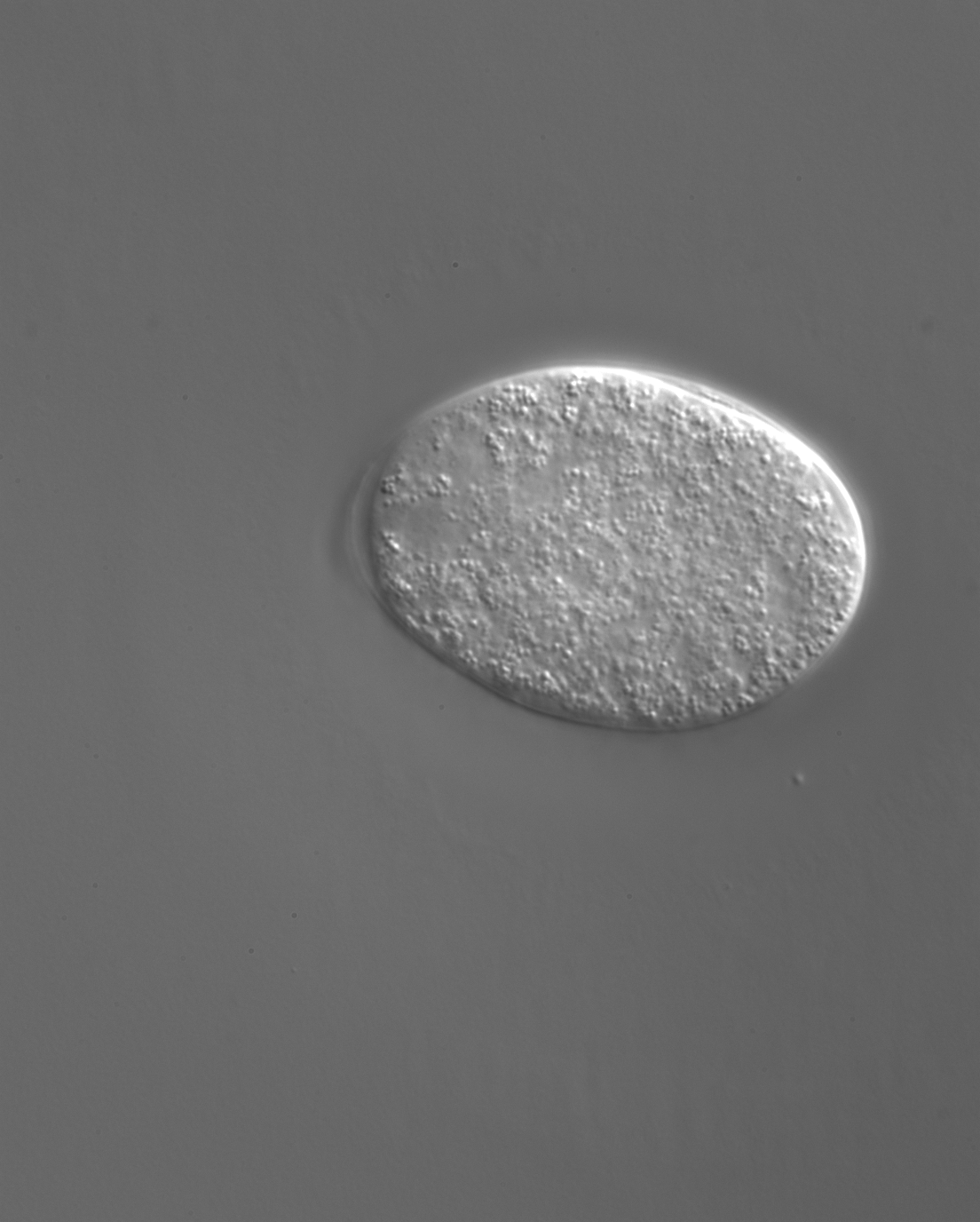

Supplement: Figure 3—figure supplement 1—source data 1. [file elife-85748-fig3-figsupp1-data1.zip › Figure3-figure-supplement1-Source_Data1/A-B/DIC_C1-Experiment-7156.tif]

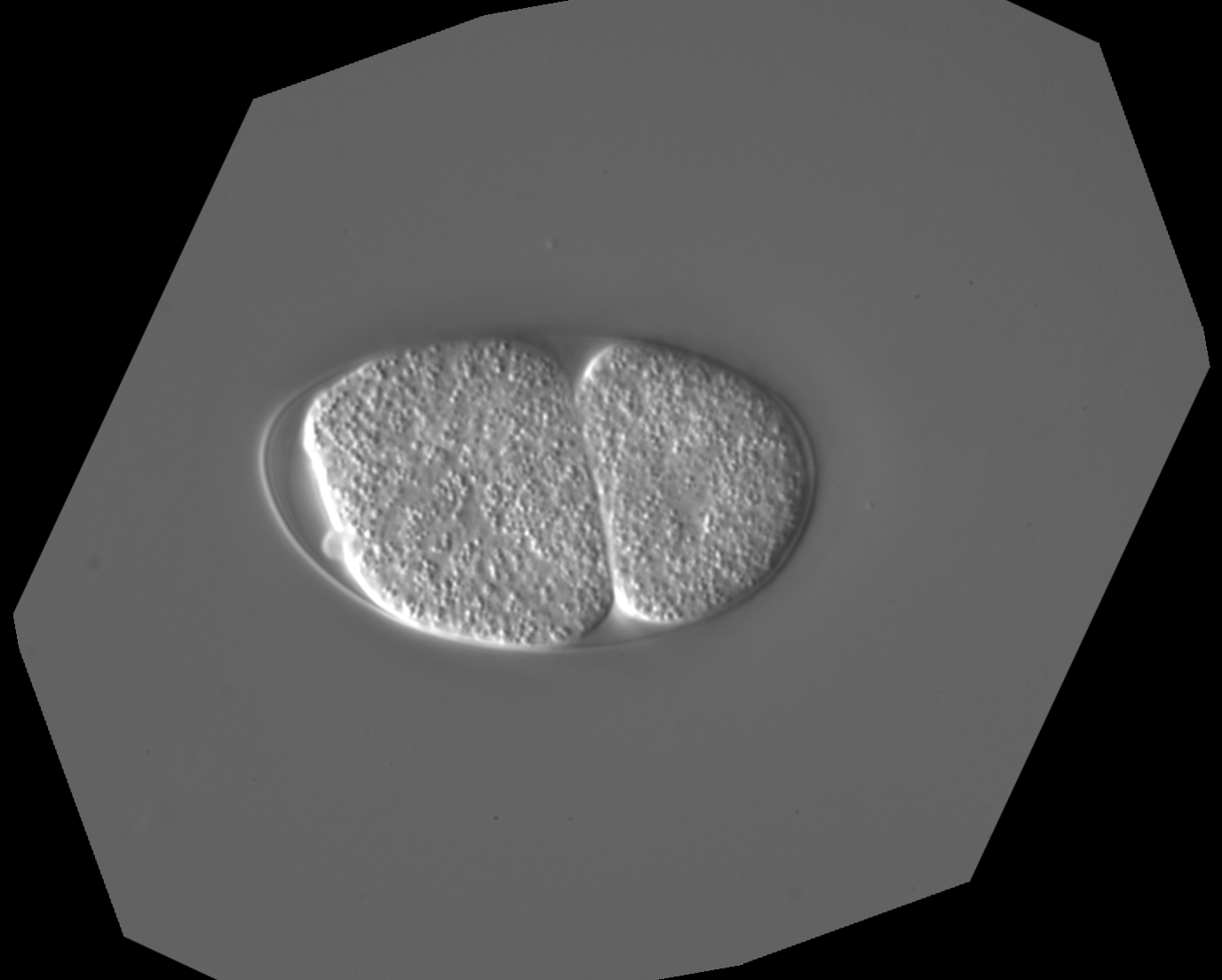

Supplement: Figure 3—figure supplement 1—source data 1. [file elife-85748-fig3-figsupp1-data1.zip › Figure3-figure-supplement1-Source_Data1/A-B/DIC_C1-Experiment-7164-1.tif]

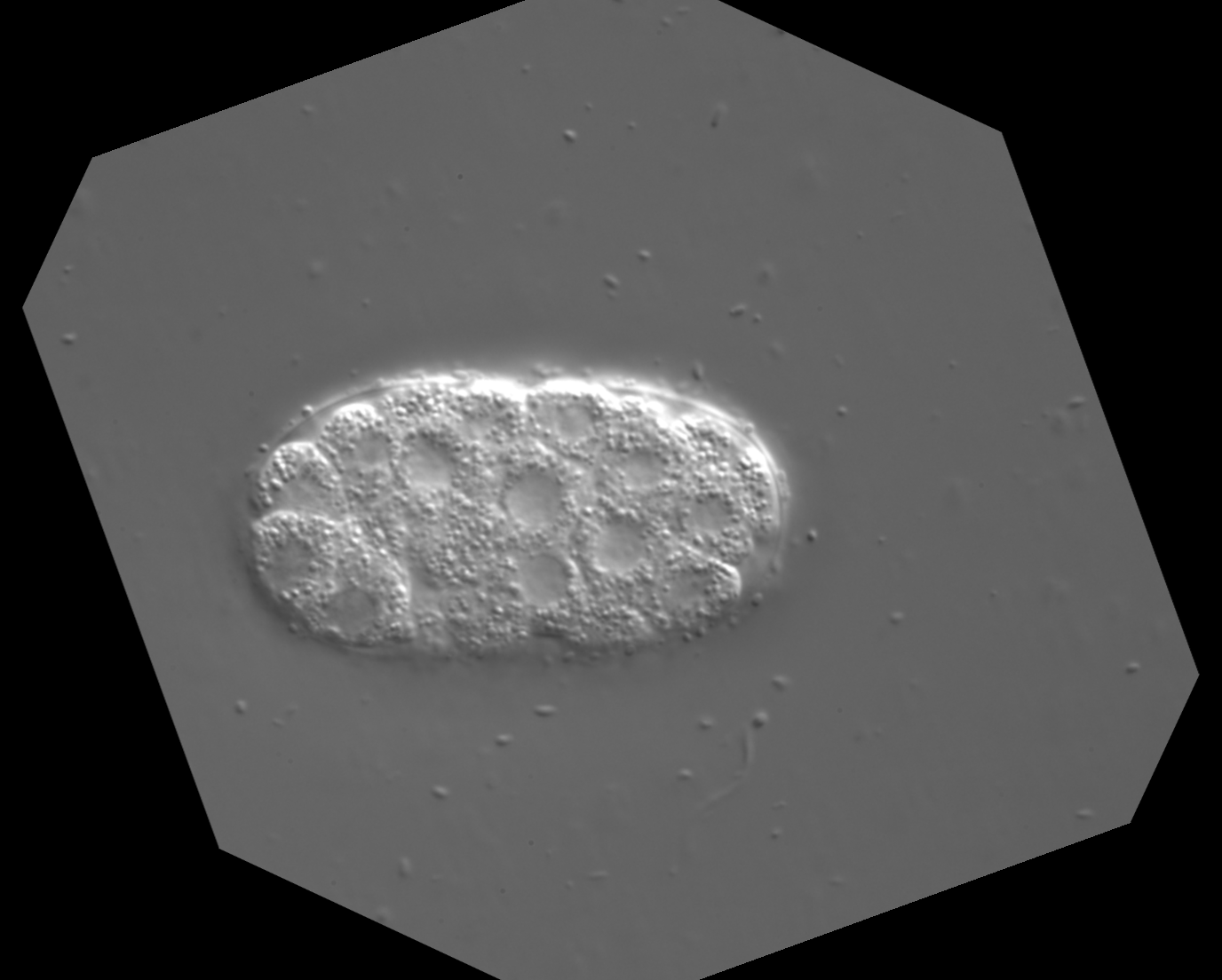

Supplement: Figure 3—figure supplement 1—source data 1. [file elife-85748-fig3-figsupp1-data1.zip › Figure3-figure-supplement1-Source_Data1/A-B/DIC-15 C1-Experiment-7223.tif]

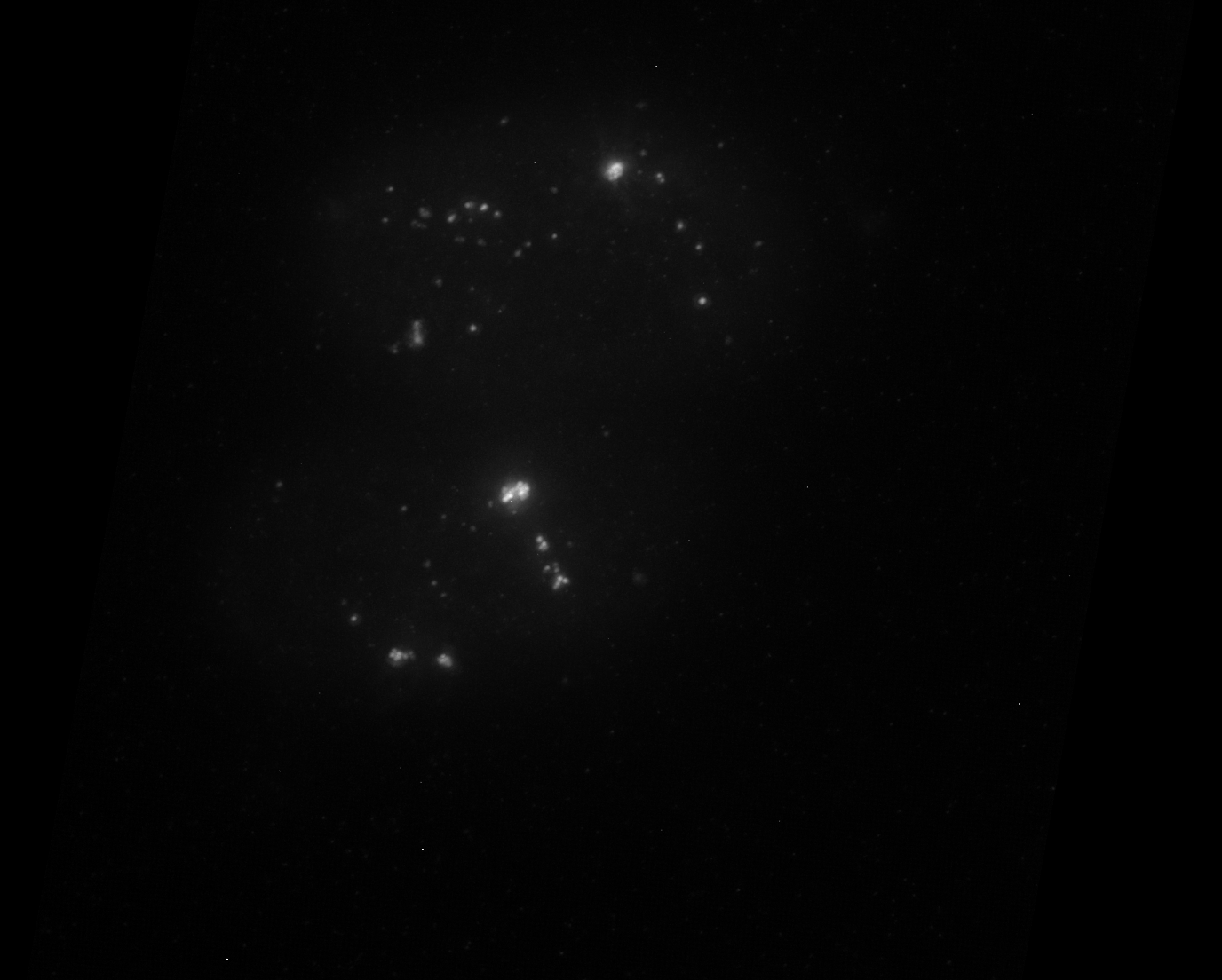

Supplement: Figure 3—figure supplement 1—source data 1. [file elife-85748-fig3-figsupp1-data1.zip › Figure3-figure-supplement1-Source_Data1/A-B/MAX_15CC1-Experiment-7169.tif]

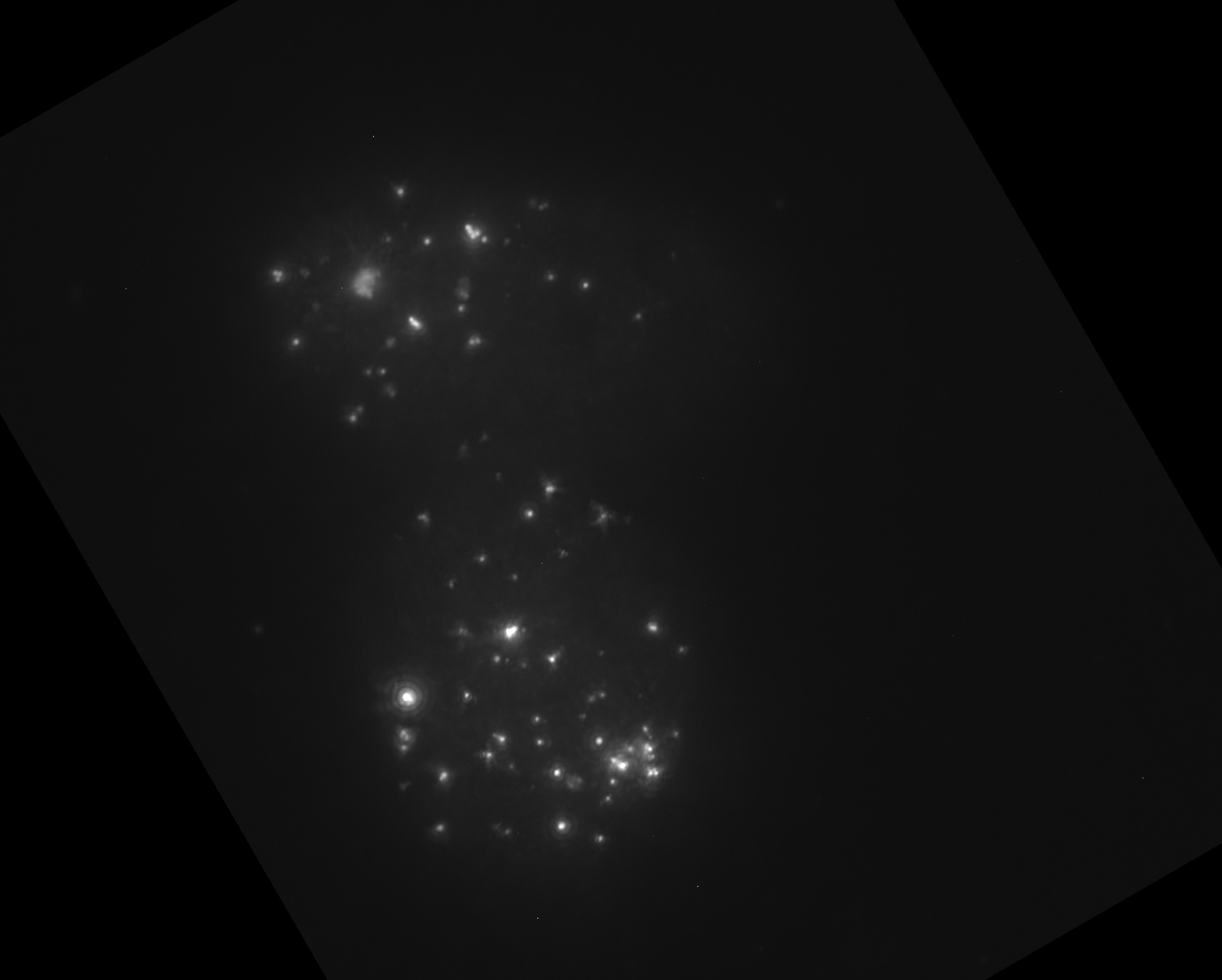

Supplement: Figure 3—figure supplement 1—source data 1. [file elife-85748-fig3-figsupp1-data1.zip › Figure3-figure-supplement1-Source_Data1/A-B/MAX_15CC1-Experiment-7252.tif]

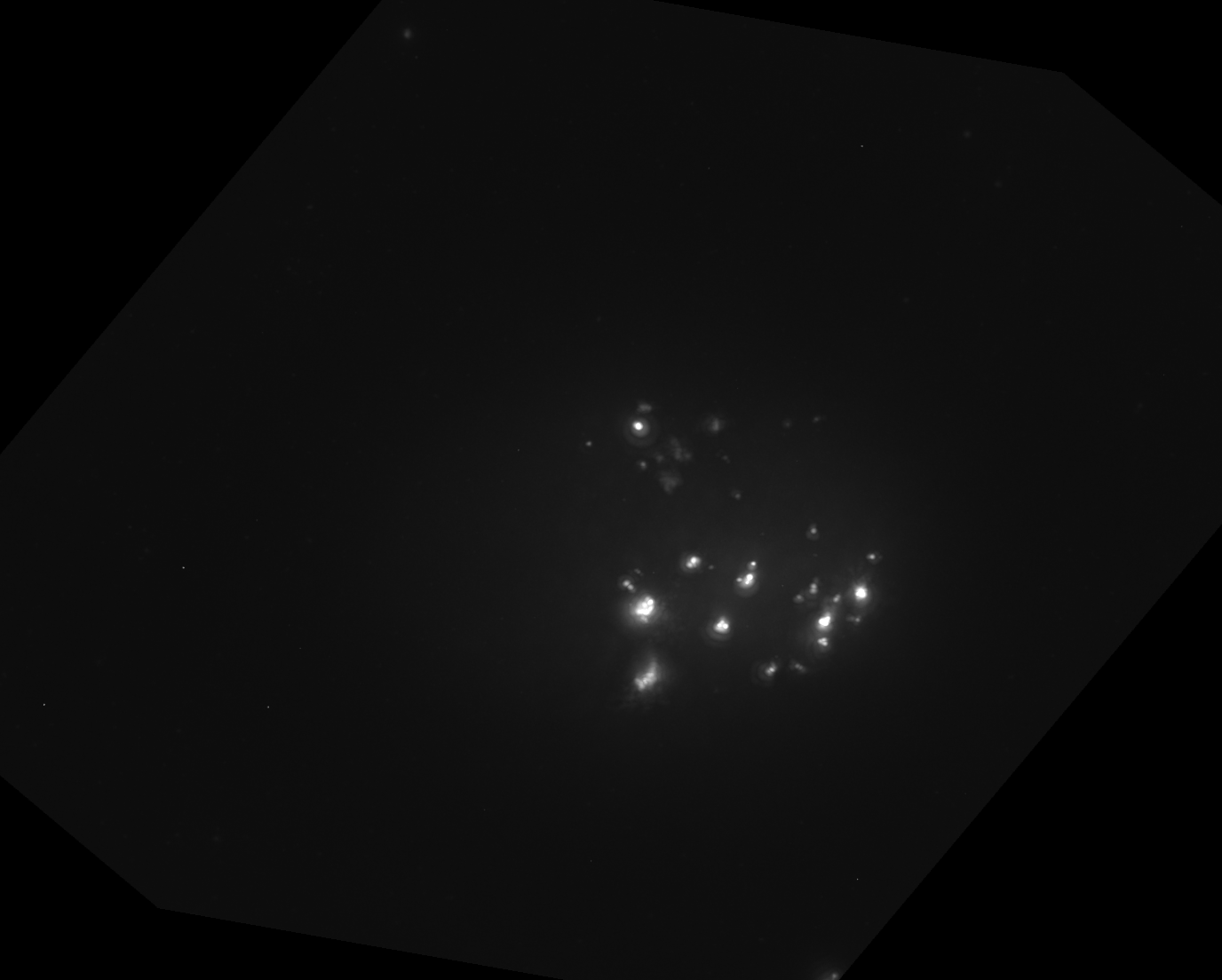

Supplement: Figure 3—figure supplement 1—source data 1. [file elife-85748-fig3-figsupp1-data1.zip › Figure3-figure-supplement1-Source_Data1/A-B/MAX_C1-Experiment-7129.tif]

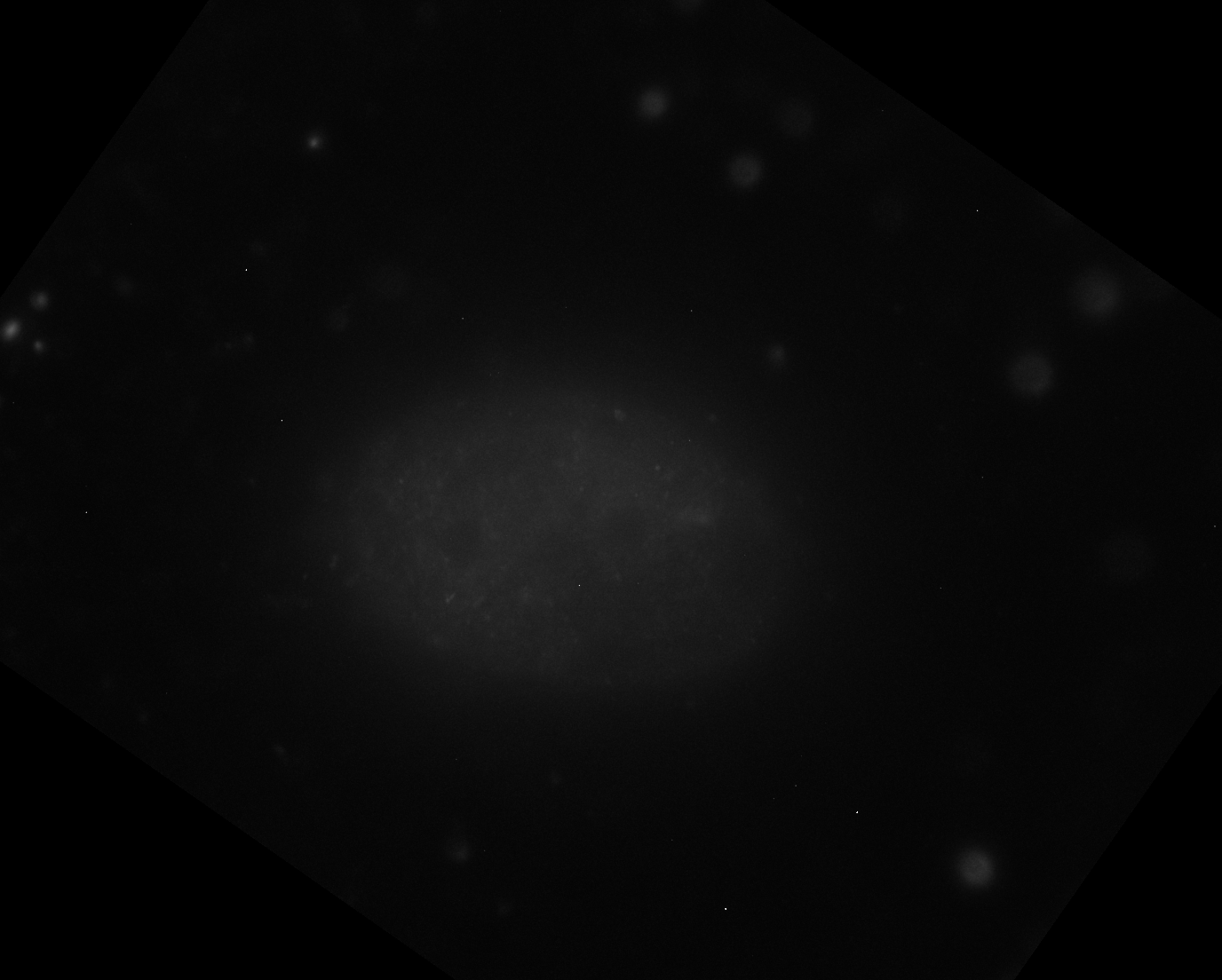

Supplement: Figure 3—figure supplement 1—source data 1. [file elife-85748-fig3-figsupp1-data1.zip › Figure3-figure-supplement1-Source_Data1/A-B/MAX_C1-Experiment-7136.tif]

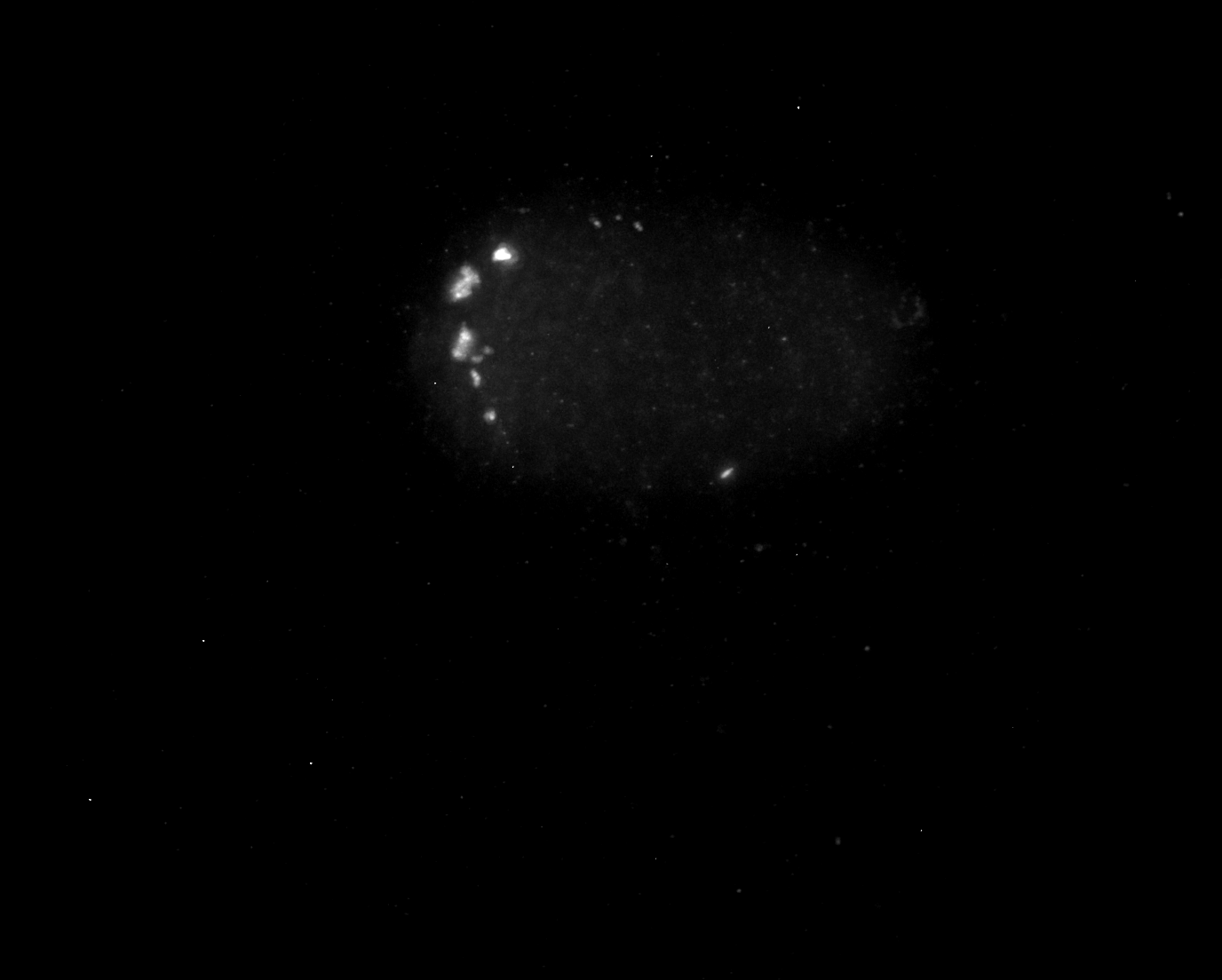

Supplement: Figure 3—figure supplement 1—source data 1. [file elife-85748-fig3-figsupp1-data1.zip › Figure3-figure-supplement1-Source_Data1/A-B/MAX_C1-Experiment-7153.tif]

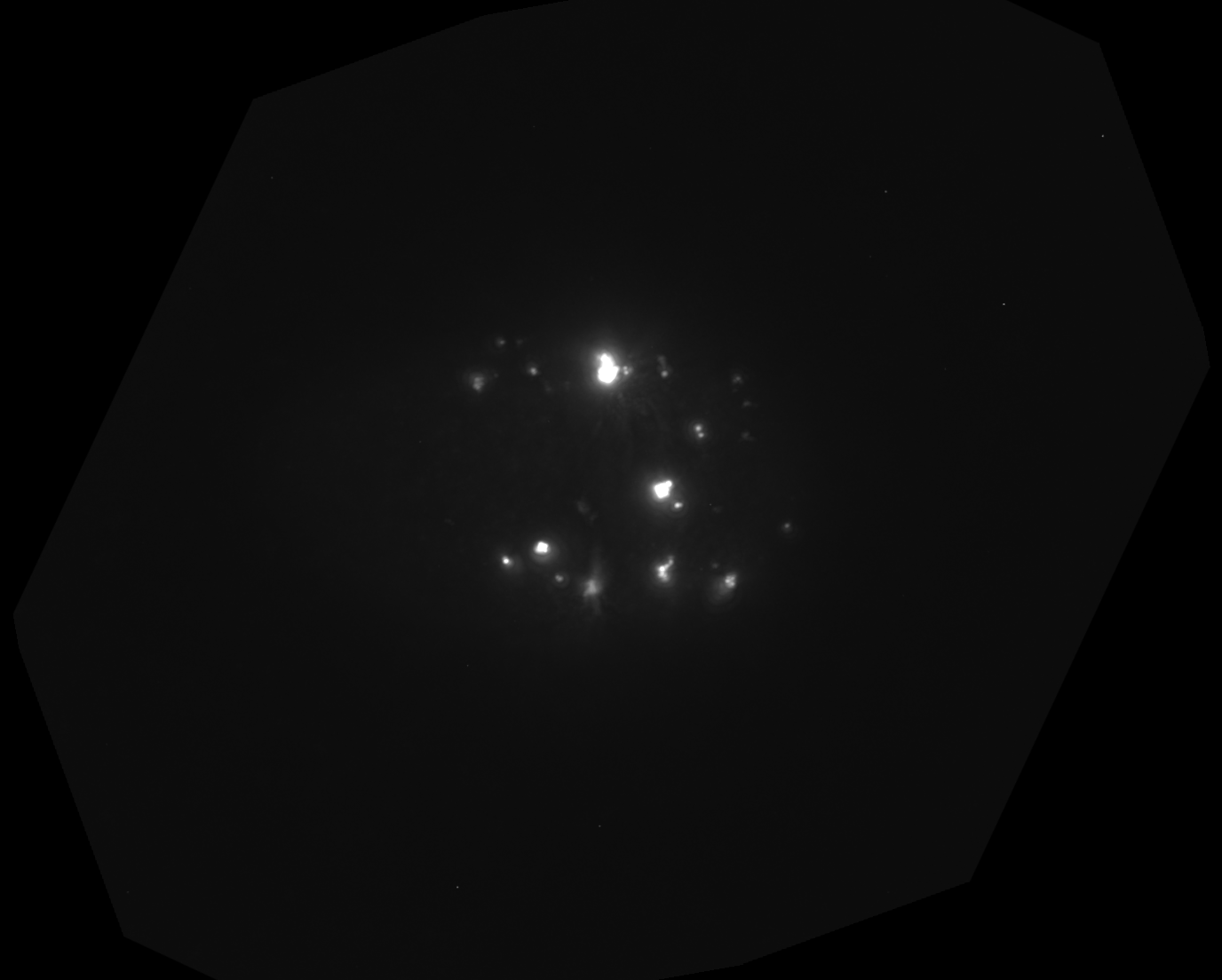

Supplement: Figure 3—figure supplement 1—source data 1. [file elife-85748-fig3-figsupp1-data1.zip › Figure3-figure-supplement1-Source_Data1/A-B/MAX_C1-Experiment-7164-1.tif]

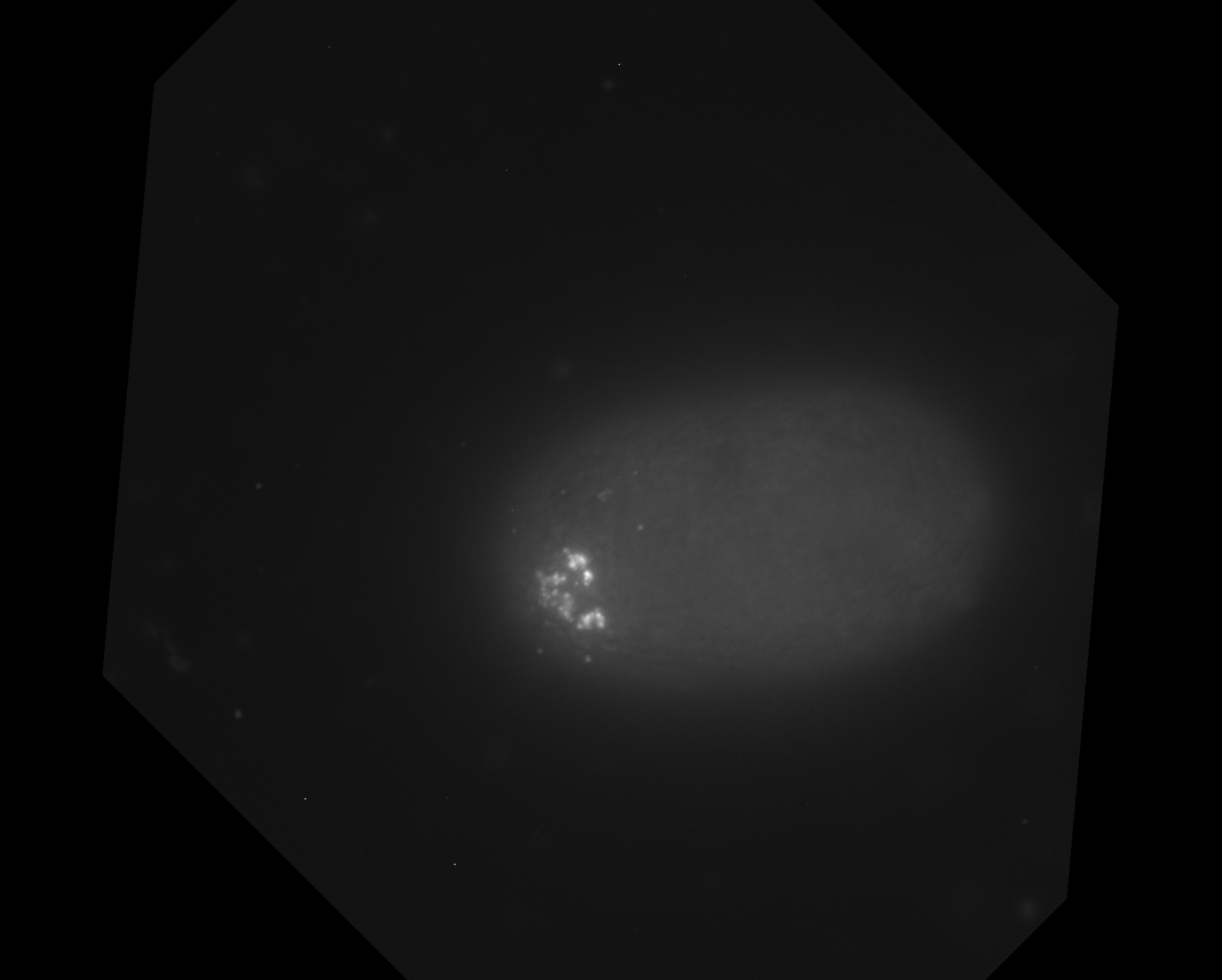

Supplement: Figure 3—figure supplement 1—source data 1. [file elife-85748-fig3-figsupp1-data1.zip › Figure3-figure-supplement1-Source_Data1/A-B/MAX_C1-Experiment-7220.tif]

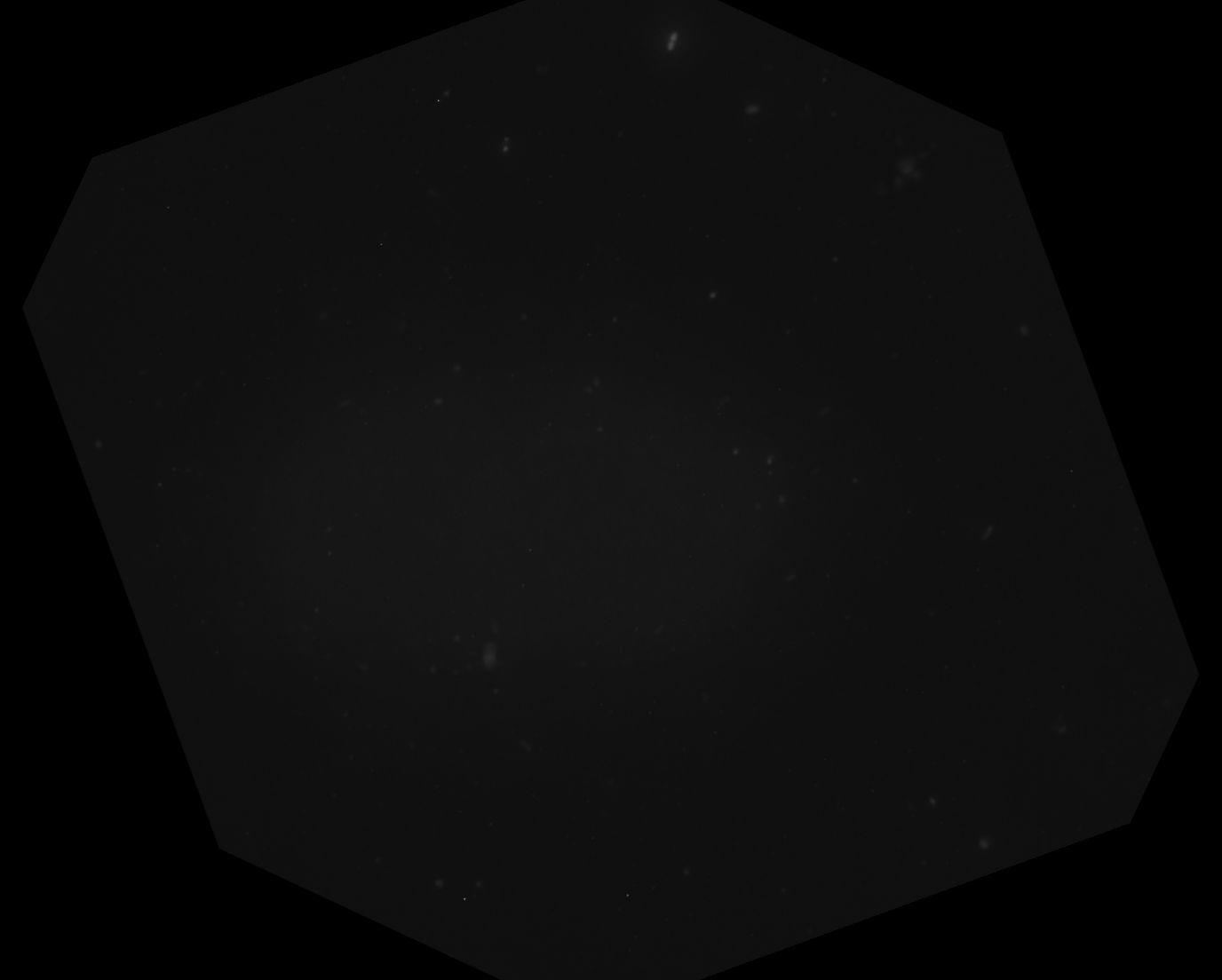

Supplement: Figure 3—figure supplement 1—source data 1. [file elife-85748-fig3-figsupp1-data1.zip › Figure3-figure-supplement1-Source_Data1/A-B/MAX_C1-Experiment-7223.tif]

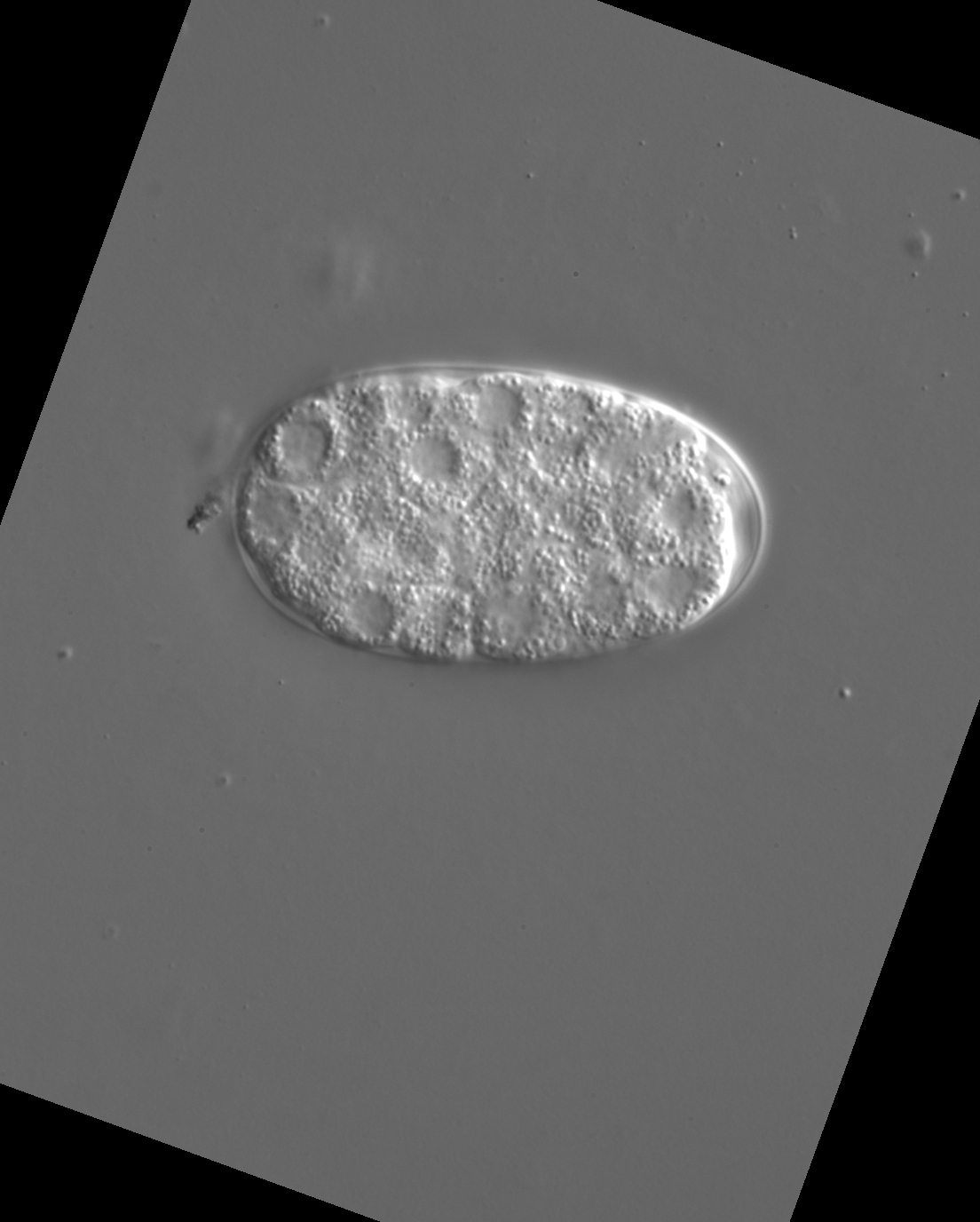

Supplement: Figure 3—figure supplement 1—source data 1. [file elife-85748-fig3-figsupp1-data1.zip › Figure3-figure-supplement1-Source_Data1/A-B/N2 C2-Experiment-7223.tif]

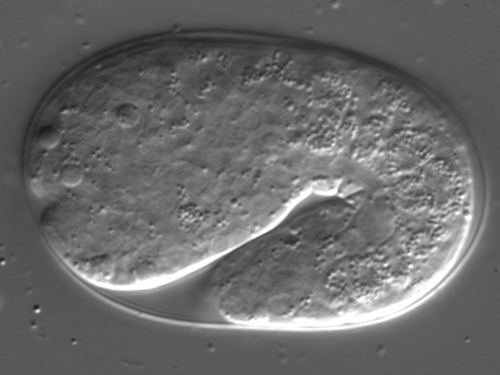

Supplement: Figure 3—figure supplement 1—source data 1. [file elife-85748-fig3-figsupp1-data1.zip › Figure3-figure-supplement1-Source_Data1/C/GASTOPEXp7036.tif]

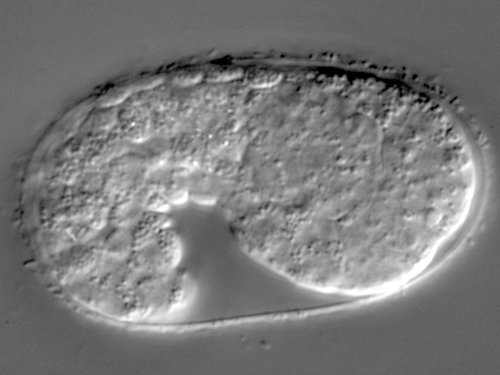

Supplement: Figure 3—figure supplement 1—source data 1. [file elife-85748-fig3-figsupp1-data1.zip › Figure3-figure-supplement1-Source_Data1/C/lgg-1(g116A) Exp6560.tif]

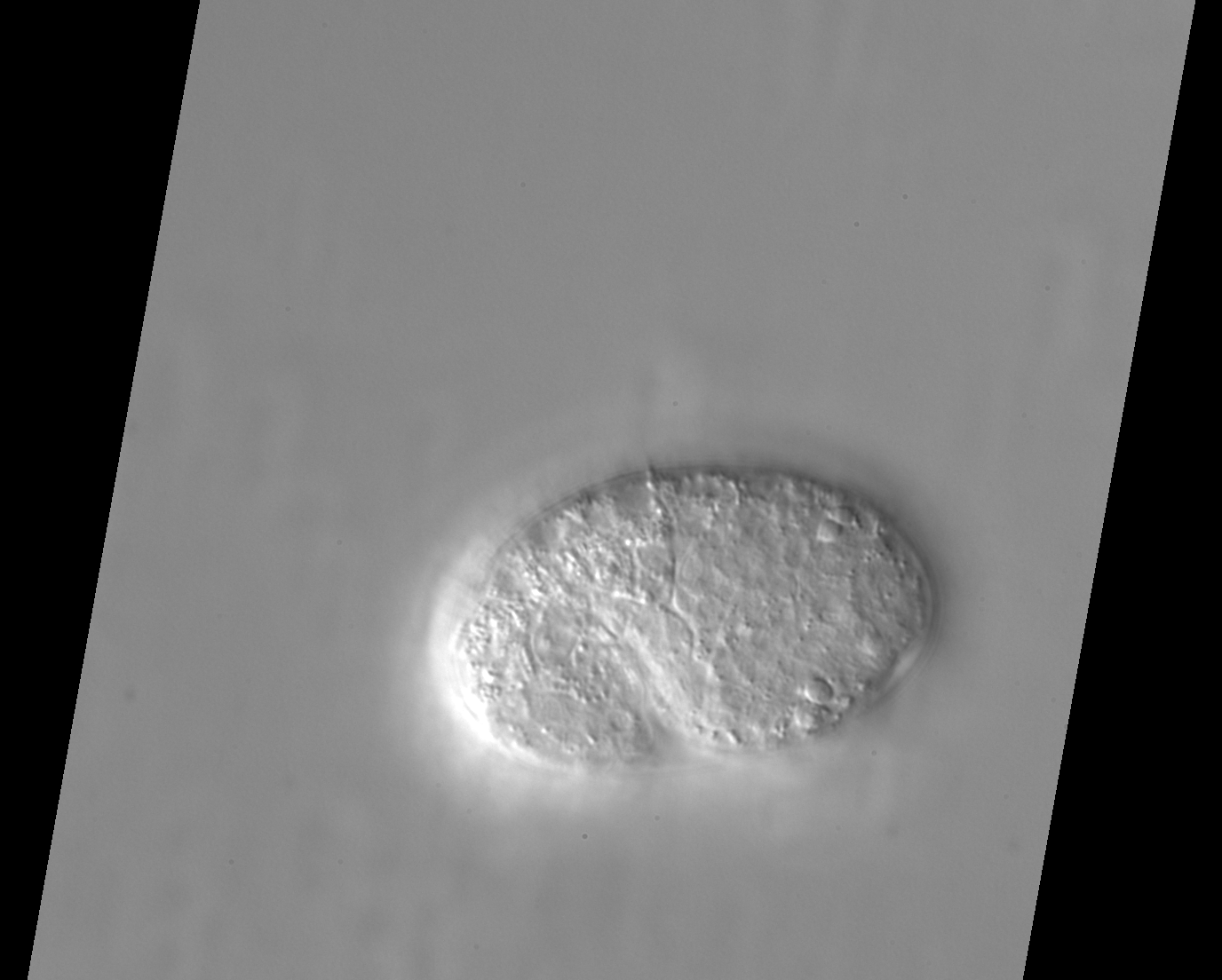

Supplement: Figure 3—figure supplement 1—source data 1. [file elife-85748-fig3-figsupp1-data1.zip › Figure3-figure-supplement1-Source_Data1/C/lgg-1(tm3489)Exp7063.tif]

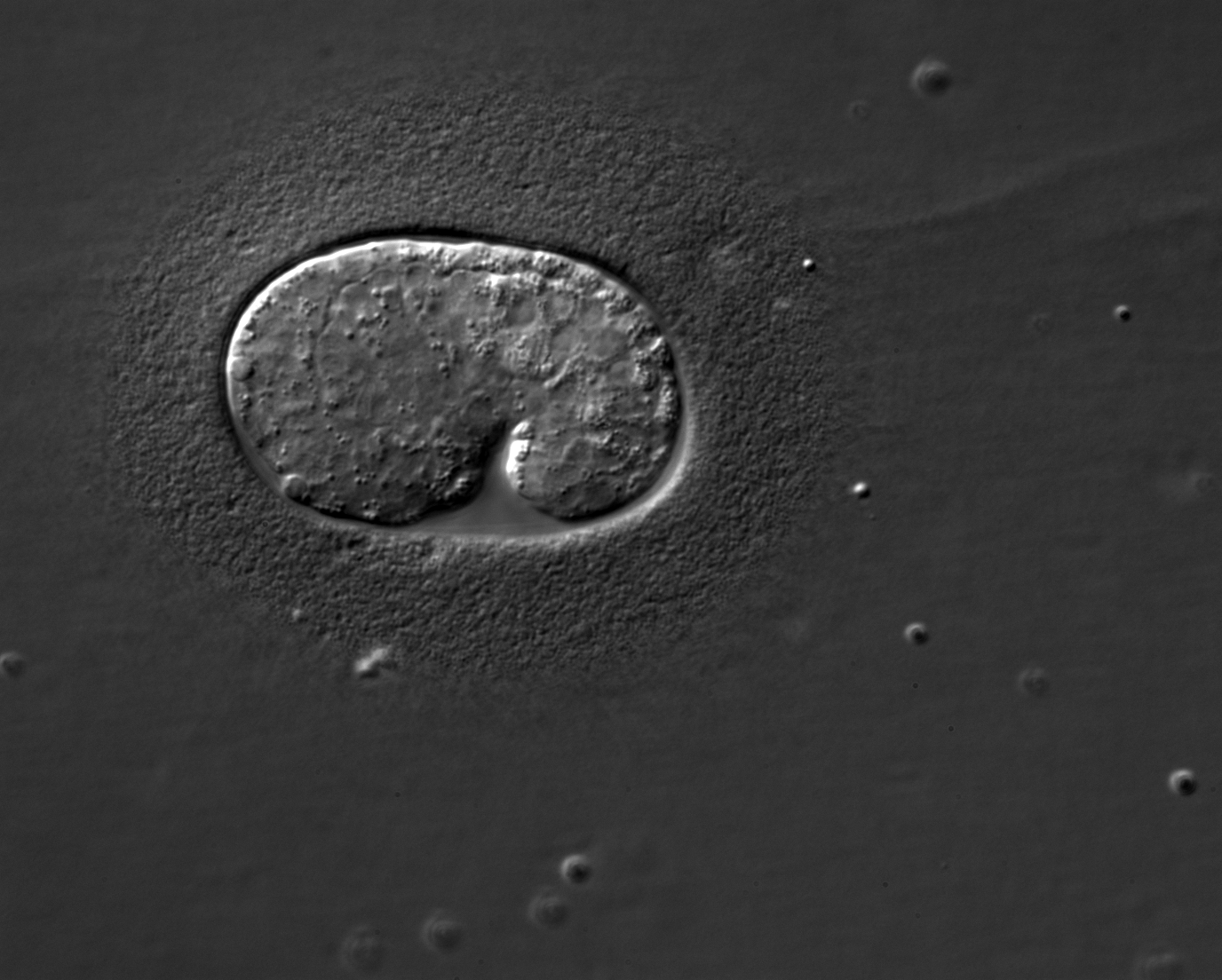

Supplement: Figure 3—figure supplement 1—source data 1. [file elife-85748-fig3-figsupp1-data1.zip › Figure3-figure-supplement1-Source_Data1/C/N2Exp7053.tif]

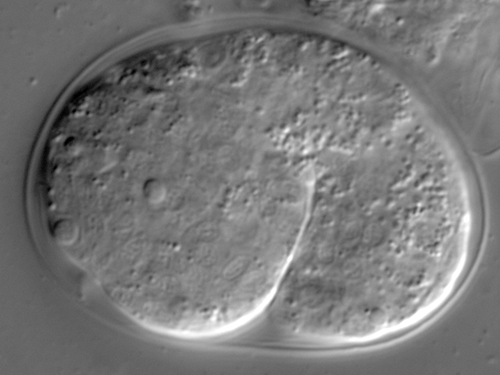

Supplement: Figure 3—figure supplement 1—source data 1. [file elife-85748-fig3-figsupp1-data1.zip › Figure3-figure-supplement1-Source_Data1/C/pp142Exp8639.tif]

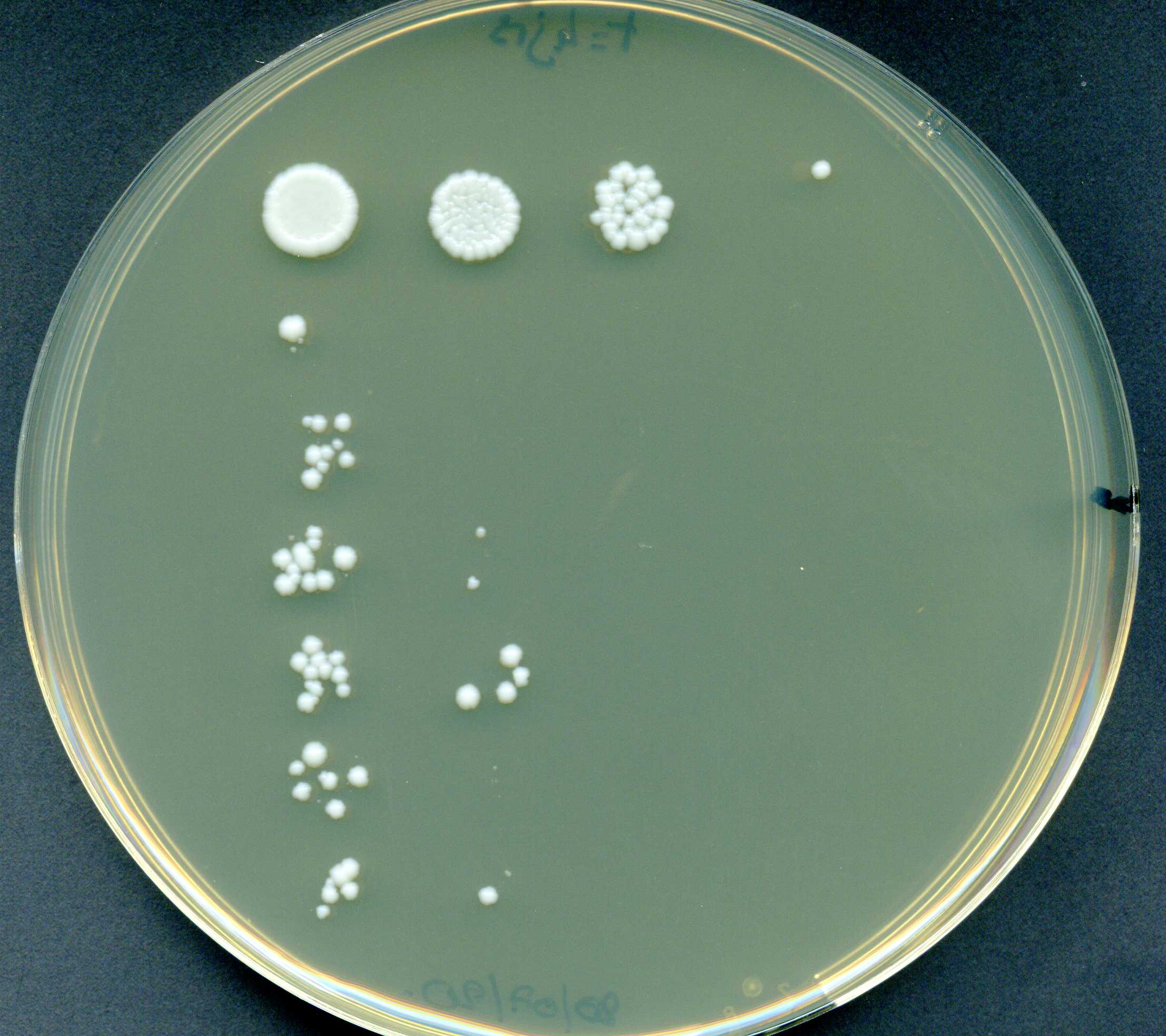

Supplement: Figure 3—figure supplement 2—source data 1. [file elife-85748-fig3-figsupp2-data1.zip › Figure3-figure-supplement2-Source_Data1/C/ATG8 t4j002.jpg]

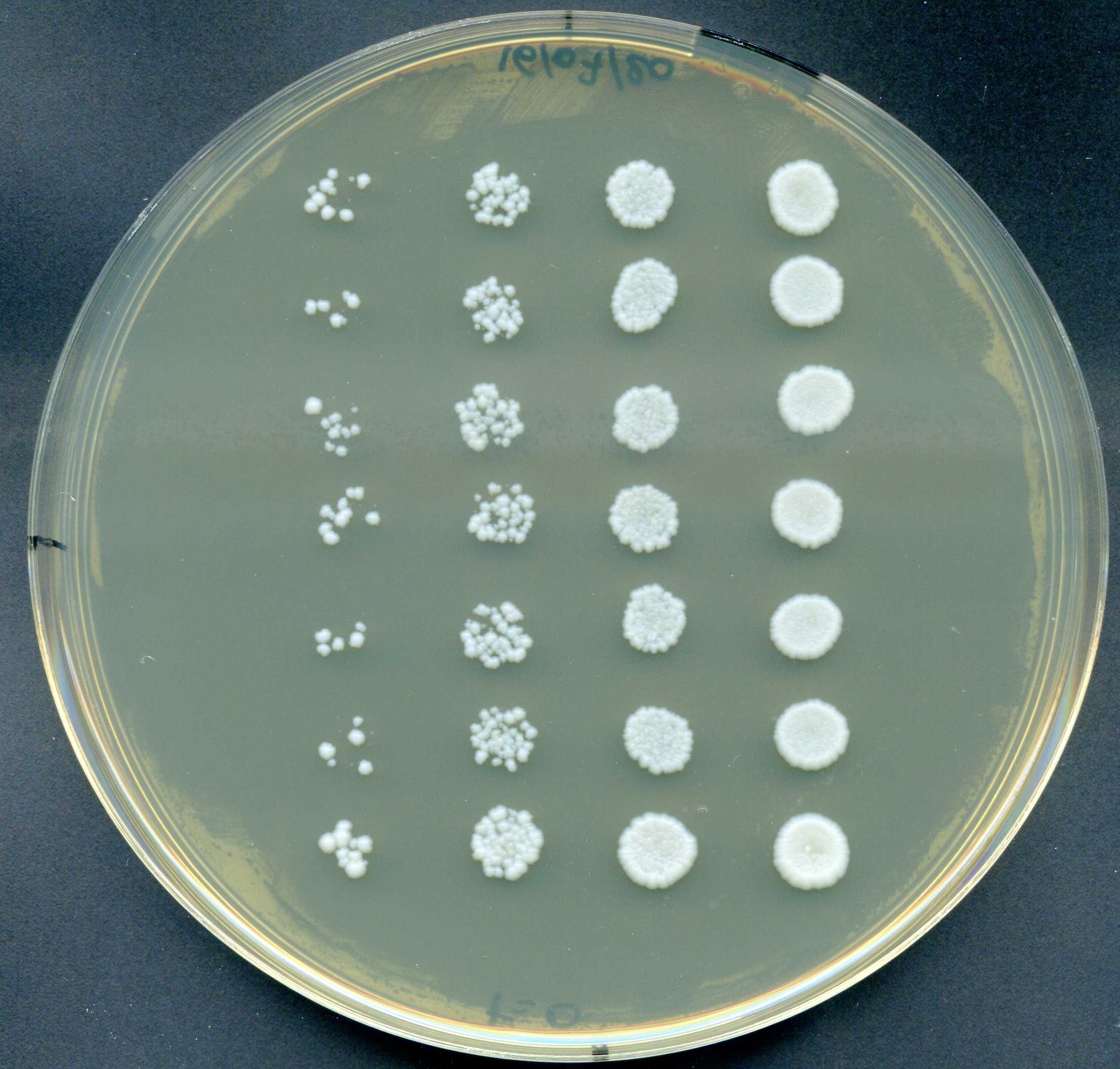

Supplement: Figure 3—figure supplement 2—source data 1. [file elife-85748-fig3-figsupp2-data1.zip › Figure3-figure-supplement2-Source_Data1/C/ATG8t0001.jpg]

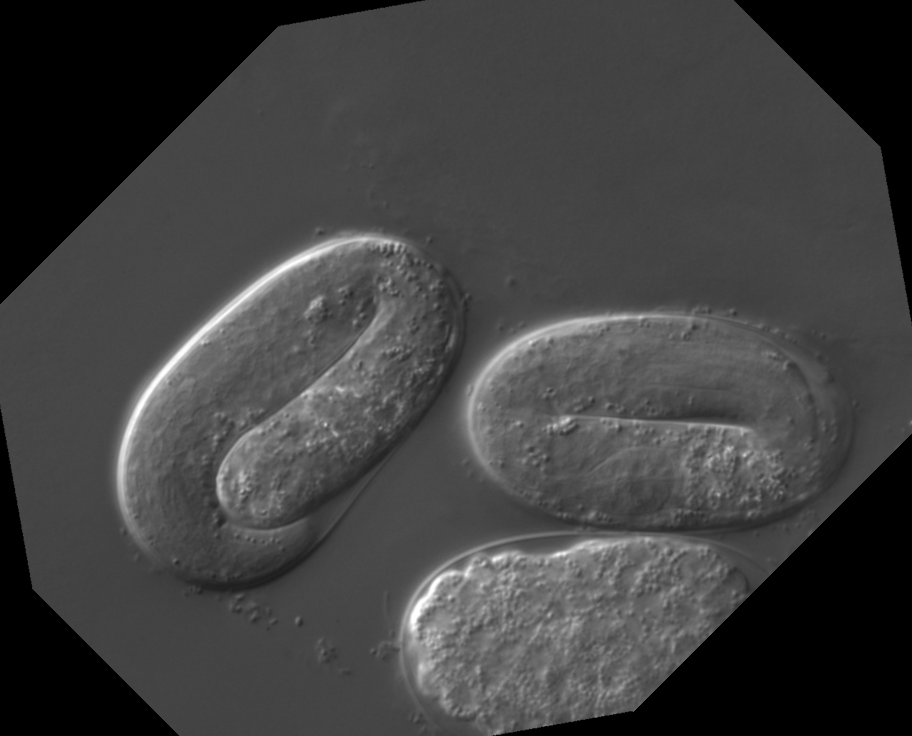

Supplement: Figure 4—source data 1. [file elife-85748-fig4-data1.zip › Figure4-Source_Data1/A-F/lgg-2(tm5755).tif]

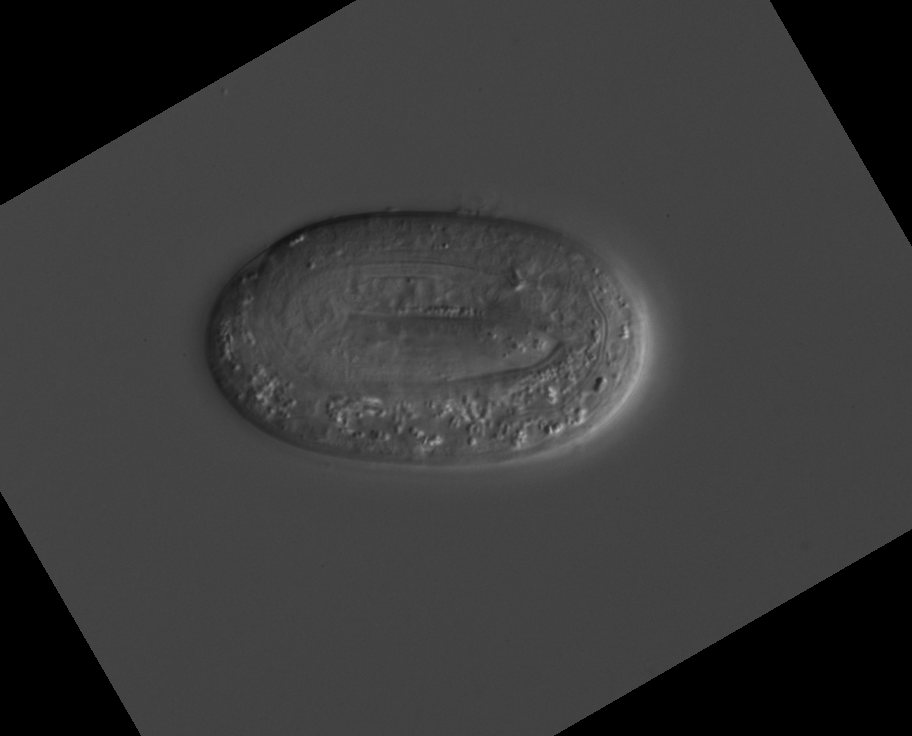

Supplement: Figure 4—source data 1. [file elife-85748-fig4-data1.zip › Figure4-Source_Data1/A-F/lgg-2(tm5755);lgg-1(G116A).tif]

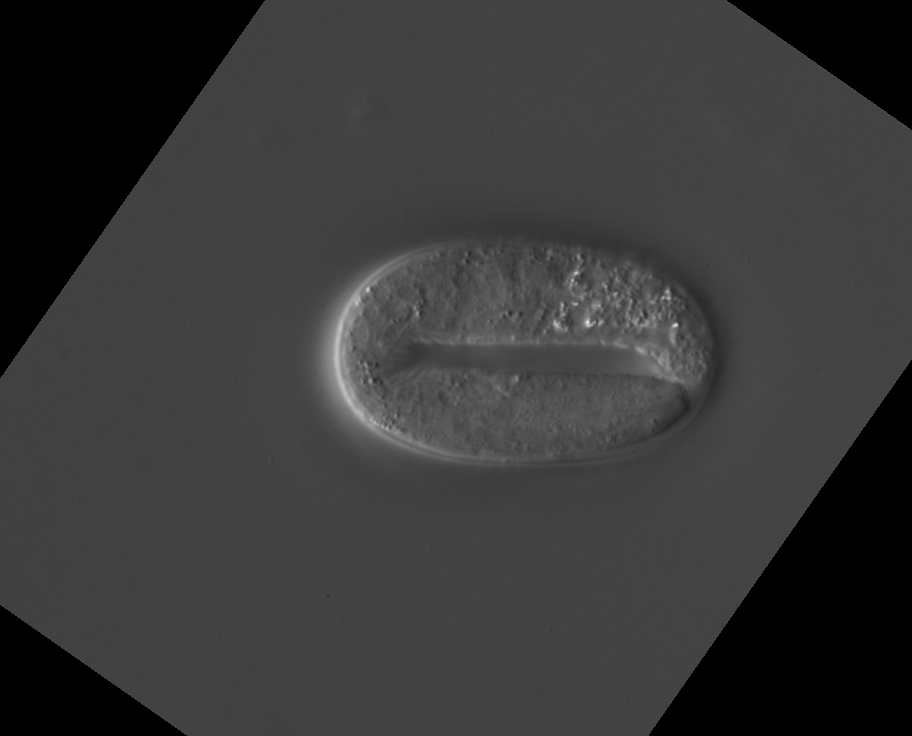

Supplement: Figure 4—source data 1. [file elife-85748-fig4-data1.zip › Figure4-Source_Data1/A-F/N2.tif]

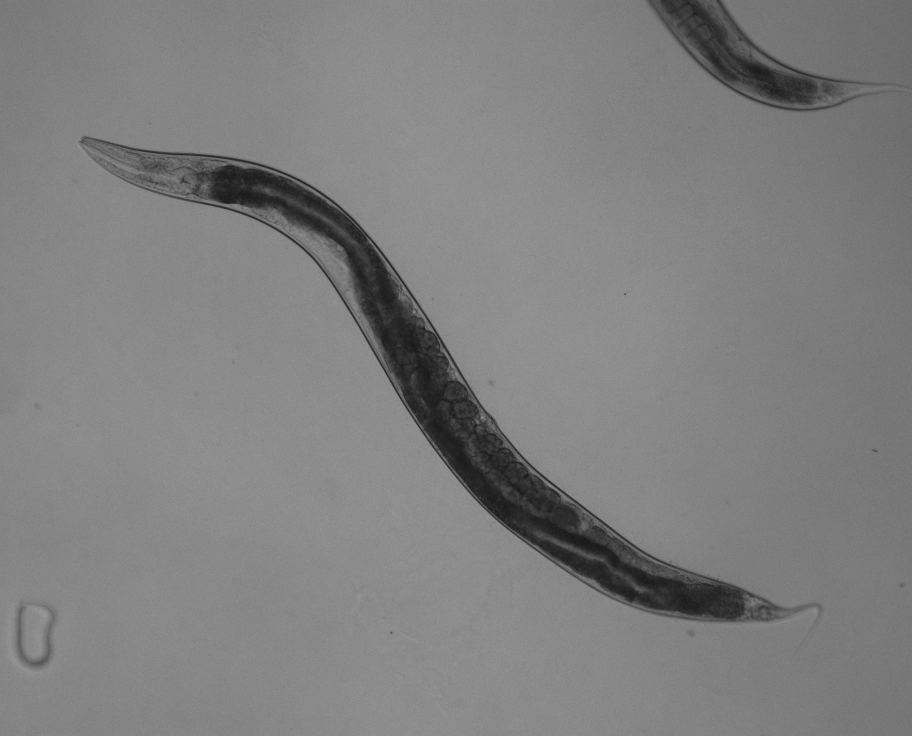

Supplement: Figure 4—source data 1. [file elife-85748-fig4-data1.zip › Figure4-Source_Data1/A-F/Snap-31980-scale.tif]

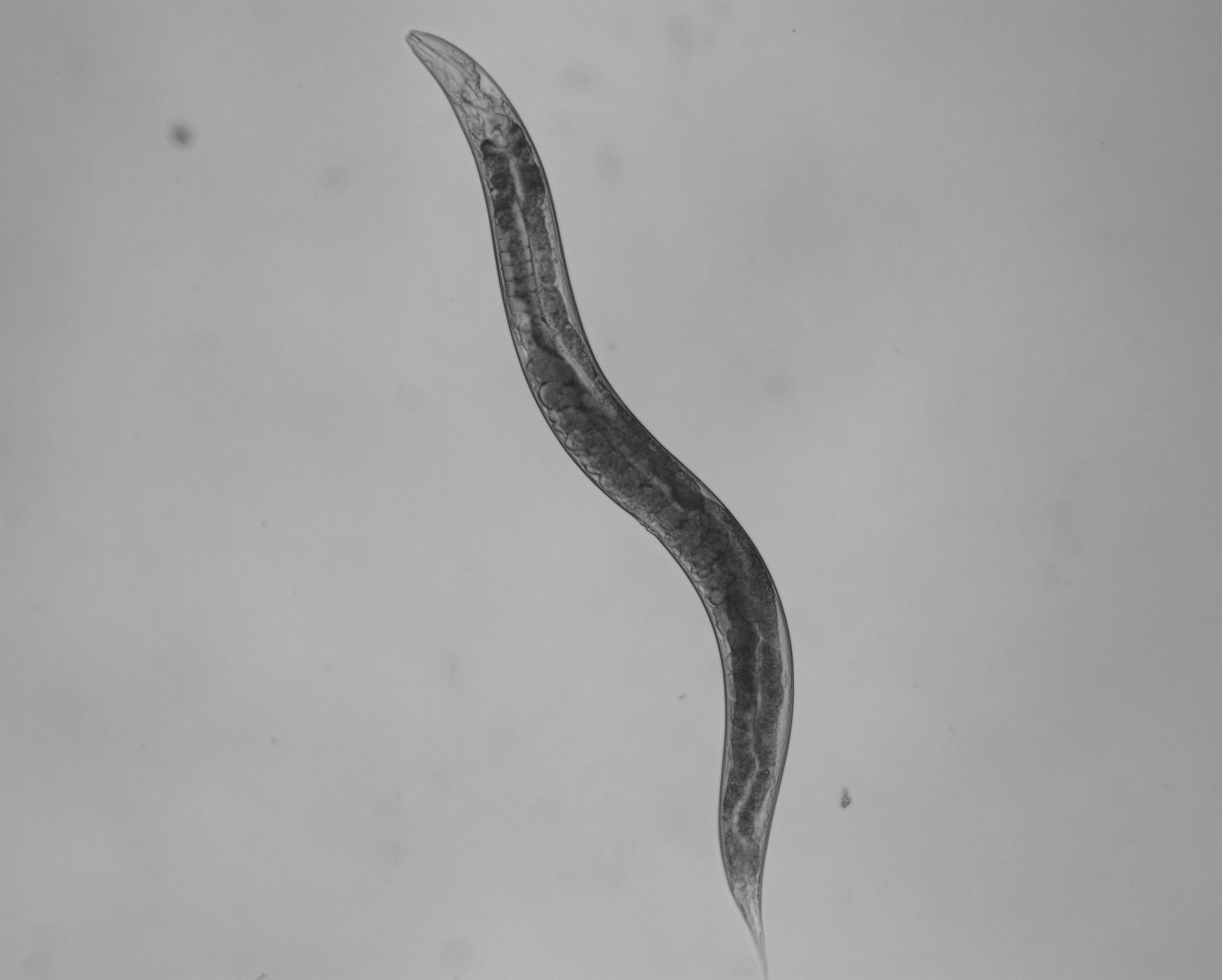

Supplement: Figure 4—source data 1. [file elife-85748-fig4-data1.zip › Figure4-Source_Data1/A-F/Snap-32489.tif]

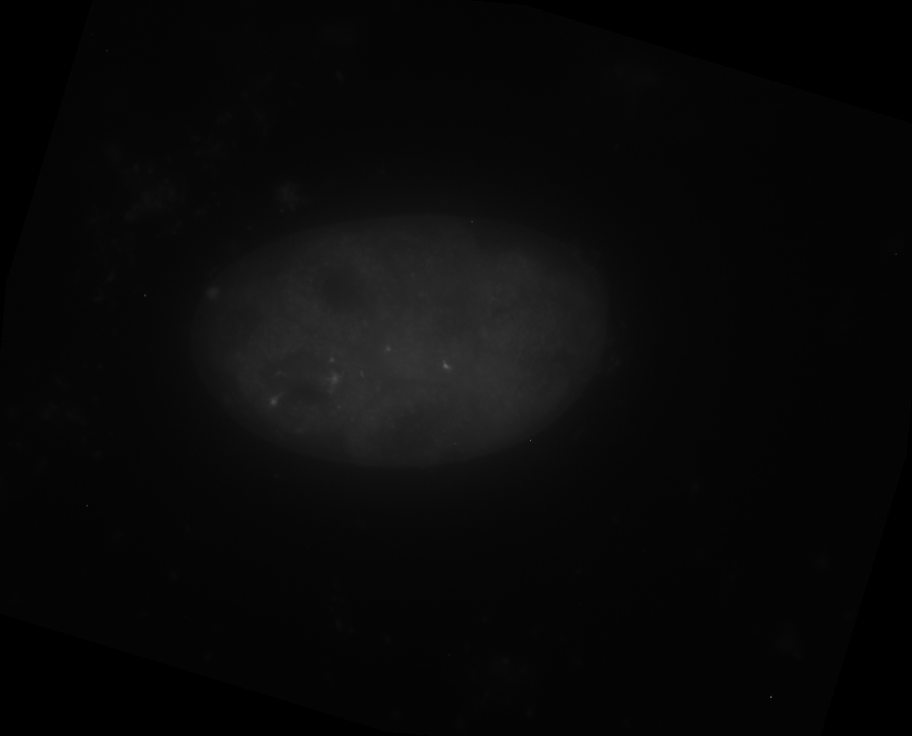

Supplement: Figure 4—source data 1. [file elife-85748-fig4-data1.zip › Figure4-Source_Data1/I-K/15C- lgg-2(tm5755)MAX_Experiment-6015-1.tif]

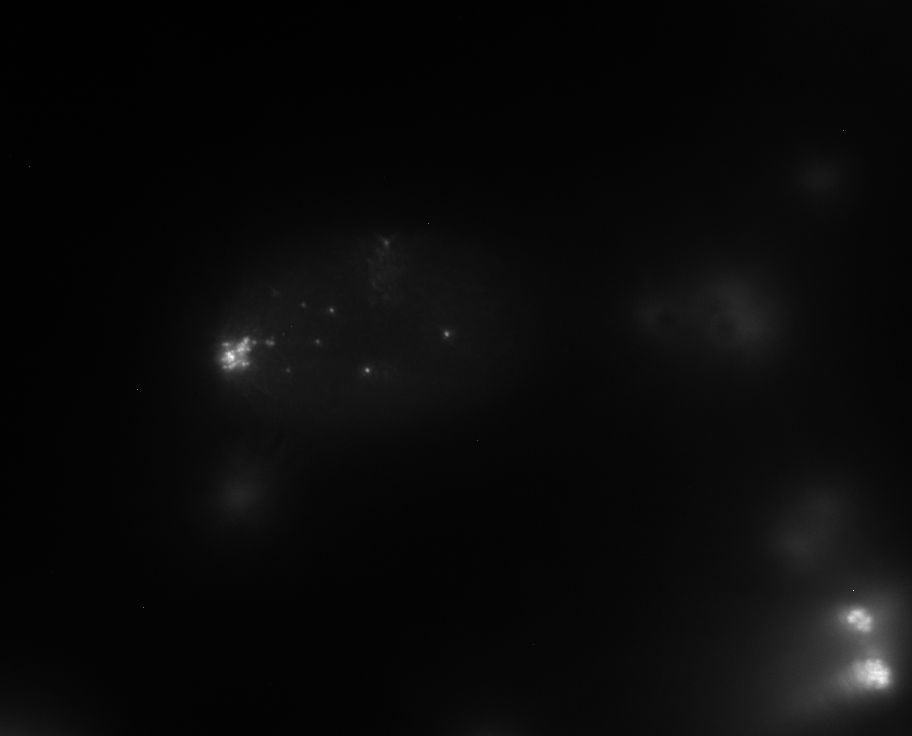

Supplement: Figure 4—source data 1. [file elife-85748-fig4-data1.zip › Figure4-Source_Data1/I-K/1C-hsp6-MAX_Experiment-6031-1.tif]

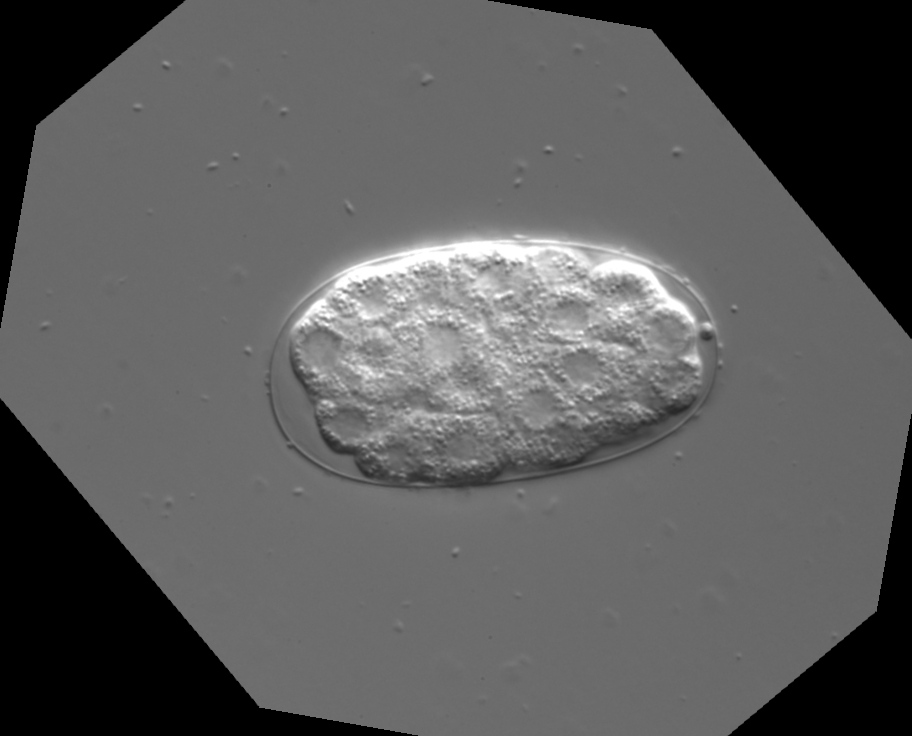

Supplement: Figure 4—source data 1. [file elife-85748-fig4-data1.zip › Figure4-Source_Data1/I-K/Experiment-5920-DIC1.tif]

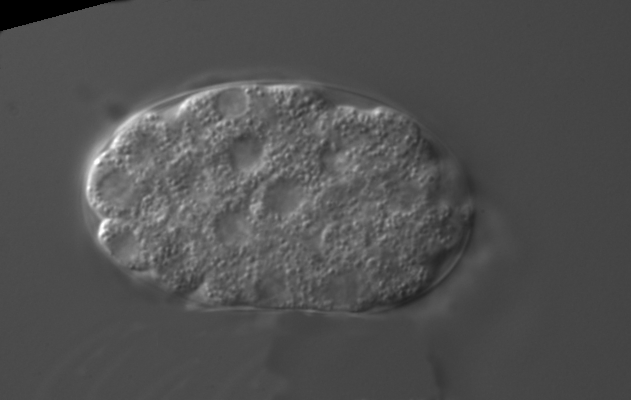

Supplement: Figure 4—source data 1. [file elife-85748-fig4-data1.zip › Figure4-Source_Data1/I-K/Experiment-DIC- 6198-3tif.tif]

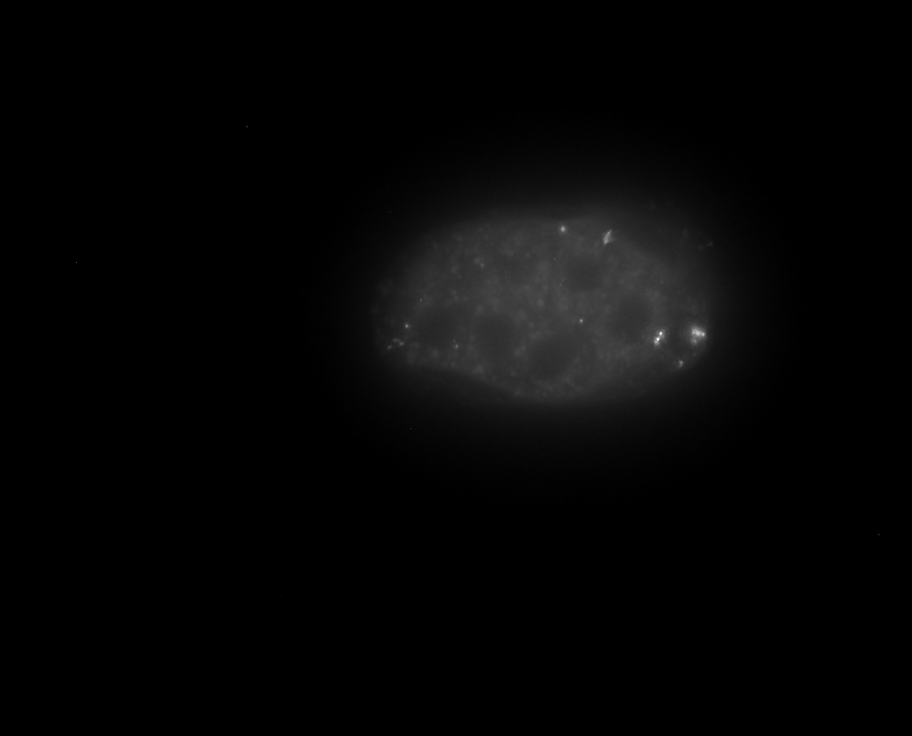

Supplement: Figure 4—source data 1. [file elife-85748-fig4-data1.zip › Figure4-Source_Data1/I-K/MAX_15C -hsp6-Experiment-5907-1.tif]

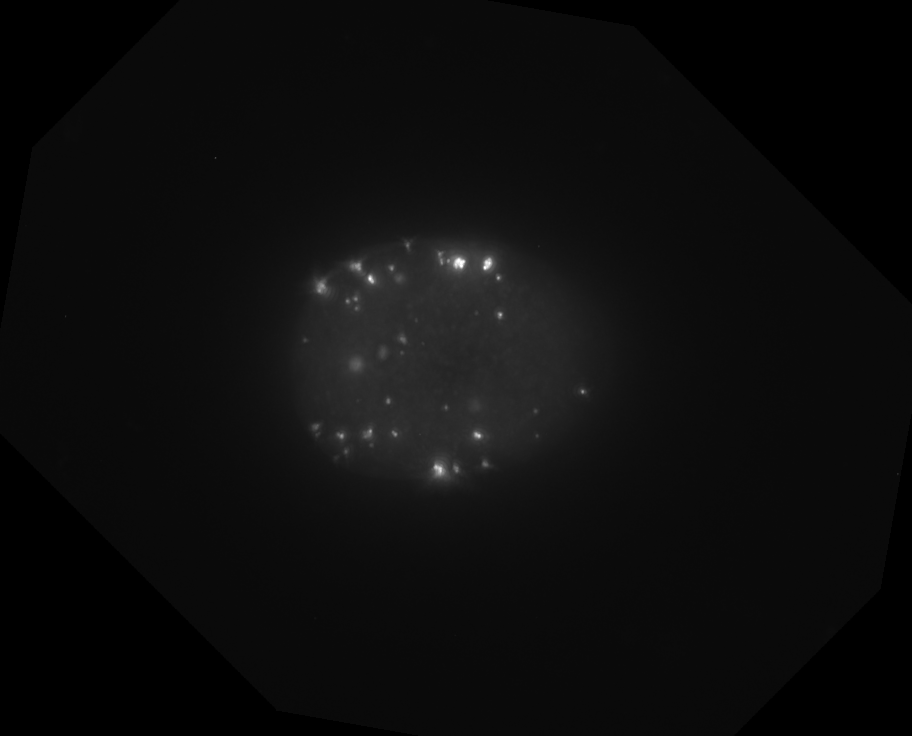

Supplement: Figure 4—source data 1. [file elife-85748-fig4-data1.zip › Figure4-Source_Data1/I-K/MAX_1C-hsp6-Experiment-5903.tif]

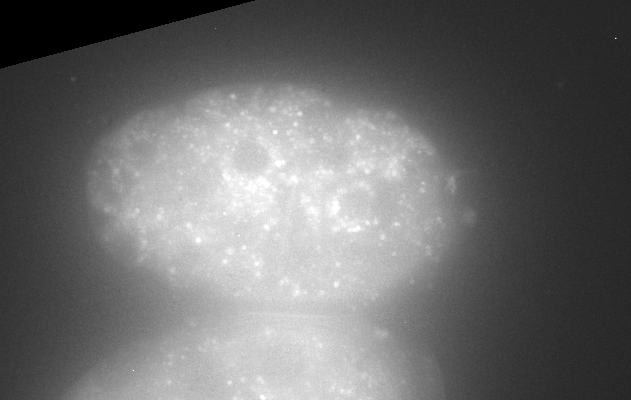

Supplement: Figure 4—source data 1. [file elife-85748-fig4-data1.zip › Figure4-Source_Data1/I-K/MAX_30C-Experiment-6198.tif]

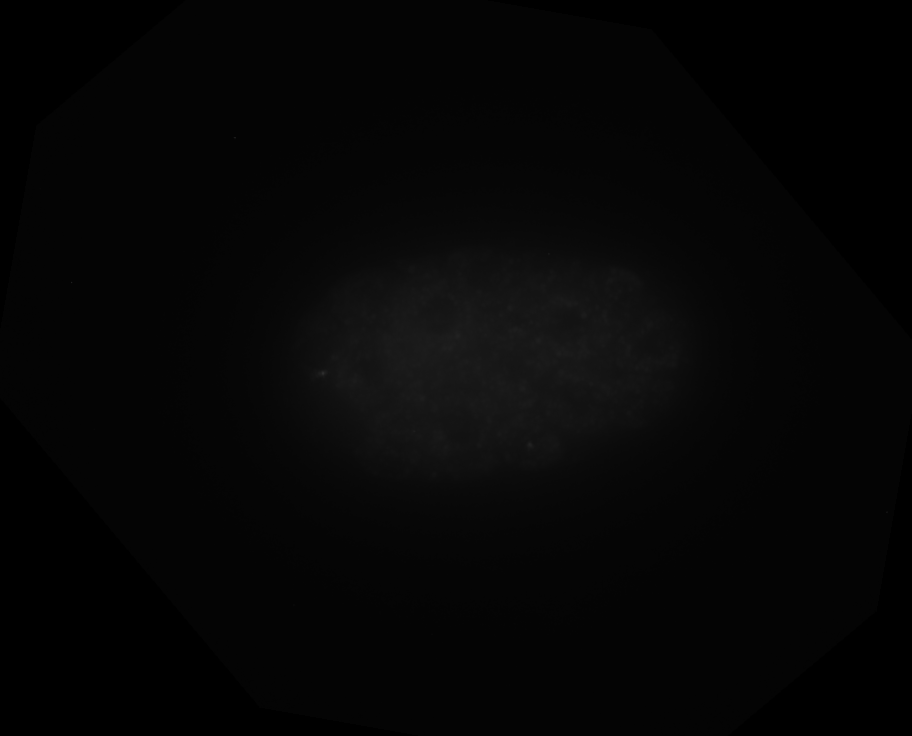

Supplement: Figure 4—source data 1. [file elife-85748-fig4-data1.zip › Figure4-Source_Data1/I-K/MAX_Experiment-5920-1.tif]

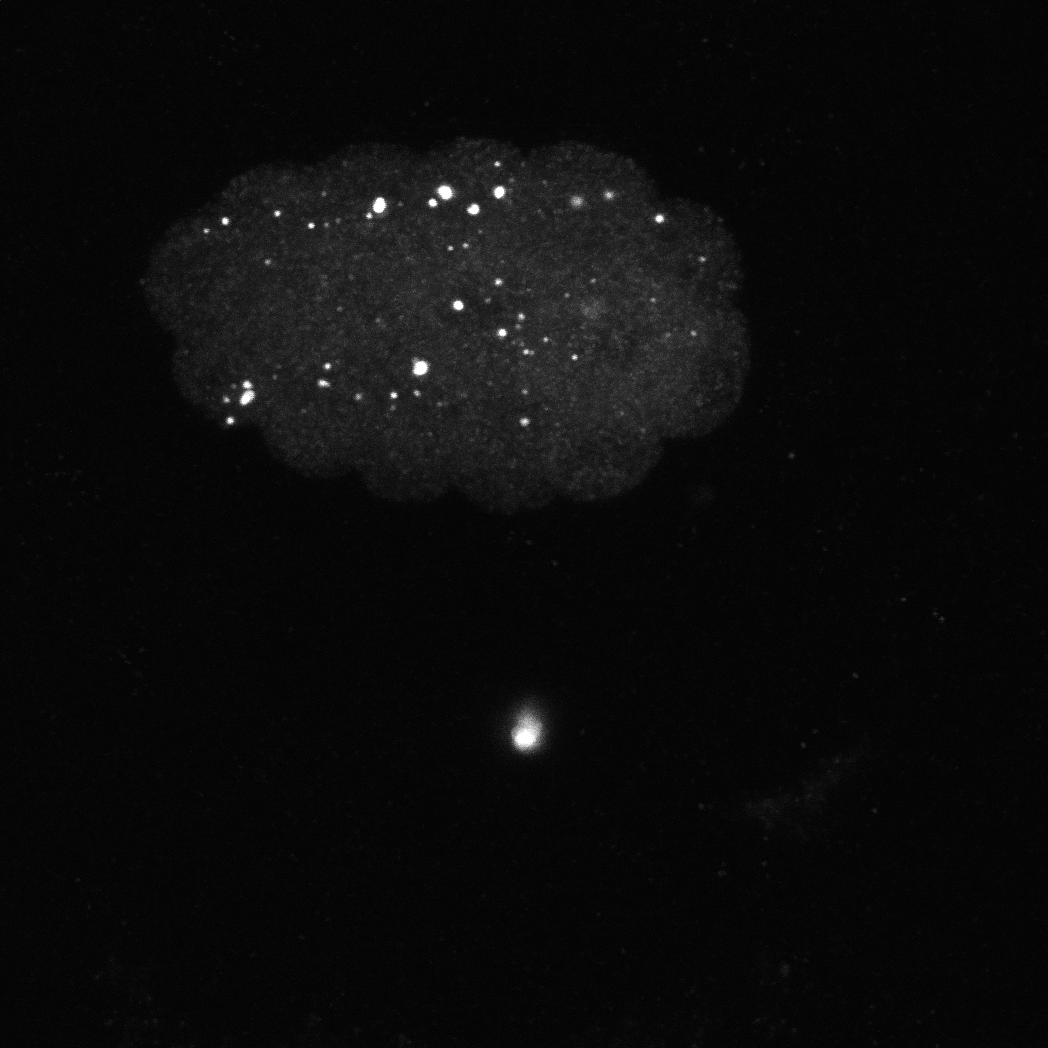

Supplement: Figure 5—source data 1. [file elife-85748-fig5-data1.zip › Figure5-Source_Data1/A-F/MAX_20200820-IF ablgg2 abgfp - qtg18gfp gastop.lif - Series018-1.tif]
