# Supplementary material for: LGG-1/GABARAP lipidation is not required for autophagy and development in Caenorhabditis elegans
Source: eLife. 2023 Jul 3;12:e85748. doi: 10.7554/eLife.85748 (PMC10338037; doi:10.7554/eLife.85748)
Supplement: Figure 4—source data 1. [file elife-85748-fig4-data1.zip › Figure4-Source_Data1/G-H/Longévité .pdf]

Survival analysis by Kaplan-Meier curves and log-rank (Mantel-Cox) test

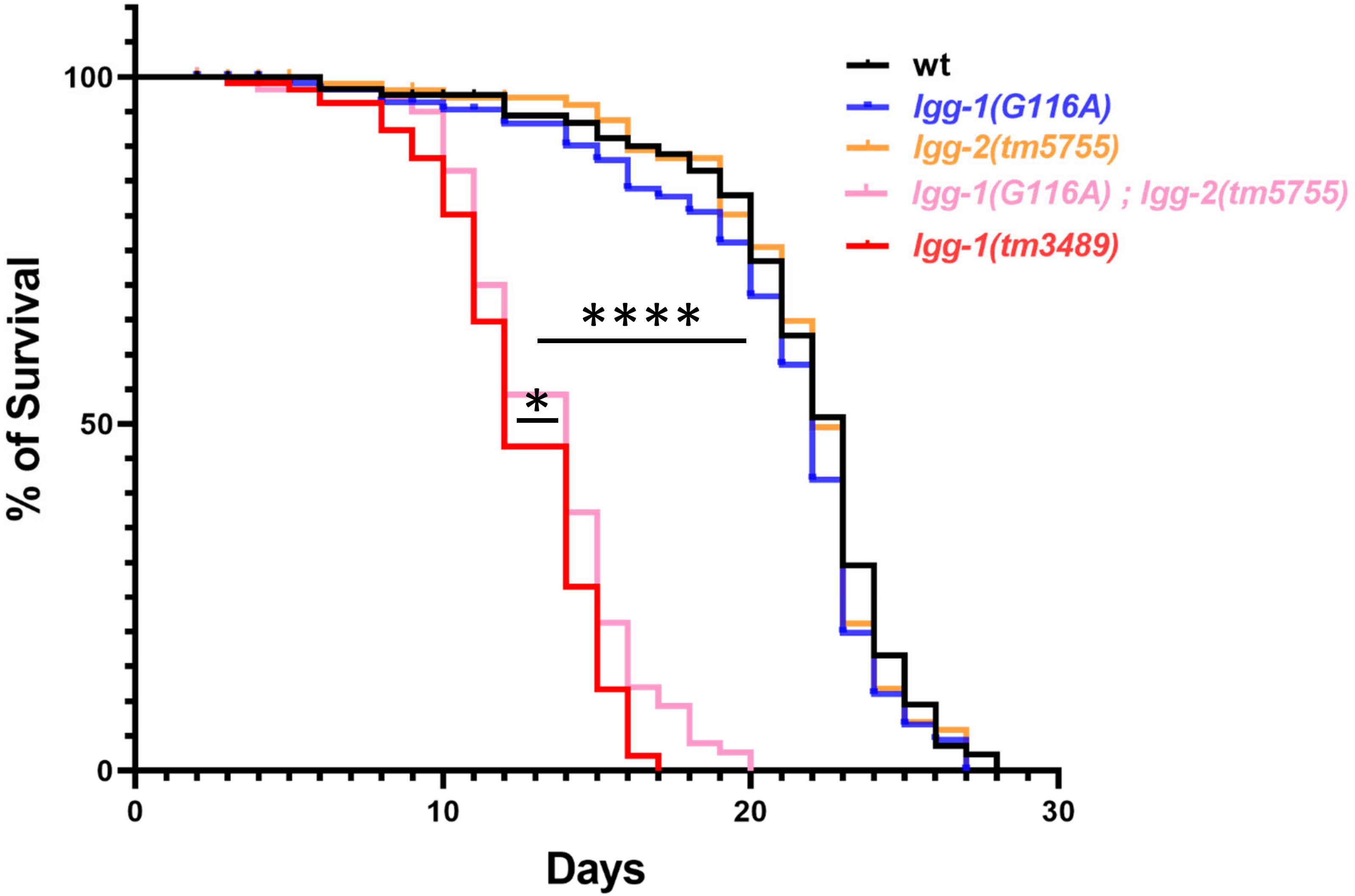

lgg-1(tm3489) vs *lgg-1(G116A); lgg-2(tm5755)*

Comparison of Survival Curves	
**Log-rank (Mantel-Cox) test**	
Chi square	5.936
df	1
P value	0.0148
P value summary	*
Are the survival curves sig different?	Yes
**Gehan-Breslow-Wilcoxon test**	
Chi square	3.056
df	1
P value	0.0804
P value summary	ns
Are the survival curves sig different?	No

lgg-1(tm3489) vs *wt*

Comparison of Survival Curves	
**Log-rank (Mantel-Cox) test**	
Chi square	174.0
df	1
P value	<0.0001
P value summary	****
Are the survival curves sig different'	Yes
**Gehan-Breslow-Wilcoxon test**	
Chi square	131.5
df	1
P value	<0.0001
P value summary	****
Are the survival curves sig different'	Yes

wt vs *lgg-1(G116A)*

Comparison of Survival Curves	
**Log-rank (Mantel-Cox) test**	
Chi square	1.927
df	1
P value	0.1651
P value summary	ns
Are the survival curves sig different'	No
**Gehan-Breslow-Wilcoxon test**	
Chi square	1.977
df	1
P value	0.1597
P value summary	ns
Are the survival curves sig different'	No

wt vs *lgg-2(tm5755)*

Comparison of Survival Curves	
**Log-rank (Mantel-Cox) test**	
Chi square	0.2874
df	1
P value	0.5919
P value summary	ns
Are the survival curves sig different?	No
**Gehan-Breslow-Wilcoxon test**	
Chi square	0.1008
df	1
P value	0.7509
P value summary	ns
Are the survival curves sig different?	No

lgg-1(G116A) vs *lgg-2(tm5755)*

Comparison of Survival Curves	
**Log-rank (Mantel-Cox) test**	
Chi square	0.8623
df	1
P value	0.3531
P value summary	ns
Are the survival curves sig different?	No
**Gehan-Breslow-Wilcoxon test**	
Chi square	1.340
df	1
P value	0.2470
P value summary	ns
Are the survival curves sig different?	No
