# Supplementary figures and images for: LGG-1/GABARAP lipidation is not required for autophagy and development in Caenorhabditis elegans (part 2 of 2)
Source: eLife. 2023 Jul 3;12:e85748. doi: 10.7554/eLife.85748 (PMC10338037; doi:10.7554/eLife.85748)

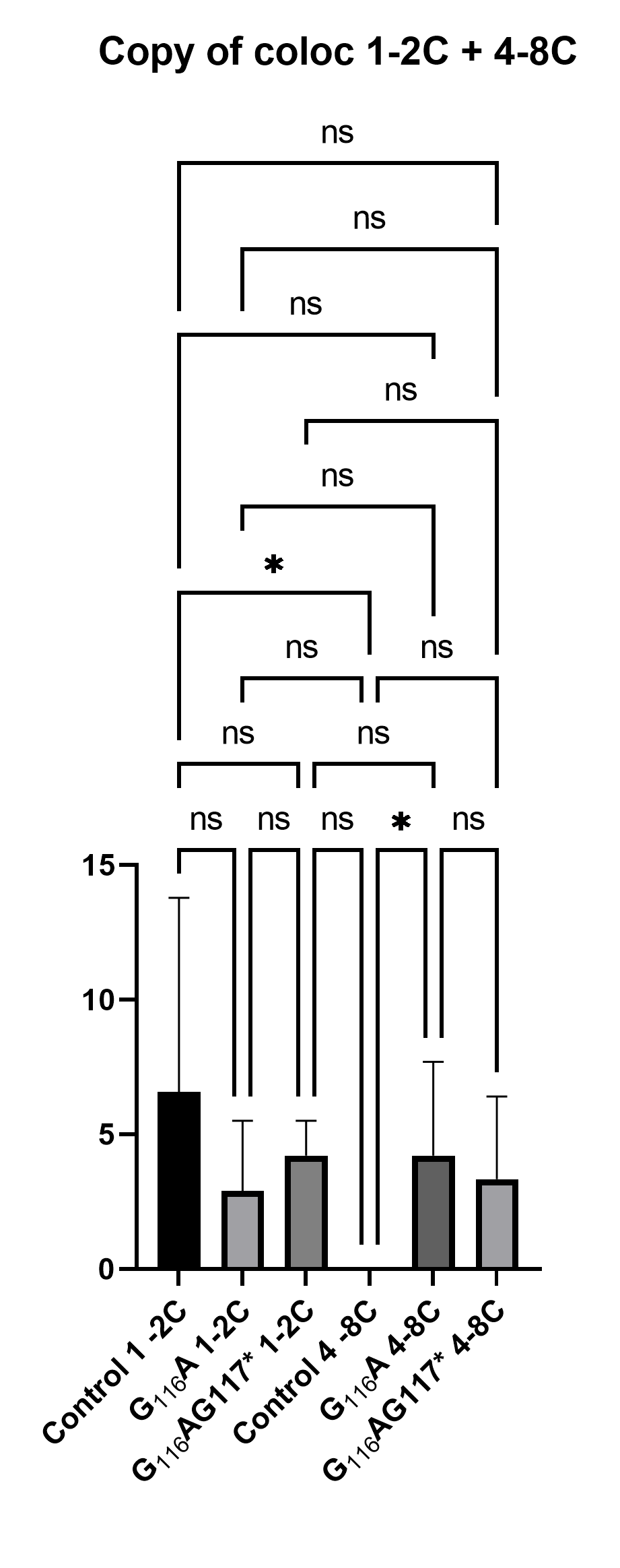

Supplement: Figure 5—source data 1. [file elife-85748-fig5-data1.zip › Figure5-Source_Data1/G-I/number of coloc between LGG-2 and hsp6 1-2C + 4-8C.tif]

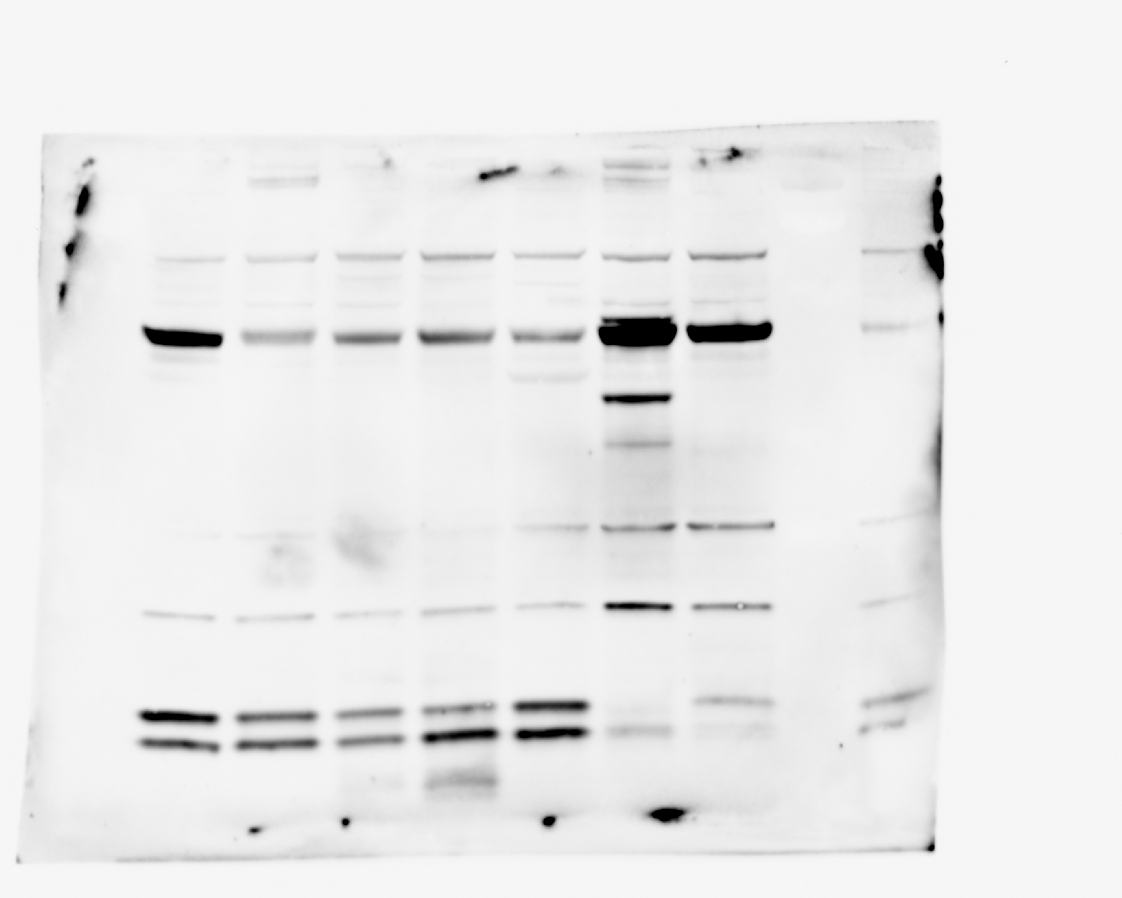

Supplement: Figure 5—source data 1. [file elife-85748-fig5-data1.zip › Figure5-Source_Data1/K/WB_LGG2_users 2019-12-13 19h03m37s(Chemiluminescence).tif]

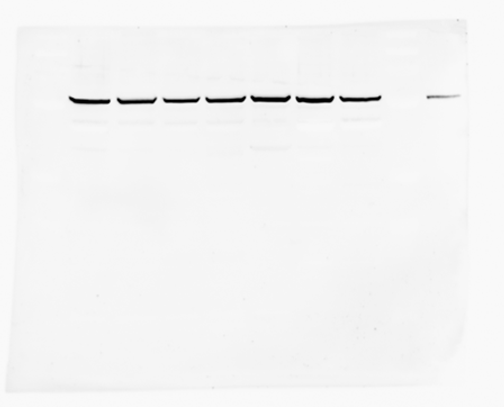

Supplement: Figure 5—source data 1. [file elife-85748-fig5-data1.zip › Figure5-Source_Data1/K/WB-Tubulin.tif]

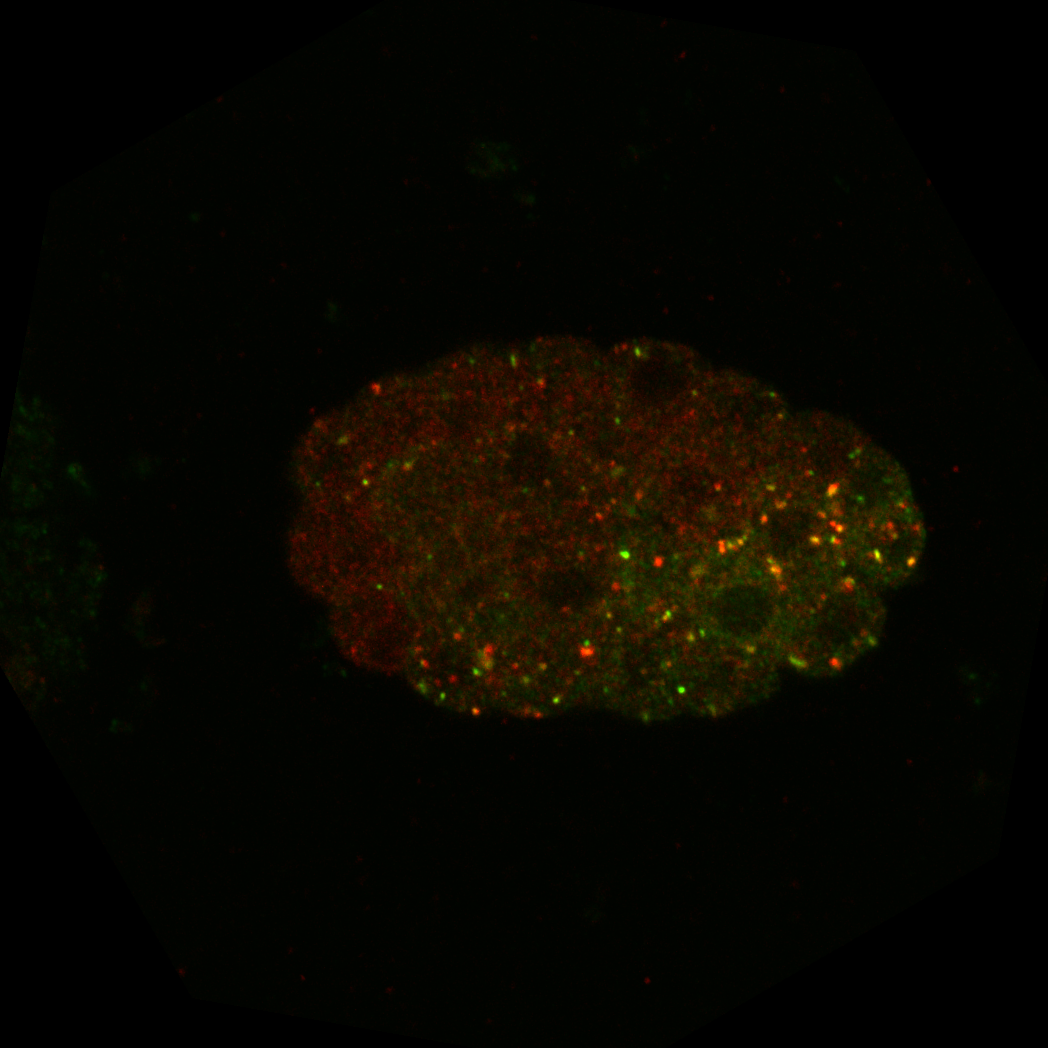

Supplement: Figure 7—source data 1. [file elife-85748-fig7-data1.zip › Figure7-Source_Data1/A-E/20200820-IF ablgg2 abgfp - atg18gfp controle.lif - Series009-1.tif (RGB).tif]

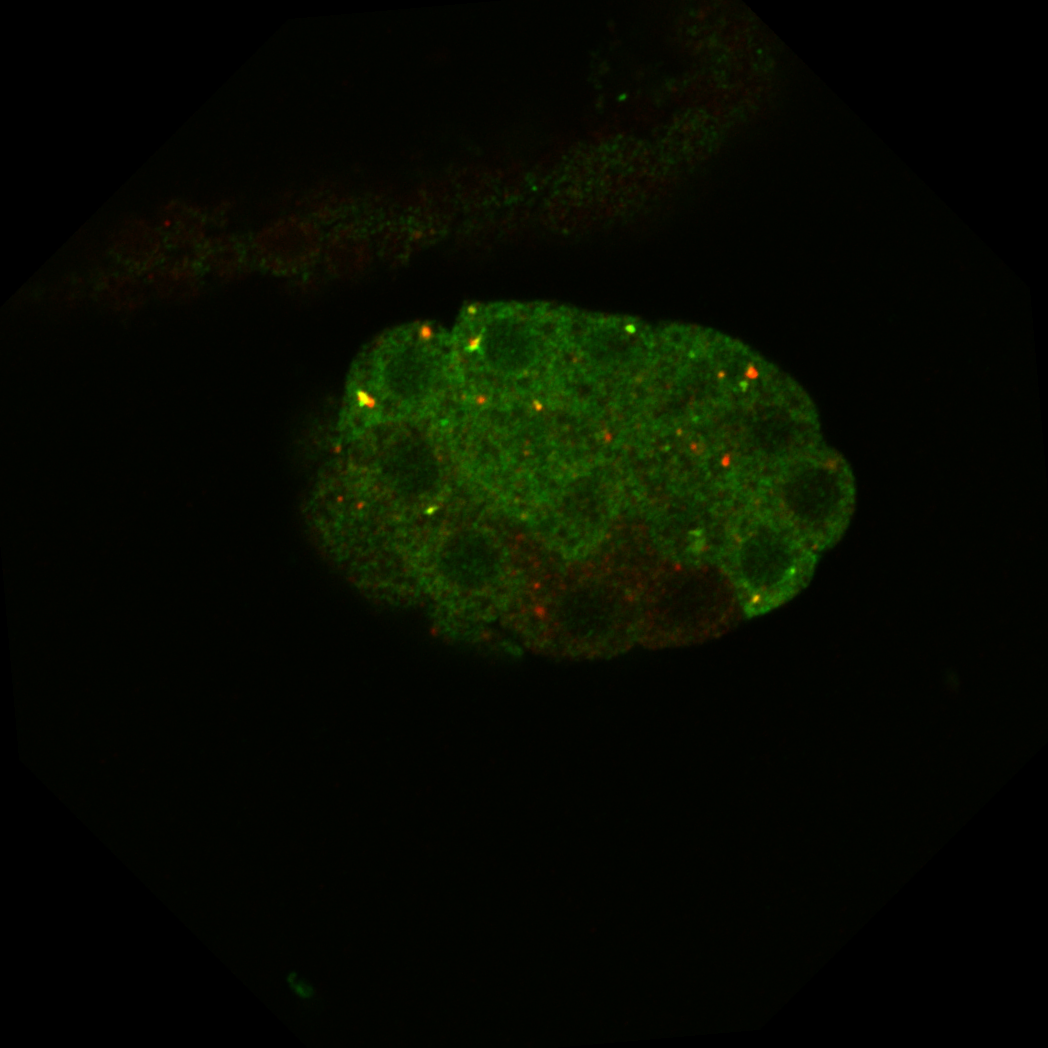

Supplement: Figure 7—source data 1. [file elife-85748-fig7-data1.zip › Figure7-Source_Data1/A-E/20200820-IF ablgg2 abgfp - qtg18gfp ga.lif - Series018-2.tif (RGB)-1.tif]

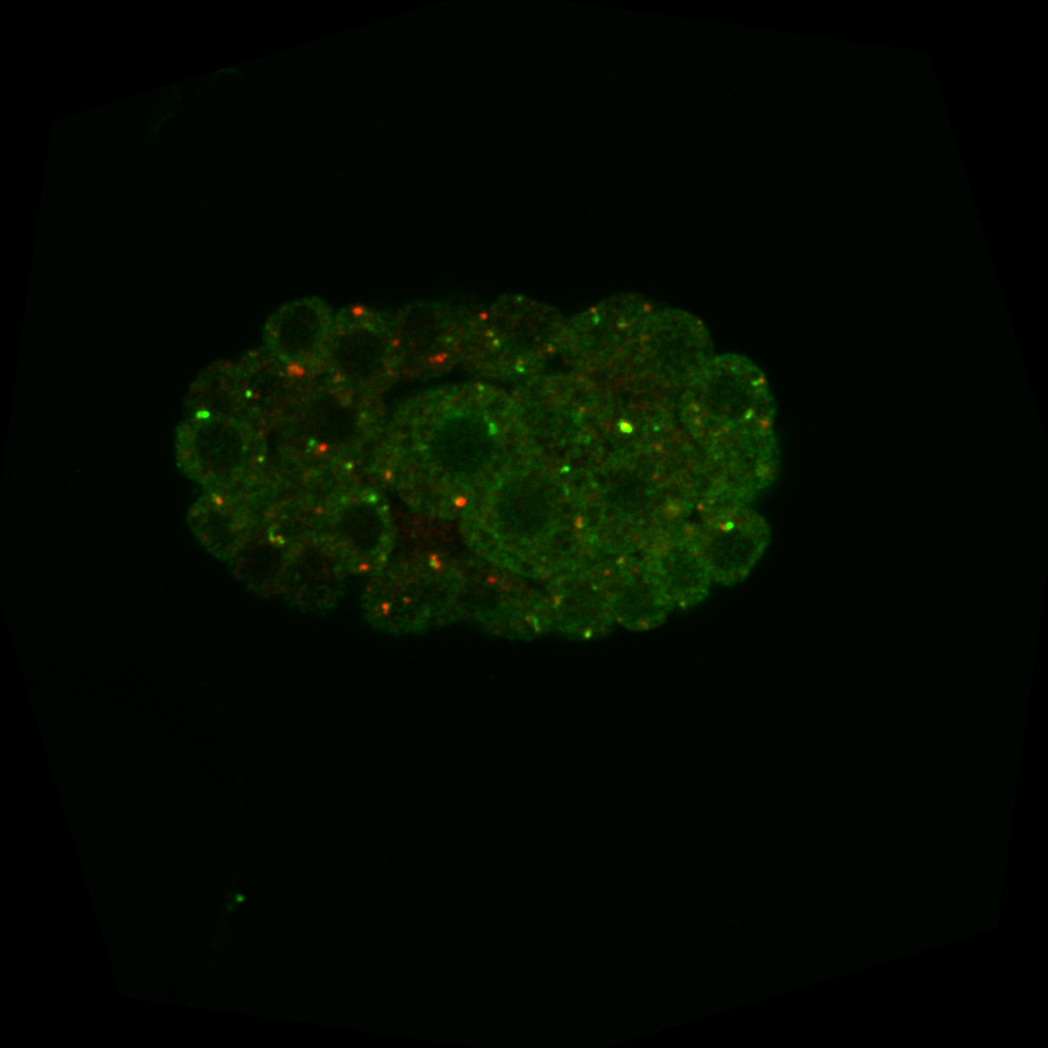

Supplement: Figure 7—source data 1. [file elife-85748-fig7-data1.zip › Figure7-Source_Data1/A-E/20200820-IF ablgg2 abgfp - qtg18gfp gastop.lif - Series026-1.tif (RGB).tif]

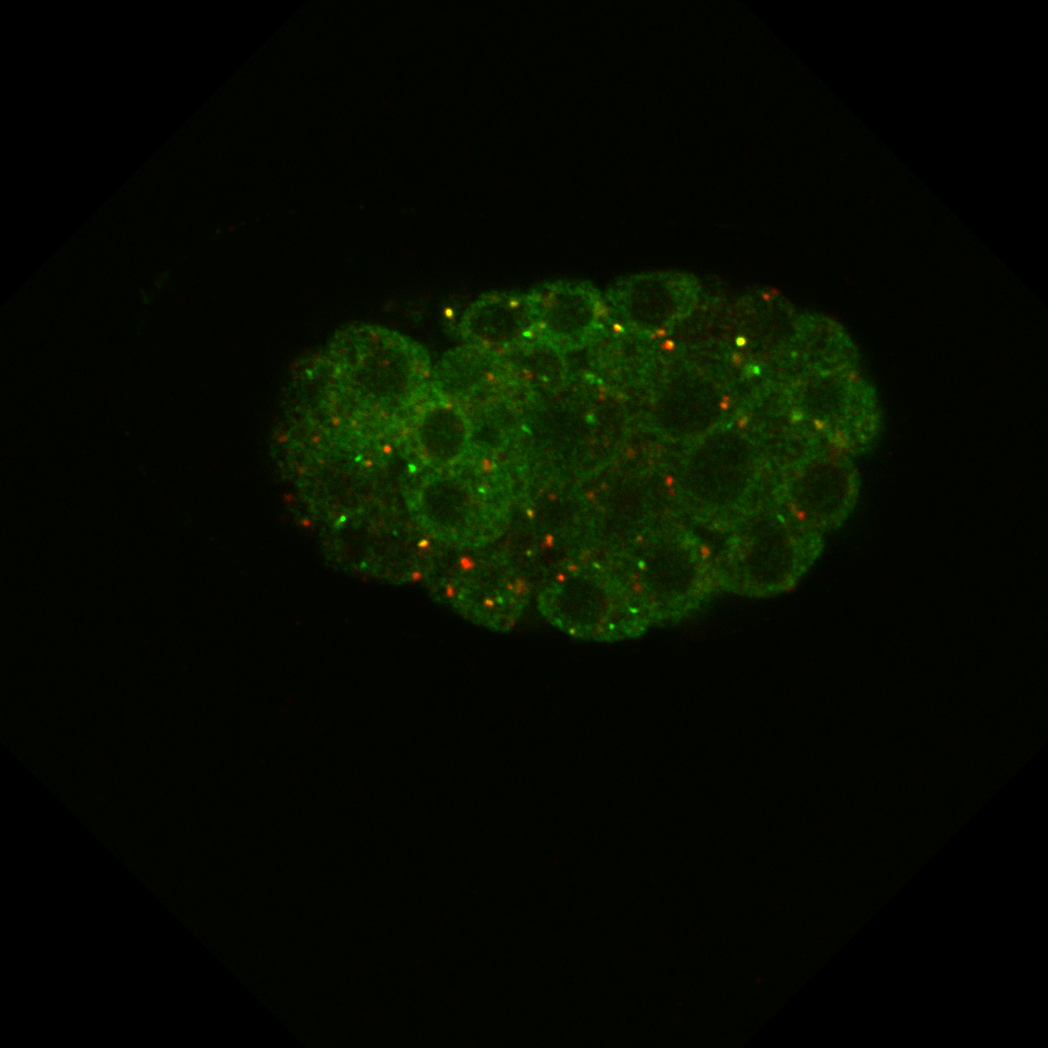

Supplement: Figure 7—source data 1. [file elife-85748-fig7-data1.zip › Figure7-Source_Data1/A-E/20200820-IF ablgg2 abgfp - qtg18gfp rnai lgg-1.lif - Series017-1.tif (RGB).tif]

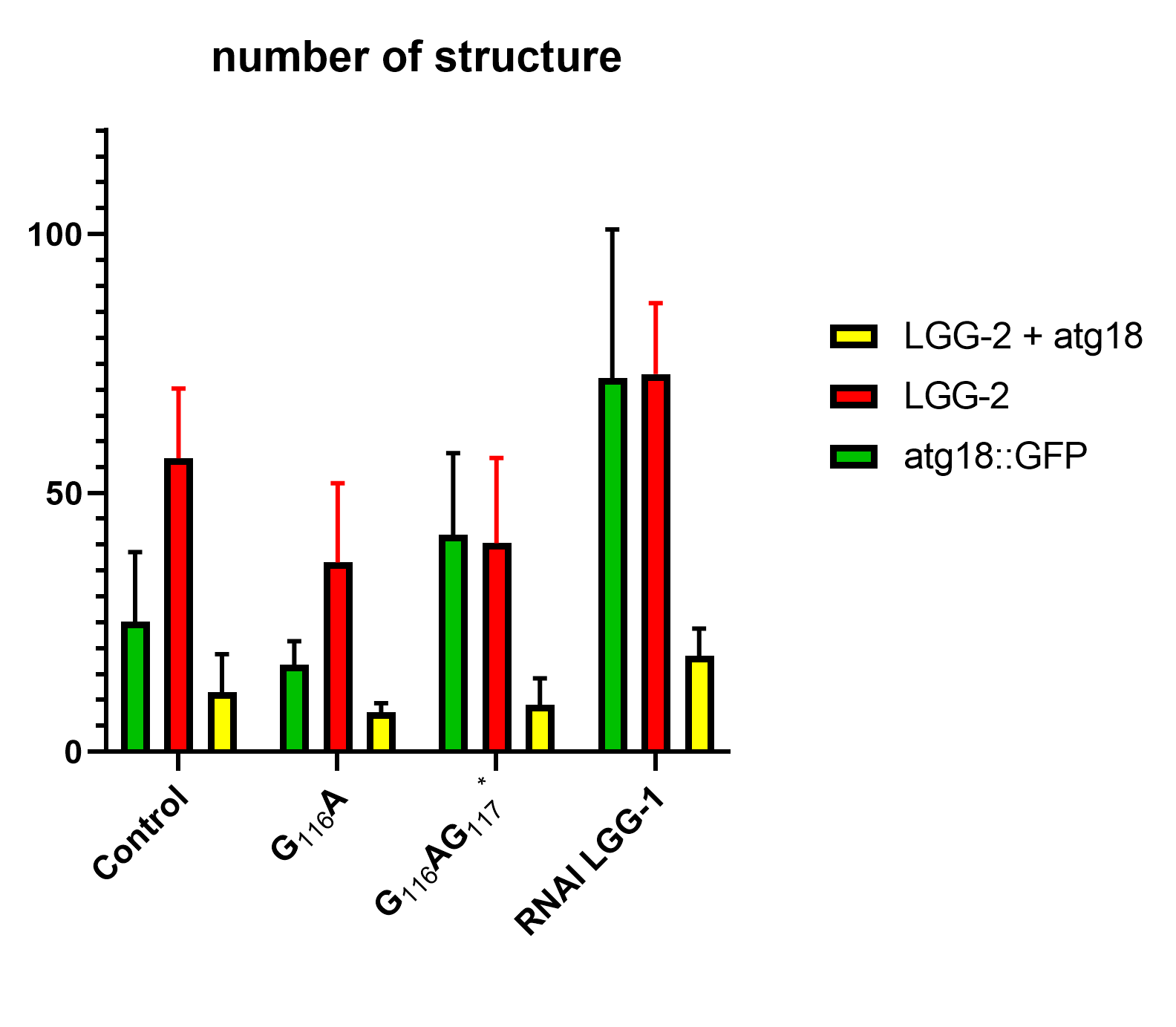

Supplement: Figure 7—source data 1. [file elife-85748-fig7-data1.zip › Figure7-Source_Data1/A-E/number of structure Atg18GFP LGG2 coloc .tif]

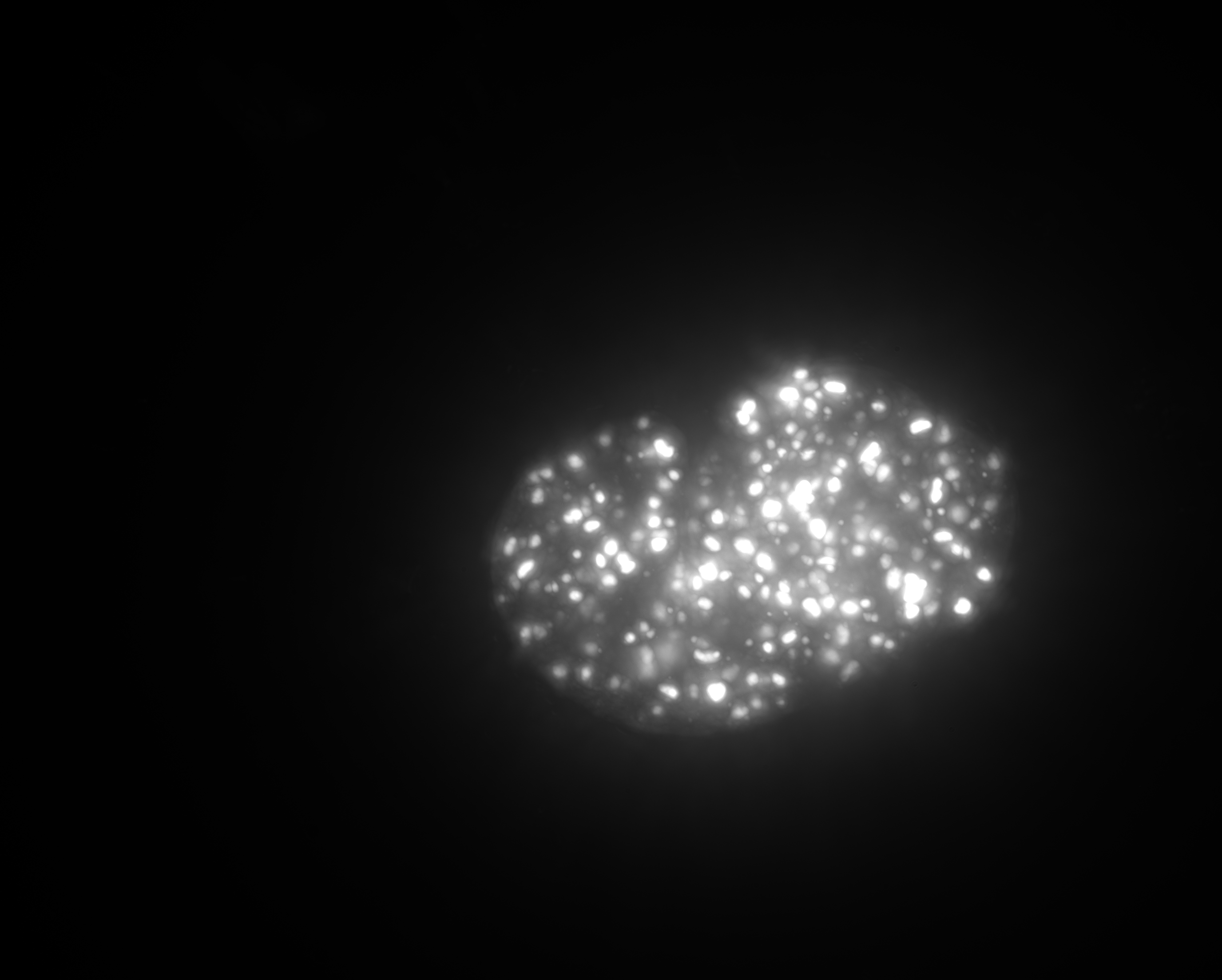

Supplement: Figure 7—source data 1. [file elife-85748-fig7-data1.zip › Figure7-Source_Data1/F-K/53gfp.tif]

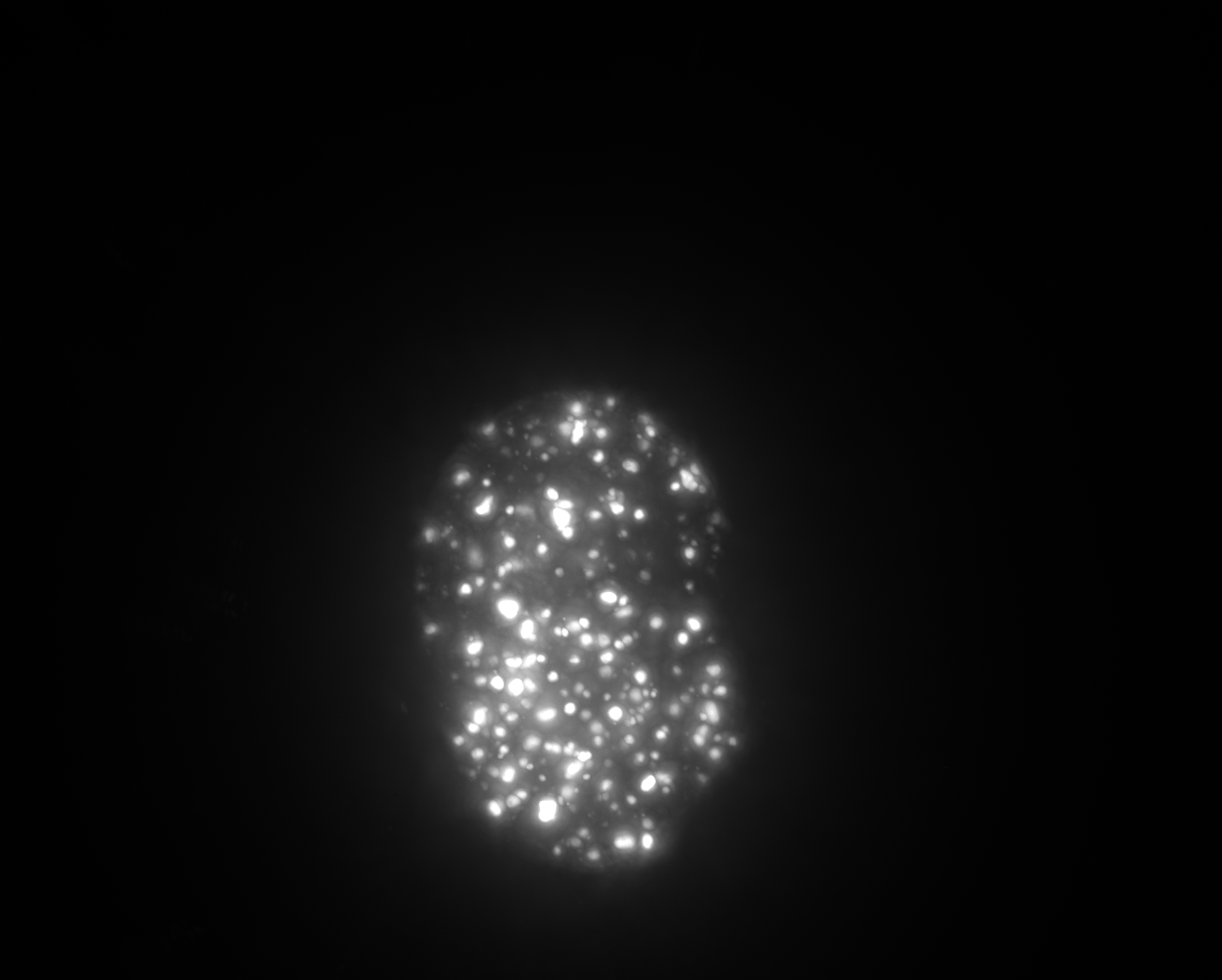

Supplement: Figure 7—source data 1. [file elife-85748-fig7-data1.zip › Figure7-Source_Data1/F-K/SEPA-gaga-L4440_gfp.tif]

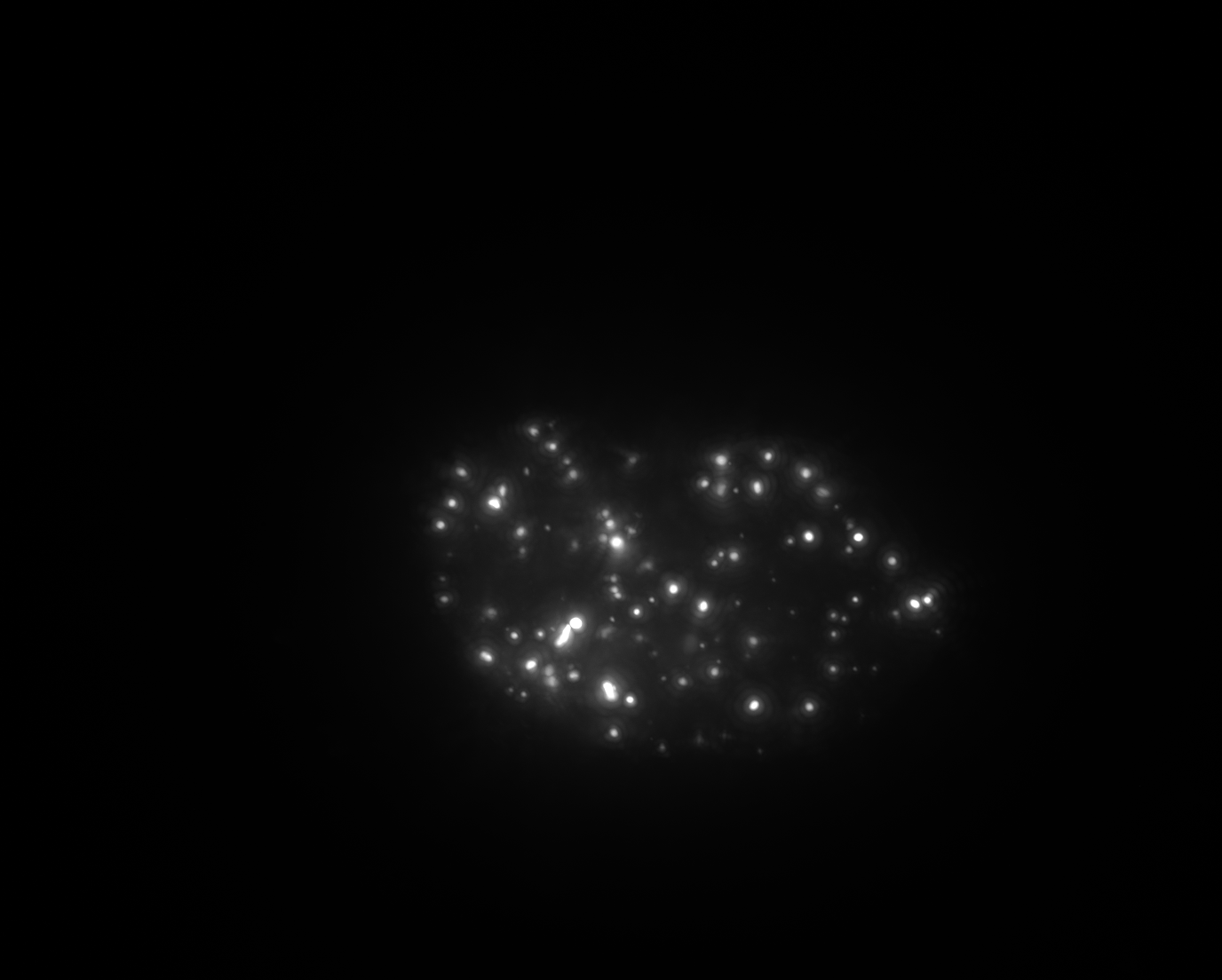

Supplement: Figure 7—source data 1. [file elife-85748-fig7-data1.zip › Figure7-Source_Data1/F-K/SEPA-ga-L4440_gfp.tif]

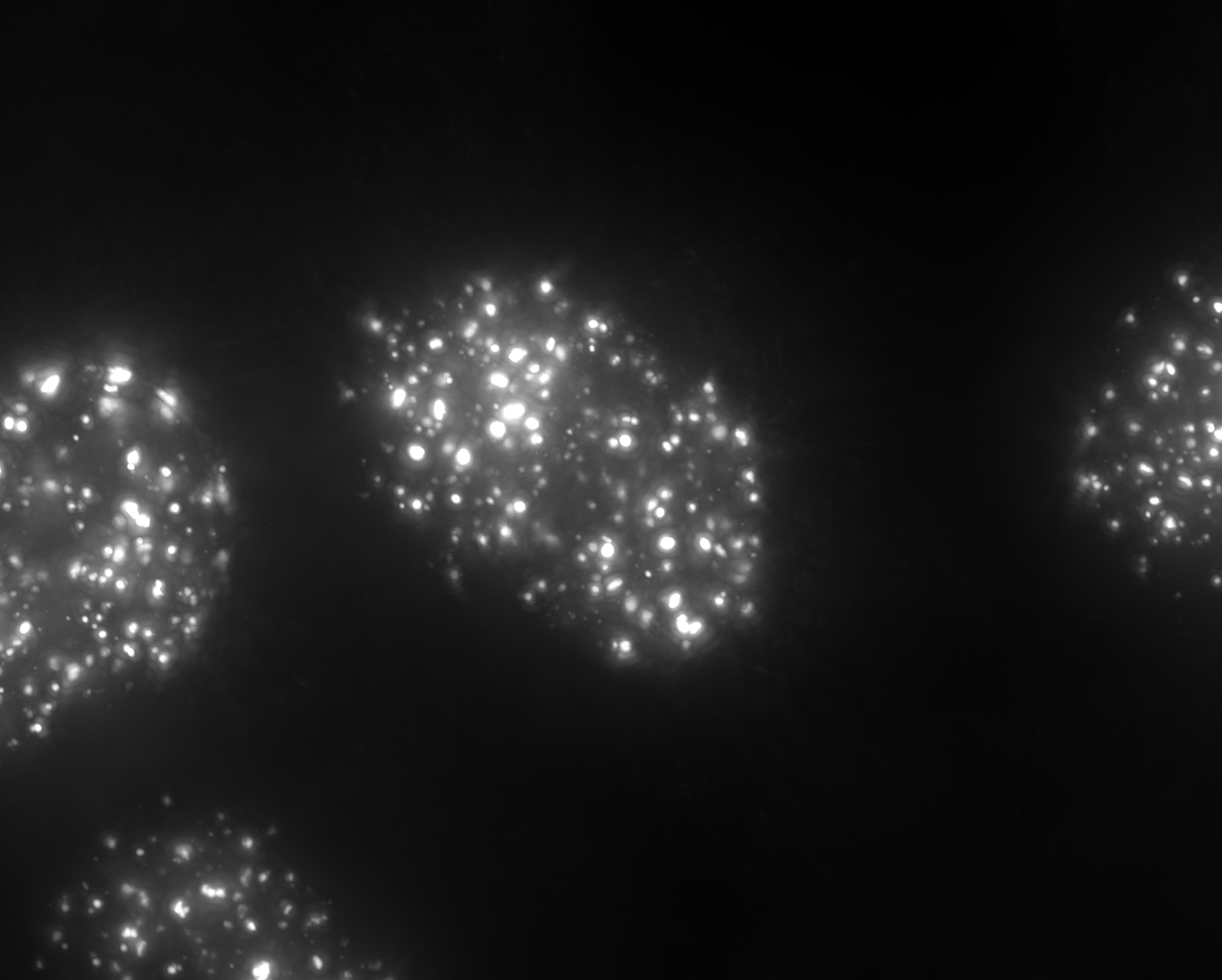

Supplement: Figure 7—source data 1. [file elife-85748-fig7-data1.zip › Figure7-Source_Data1/F-K/SEPA-gastop-L4440_gfp.tif]

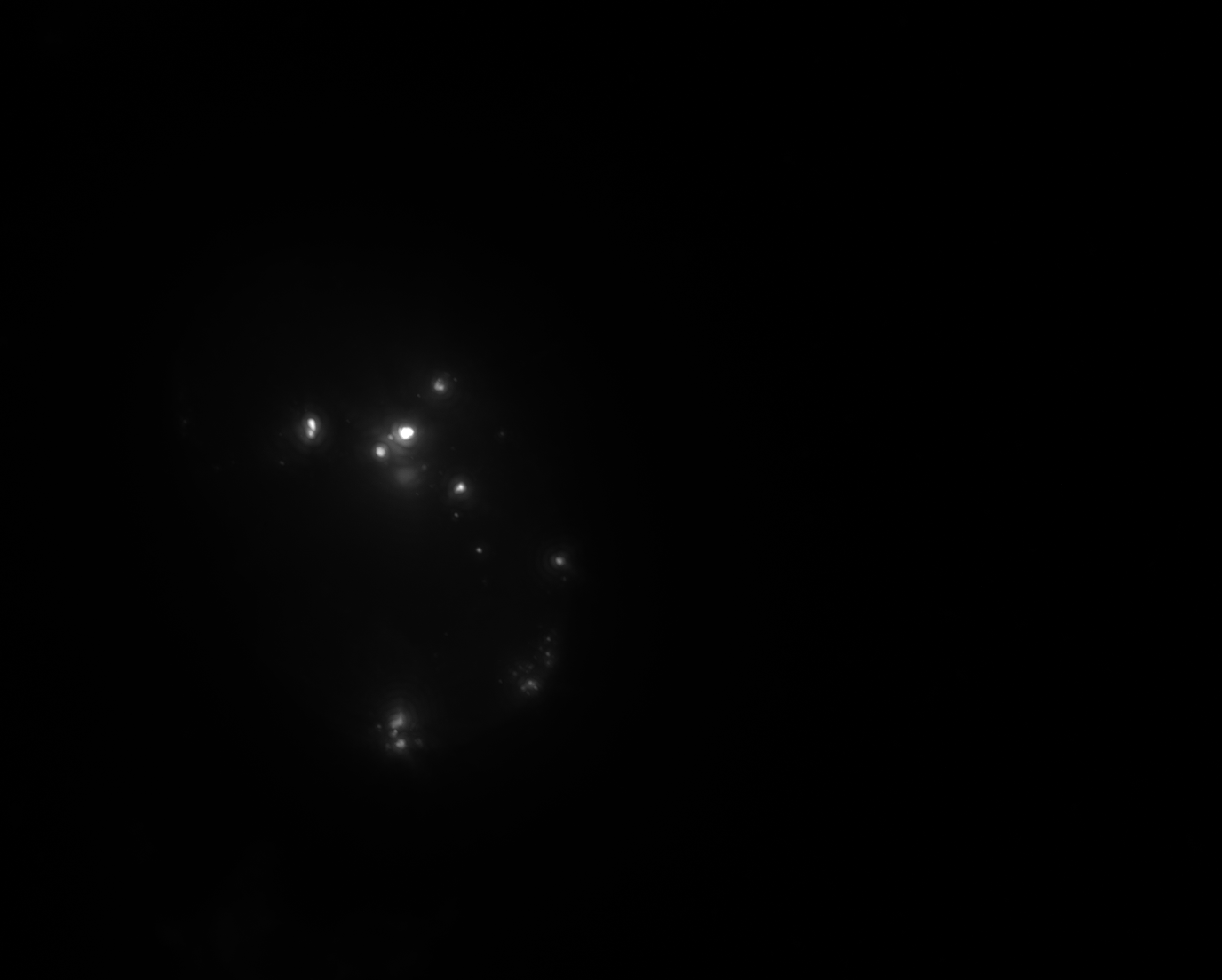

Supplement: Figure 7—source data 1. [file elife-85748-fig7-data1.zip › Figure7-Source_Data1/F-K/SEPA-wt-L4440_gfp.tif]

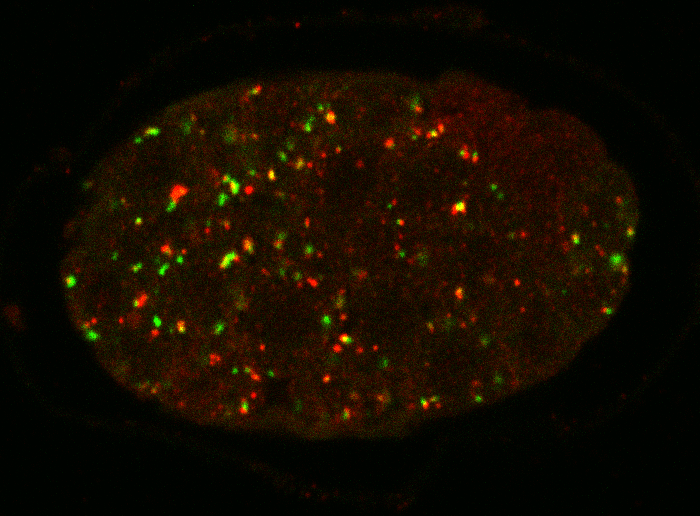

Supplement: Figure 7—source data 1. [file elife-85748-fig7-data1.zip › Figure7-Source_Data1/L-P/220919_LGG2_SEPA1GFP-N2- Series005-rot-rec-z18.tif (RGB).tif]

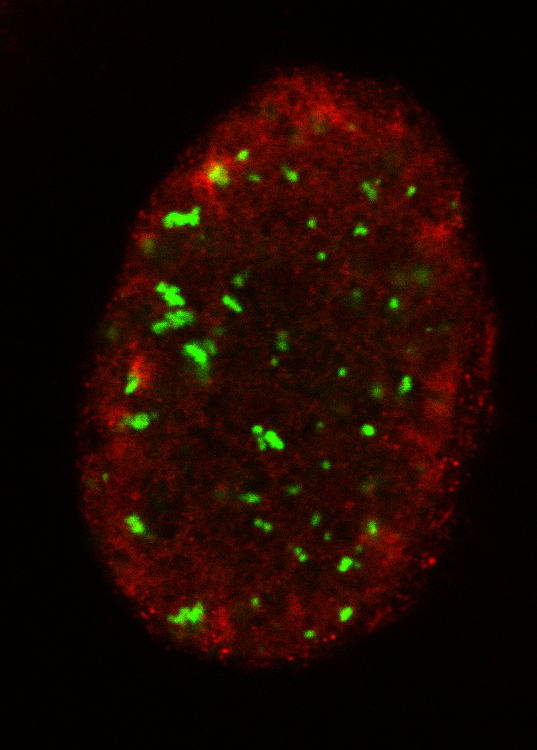

Supplement: Figure 7—source data 1. [file elife-85748-fig7-data1.zip › Figure7-Source_Data1/L-P/220930_LGG1_SEPA1GFP-GA-2-rec-z13(RGB).tif]

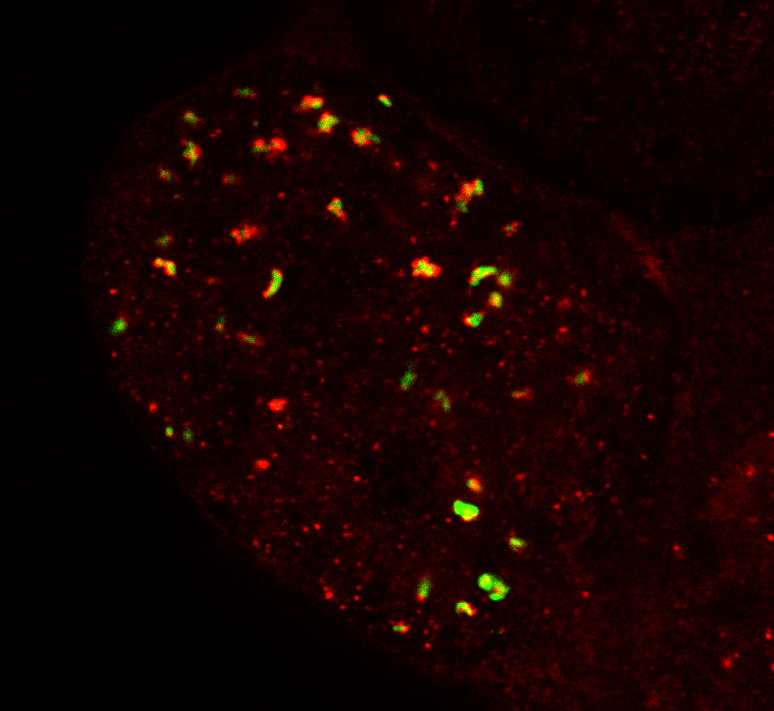

Supplement: Figure 7—source data 1. [file elife-85748-fig7-data1.zip › Figure7-Source_Data1/L-P/220930_LGG1_SEPA1GFP-N2-3-rec-z13(RGB).tif]

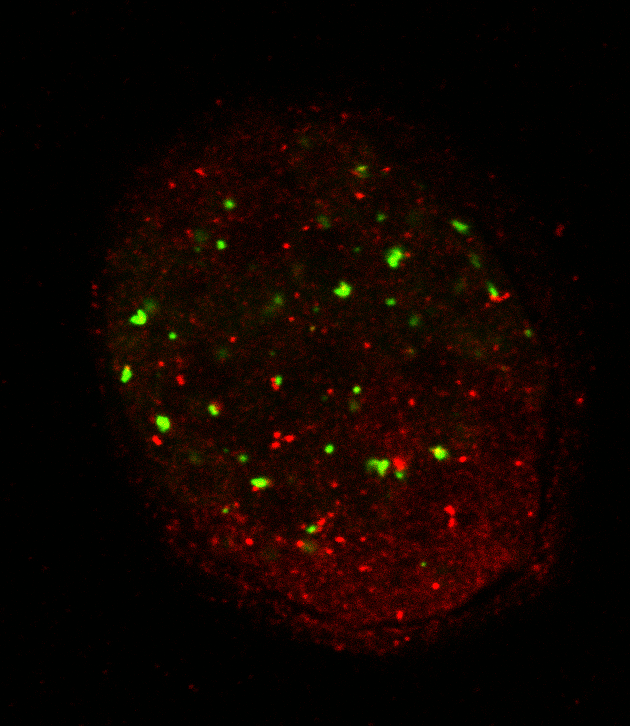

Supplement: Figure 7—source data 1. [file elife-85748-fig7-data1.zip › Figure7-Source_Data1/L-P/221007_LGG2_SEPAGFP-RD448-5-rec-z6(RGB).tif]

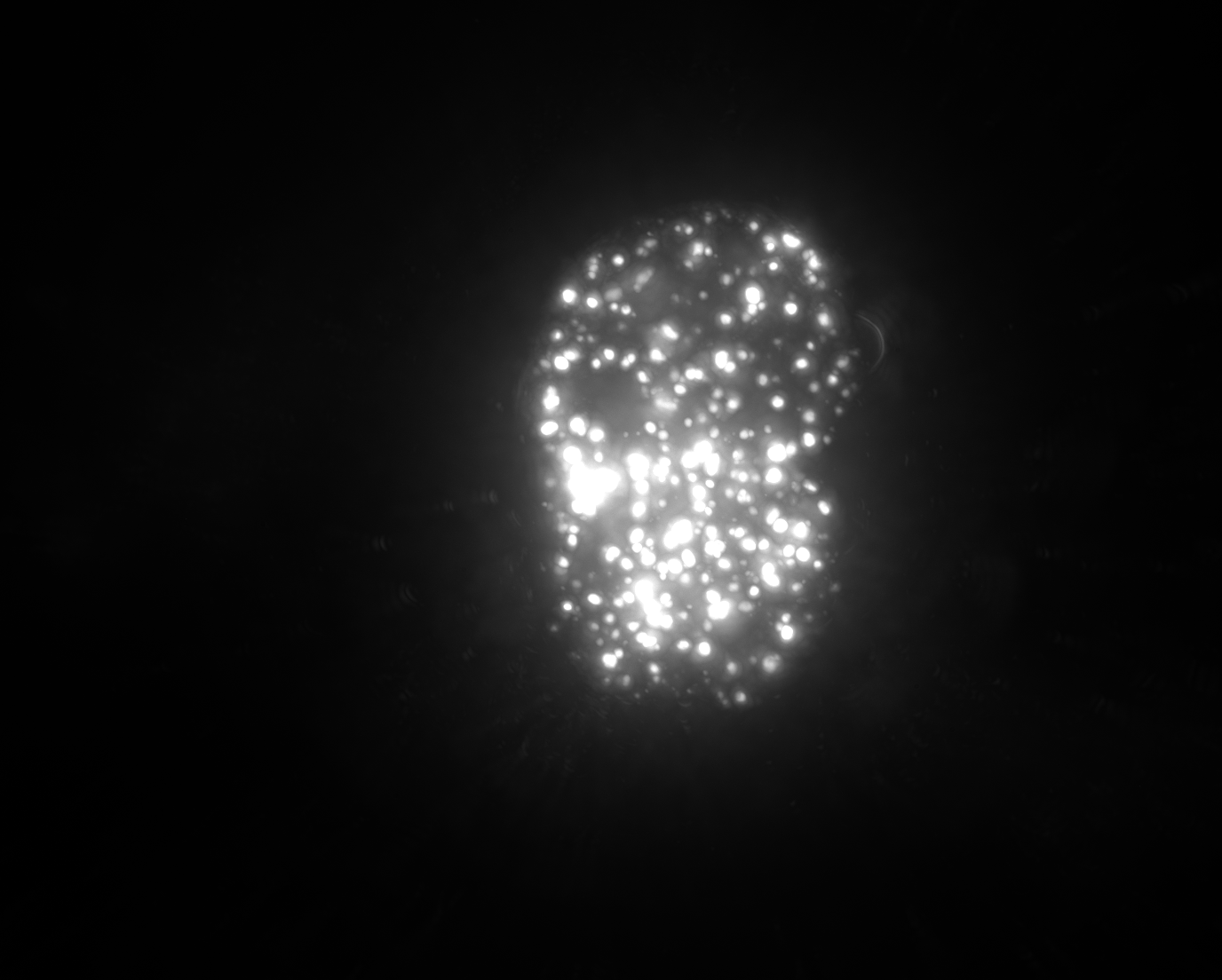

Supplement: Figure 7—figure supplement 1—source data 1. [file elife-85748-fig7-figsupp1-data1.zip › Figure7-figure-supplement1-Source_Data1/K-O/SEPA-ga-epg2_gfp.tif]

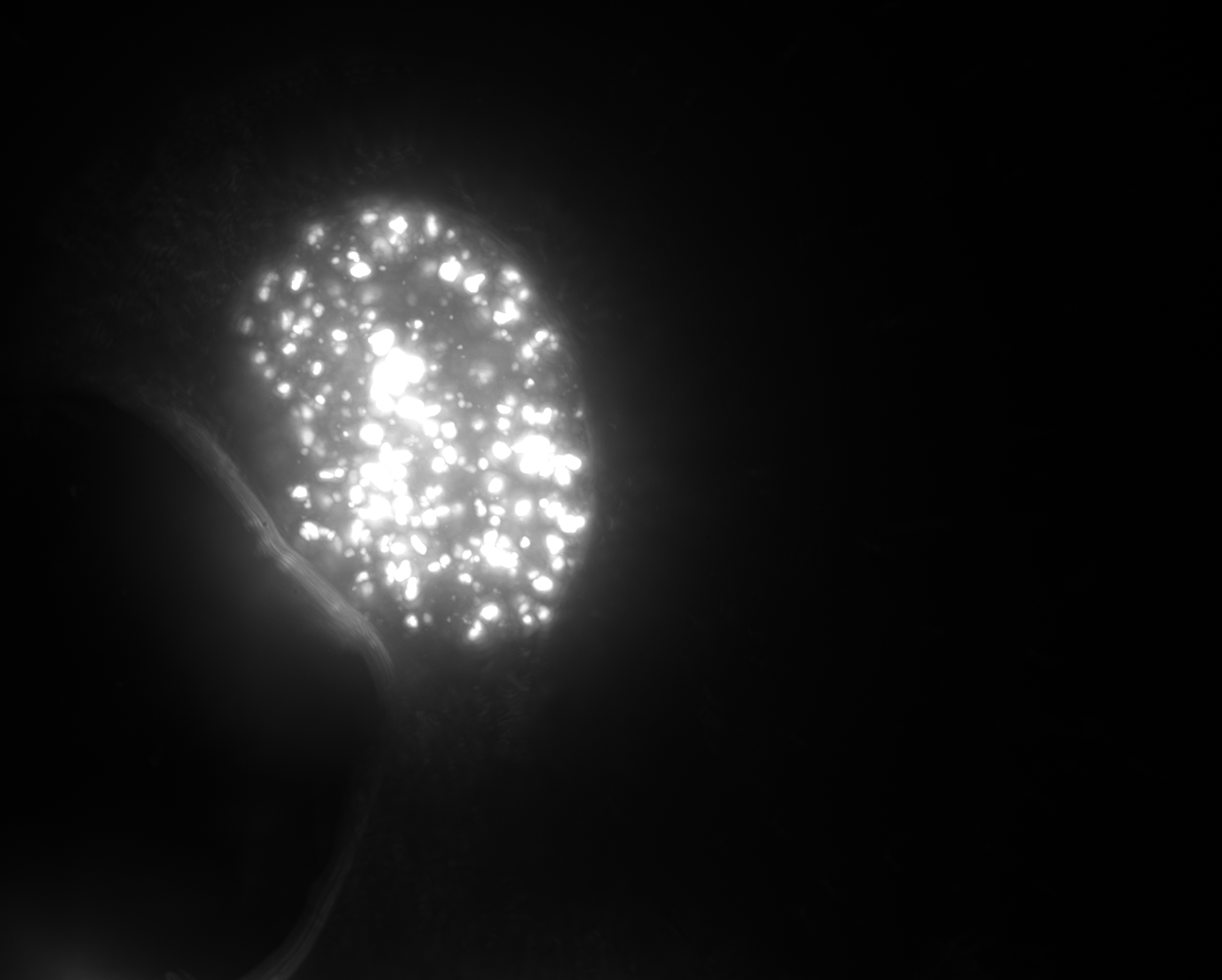

Supplement: Figure 7—figure supplement 1—source data 1. [file elife-85748-fig7-figsupp1-data1.zip › Figure7-figure-supplement1-Source_Data1/K-O/SEPA-gaga-epg2_gfp.tif]

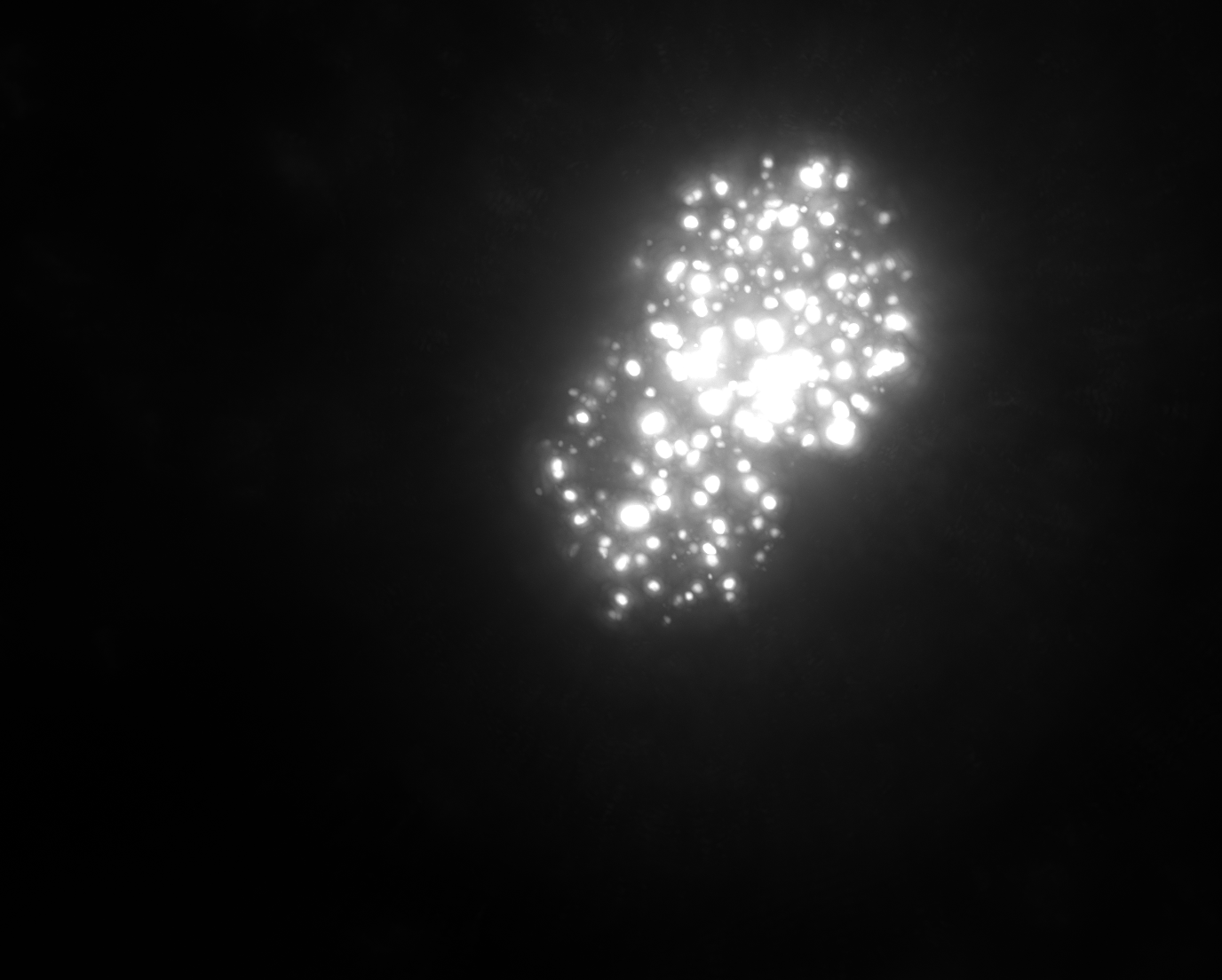

Supplement: Figure 7—figure supplement 1—source data 1. [file elife-85748-fig7-figsupp1-data1.zip › Figure7-figure-supplement1-Source_Data1/K-O/SEPA-gastop-epg2_gfp.tif]

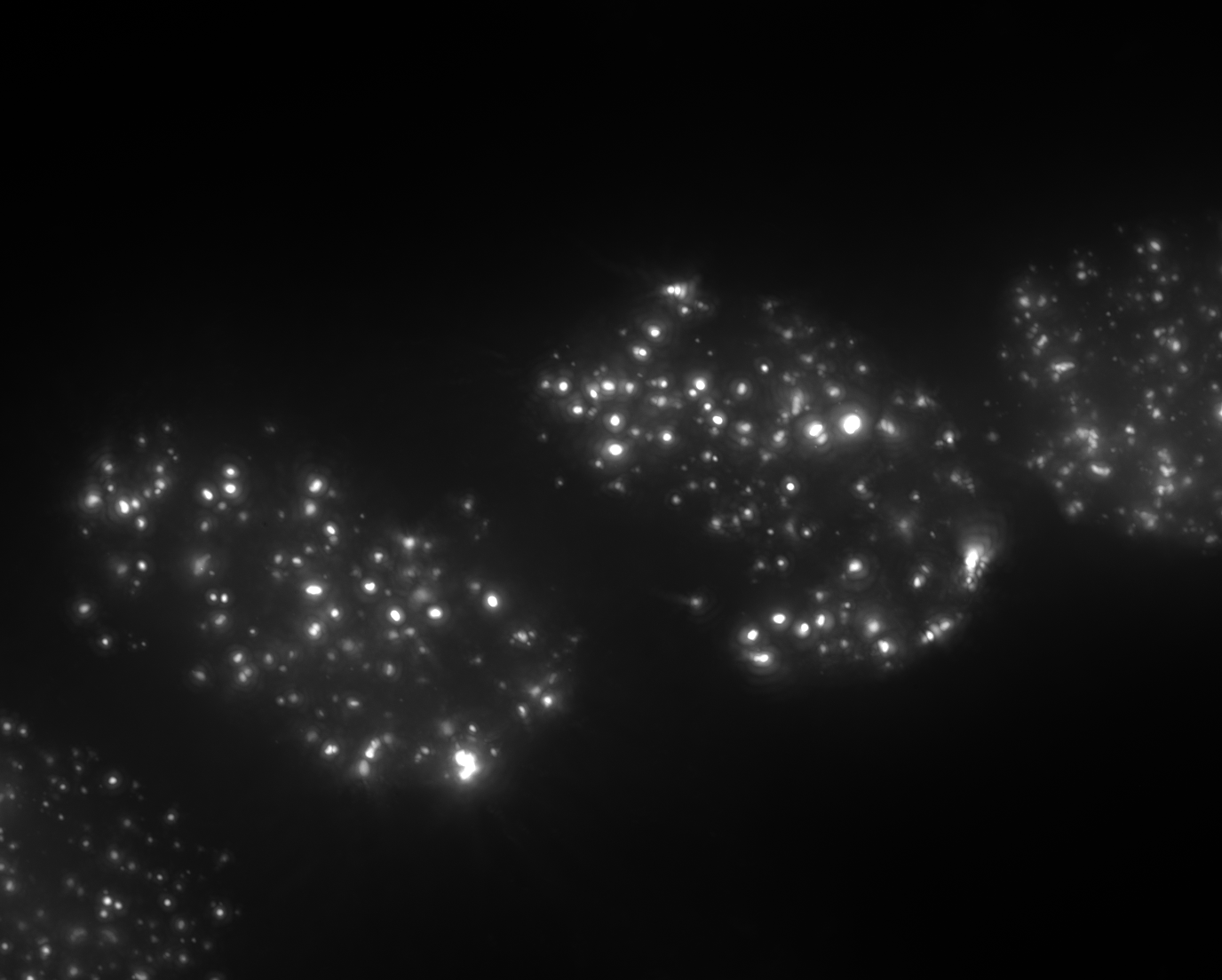

Supplement: Figure 7—figure supplement 1—source data 1. [file elife-85748-fig7-figsupp1-data1.zip › Figure7-figure-supplement1-Source_Data1/K-O/SEPA-wt-epg2_gfp.tif]
