# Supplementary material for: LGG-1/GABARAP lipidation is not required for autophagy and development in Caenorhabditis elegans
Source: eLife. 2023 Jul 3;12:e85748. doi: 10.7554/eLife.85748 (PMC10338037; doi:10.7554/eLife.85748)
Supplement: Figure 7—source data 1. [file elife-85748-fig7-data1.zip › Figure7-Source_Data1/A-E/Graph test stat manip3 number of Atg18GFP.pdf]

Table Analyzed	Copy of number of punctiform structures
Column B	*lgg-1(tm3489)*
vs.	vs,
Column A	ctrl
Mann Whitney test	
P value	<0,0001
Exact or approximate P value?	Exact
P value summary	****
Significantly different (P < 0.05)?	Yes
One- or two-tailed P value?	Two-tailed
Sum of ranks in column A,B	99 , 604
Mann-Whitney U	8

Table Analyzed	Copy of number of punctiform structures
Column C	*lgg-1(G116A)*
vs.	vs,
Column A	ctrl
Mann Whitney test	
P value	0,0060
Exact or approximate P value?	Exact
P value summary	**
Significantly different (P < 0.05)?	Yes
One- or two-tailed P value?	Two-tailed
Sum of ranks in column A,C	199 , 77
Mann-Whitney U	22

Table Analyzed	Copy of number of punctiform structures
Column D	*lgg-1(G116AG117*)*
vs.	vs,
Column A	ctrl
Mann Whitney test	
P value	<0,0001
Exact or approximate P value?	Exact
P value summary	****
Significantly different (P < 0.05)?	Yes
One- or two-tailed P value?	Two-tailed
Sum of ranks in column A,D	91 , 287
Mann-Whitney U	0

Table Analyzed	Copy of number of punctiform structures
Column E	*RNAi unc-51*
vs.	vs,
Column A	ctrl
Mann Whitney test	
P value	<0,0001
Exact or approximate P value?	Exact
P value summary	****
Significantly different (P < 0.05)?	Yes
One- or two-tailed P value?	Two-tailed
Sum of ranks in column A,E	420,5 , 440,5
Mann-Whitney U	34,50

Table Analyzed	Copy of number of punctiform structures
Column F	lgg-1(tm3489) RNAI unc-51
vs.	vs,
Column B	*lgg-1(tm3489)*
Mann Whitney test	
P value	<0,0001
Exact or approximate P value?	Exact
P value summary	****
Significantly different (P < 0.05)?	Yes
One- or two-tailed P value?	Two-tailed
Sum of ranks in column B,F	1112 , 718
Mann-Whitney U	52

Table Analyzed	Copy of number of punctiform structures
Column H	*lgg-1(G116AG117*) RNAi unc-51*
vs.	vs,
Column D	*lgg-1(G116AG117*)*
Mann Whitney test	
P value	0,7623
Exact or approximate P value?	Exact
P value summary	ns
Significantly different (P < 0.05)?	No
One- or two-tailed P value?	Two-tailed
Sum of ranks in column D,H	236 , 359
Mann-Whitney U	131

Table Analyzed	Copy of number of punctiform structures
Column G	*lgg-1(G116A) RNAi unc-51*
vs.	vs,
Column C	*lgg-1(G116A)*
Unpaired t test	
P value	0,0011
P value summary	**
Significantly different (P < 0.05)?	Yes
One- or two-tailed P value?	Two-tailed
t, df	t=3,571, df=35

Table Analyzed	Copy of number of punctiform structures
Column F	lgg-1(tm3489) RNAI unc-51
vs.	vs,
Column E	*RNAi unc-51*
Mann Whitney test	
P value	0,1939
Exact or approximate P value?	Exact
P value summary	ns
Significantly different (P < 0.05)?	No
One- or two-tailed P value?	Two-tailed
Sum of ranks in column E,F	1007 , 1074
Mann-Whitney U	407,5

Table Analyzed	Copy of number of punctiform structures
Column G vs. Column E	*lgg-1(G116A) RNAi unc-51* vs, *RNAi unc-51*
Mann Whitney test	
P value	0,1722
Exact or approximate P value?	Exact
P value summary	ns
Significantly different (P < 0.05)?	No
One- or two-tailed P value?	Two-tailed
Sum of ranks in column E,G	865,5 , 674,5
Mann-Whitney U	296,5

Table Analyzed	Copy of number of punctiform structures
Column H vs. Column E	*lgg-1(G116AG117*) RNAi unc-51* vs, *RNAi unc-51*
Mann Whitney test	
P value	<0,0001
Exact or approximate P value?	Exact
P value summary	****
Significantly different (P < 0.05)?	Yes
One- or two-tailed P value?	Two-tailed
Sum of ranks in column E,H	434 , 742
Mann-Whitney U	28

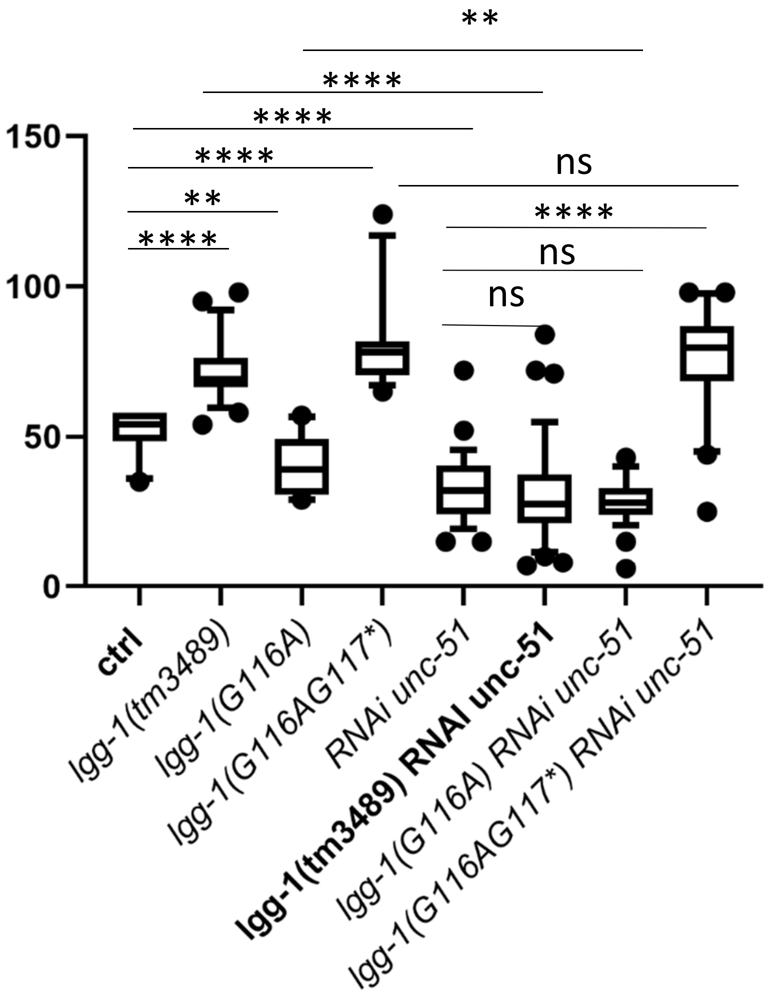

Mann Whitney
Alpha 0,05

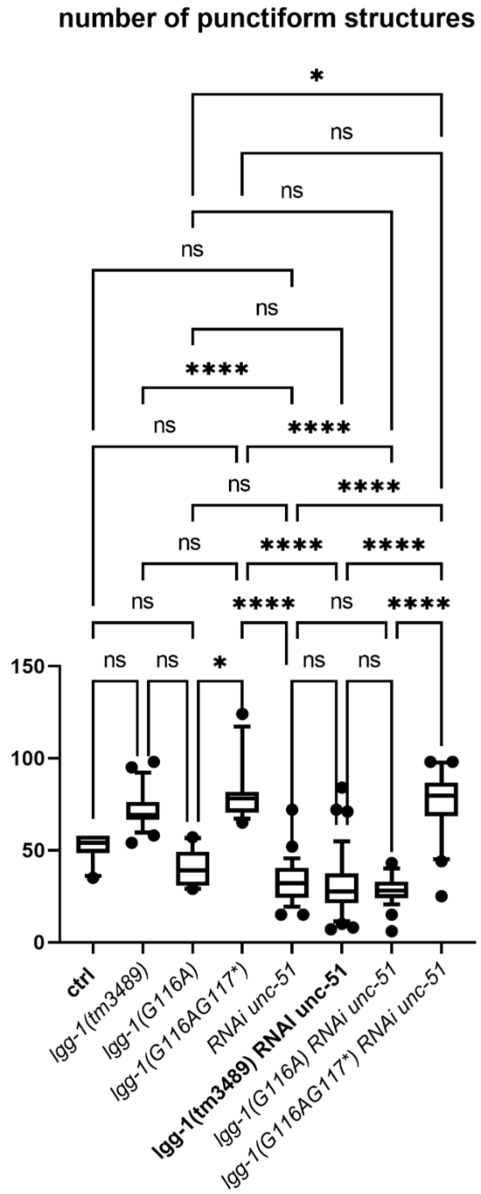

Kruskall Wallis
Alpha 0,05

Number of families	1
Number of comparisons per family	28
Alpha	0,05

Dunn's multiple comparisons test	Mean rank diff,	Significant?	Summary	Adjusted P Value	
ctrl vs. *lgg-1(tm3489)*	-32,01	No	ns	>0,9999	A-B
ctrl vs. *lgg-1(G116A)*	25,20	No	ns	>0,9999	A-C
ctrl vs. *lgg-1(G116AG117*)*	-44,69	No	ns	0,5537	A-D
ctrl vs. *RNAi unc-51*	43,15	No	ns	0,2747	A-E
ctrl vs. lgg-1(tm3489) RNAI unc-51	51,97	Yes	*	0,0351	A-F
ctrl vs. *lgg-1(G116A) RNAi unc-51*	55,14	Yes	*	0,0290	A-G
ctrl vs. *lgg-1(G116AG117*) RNAi unc-51*	-39,33	No	ns	0,7447	A-H
lgg-1(tm3489) vs. *lgg-1(G116A)*	57,20	No	ns	0,0635	B-C
lgg-1(tm3489) vs. *lgg-1(G116AG117*)*	-12,68	No	ns	>0,9999	B-D
lgg-1(tm3489) vs. *RNAi unc-51*	75,16	Yes	****	<0,0001	B-E
lgg-1(tm3489) vs. lgg-1(tm3489) RNAI unc-51	83,98	Yes	****	<0,0001	B-F
lgg-1(tm3489) vs. *lgg-1(G116A) RNAi unc-51*	87,15	Yes	****	<0,0001	B-G
lgg-1(tm3489) vs. *lgg-1(G116AG117*) RNAi unc-51*	-7,321	No	ns	>0,9999	B-H
lgg-1(G116A) vs. *lgg-1(G116AG117*)*	-69,89	Yes	*	0,0195	C-D
lgg-1(G116A) vs. *RNAi unc-51*	17,95	No	ns	>0,9999	C-E
lgg-1(G116A) vs. lgg-1(tm3489) RNAI unc-51	26,78	No	ns	>0,9999	C-F
lgg-1(G116A) vs. *lgg-1(G116A) RNAi unc-51*	29,95	No	ns	>0,9999	C-G
lgg-1(G116A) vs. *lgg-1(G116AG117*) RNAi unc-51*	-64,53	Yes	*	0,0229	C-H
lgg-1(G116AG117)* vs. *RNAi unc-51*	87,84	Yes	****	<0,0001	D-E
lgg-1(G116AG117)* vs. lgg-1(tm3489) RNAI unc-51	96,66	Yes	****	<0,0001	D-F
lgg-1(G116AG117)* vs. *lgg-1(G116A) RNAi unc-51*	99,83	Yes	****	<0,0001	D-G
lgg-1(G116AG117)* vs. *lgg-1(G116AG117*) RNAi unc-51*	5,361	No	ns	>0,9999	D-H
RNAi unc-51 vs. lgg-1(tm3489) RNAI unc-51	8,821	No	ns	>0,9999	E-F
RNAi unc-51 vs. *lgg-1(G116A) RNAi unc-51*	11,99	No	ns	>0,9999	E-G
RNAi unc-51 vs. *lgg-1(G116AG117*) RNAi unc-51*	-82,48	Yes	****	<0,0001	E-H
lgg-1(tm3489) RNAI unc-51 vs. *lgg-1(G116A) RNAi unc-51*	3,171	No	ns	>0,9999	F-G
lgg-1(tm3489) RNAI unc-51 vs. *lgg-1(G116AG117*) RNAi unc-51*	-91,30	Yes	****	<0,0001	F-H
lgg-1(G116A) RNAi unc-51 vs. *lgg-1(G116AG117*) RNAi unc-51*	-94,47	Yes	****	<0,0001	G-H

Test details	Mean rank 1	Mean rank 2	Mean rank diff,	n1	n2	Z
ctrl vs. *lgg-1(tm3489)*	101,8	133,9	-32,01	13	24	1,867
ctrl vs. *lgg-1(G116A)*	101,8	76,65	25,20	13	10	1,203
ctrl vs. *lgg-1(G116AG117*)*	101,8	146,5	-44,69	13	14	2,331
ctrl vs. *RNAi unc-51*	101,8	58,70	43,15	13	28	2,582
ctrl vs. lgg-1(tm3489) RNAI unc-51	101,8	49,88	51,97	13	36	3,226
ctrl vs. *lgg-1(G116A) RNAi unc-51*	101,8	46,70	55,14	13	27	3,281
ctrl vs. *lgg-1(G116AG117*) RNAi unc-51*	101,8	141,2	-39,33	13	20	2,217
lgg-1(tm3489) vs. *lgg-1(G116A)*	133,9	76,65	57,20	24	10	3,053
lgg-1(tm3489) vs. *lgg-1(G116AG117*)*	133,9	146,5	-12,68	24	14	0,7574
lgg-1(tm3489) vs. *RNAi unc-51*	133,9	58,70	75,16	24	28	5,427
lgg-1(tm3489) vs. lgg-1(tm3489) RNAI unc-51	133,9	49,88	83,98	24	36	6,401
lgg-1(tm3489) vs. *lgg-1(G116A) RNAi unc-51*	133,9	46,70	87,15	24	27	6,240
lgg-1(tm3489) vs. *lgg-1(G116AG117*) RNAi unc-51*	133,9	141,2	-7,321	24	20	0,4857
lgg-1(G116A) vs. *lgg-1(G116AG117*)*	76,65	146,5	-69,89	10	14	3,390
lgg-1(G116A) vs. *RNAi unc-51*	76,65	58,70	17,95	10	28	0,9789
lgg-1(G116A) vs. lgg-1(tm3489) RNAI unc-51	76,65	49,88	26,78	10	36	1,505
lgg-1(G116A) vs. *lgg-1(G116A) RNAi unc-51*	76,65	46,70	29,95	10	27	1,625
lgg-1(G116A) vs. *lgg-1(G116AG117*) RNAi unc-51*	76,65	141,2	-64,53	10	20	3,346
lgg-1(G116AG117)* vs. *RNAi unc-51*	146,5	58,70	87,84	14	28	5,390
lgg-1(G116AG117)* vs. lgg-1(tm3489) RNAI unc-51	146,5	49,88	96,66	14	36	6,164
lgg-1(G116AG117)* vs. *lgg-1(G116A) RNAi unc-51*	146,5	46,70	99,83	14	27	6,089
lgg-1(G116AG117)* vs. *lgg-1(G116AG117*) RNAi unc-51*	146,5	141,2	5,361	14	20	0,3090
RNAi unc-51 vs. lgg-1(tm3489) RNAI unc-51	58,70	49,88	8,821	28	36	0,7032
RNAi unc-51 vs. *lgg-1(G116A) RNAi unc-51*	58,70	46,70	11,99	28	27	0,8931
RNAi unc-51 vs. *lgg-1(G116AG117*) RNAi unc-51*	58,70	141,2	-82,48	28	20	5,659
lgg-1(tm3489) RNAI unc-51 vs. *lgg-1(G116A) RNAi unc-51*	49,88	46,70	3,171	36	27	0,2502
lgg-1(tm3489) RNAI unc-51 vs. *lgg-1(G116AG117*) RNAi unc-51*	49,88	141,2	-91,30	36	20	6,576
lgg-1(G116A) RNAi unc-51 vs. *lgg-1(G116AG117*) RNAi unc-51*	46,70	141,2	-94,47	27	20	6,432

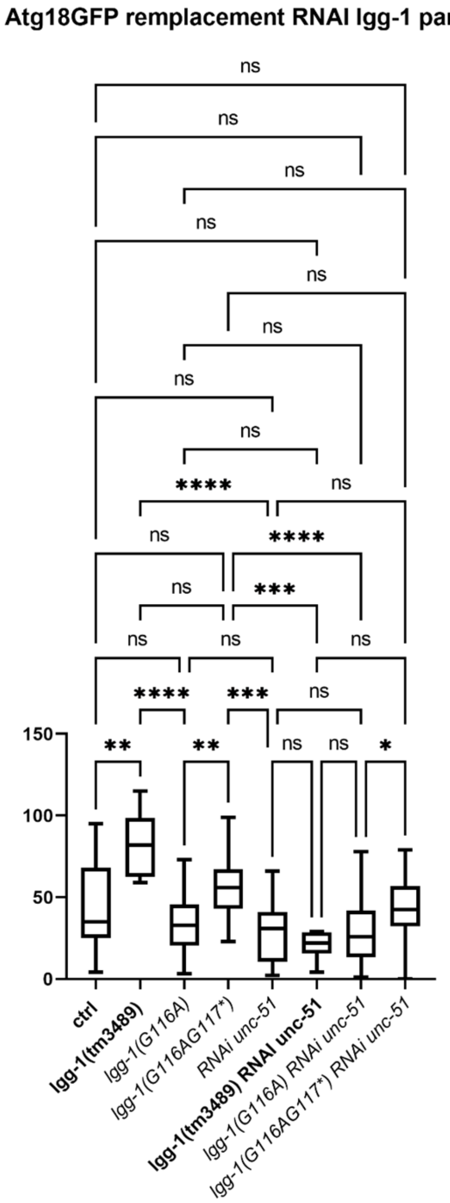

Kruskall Wallis
Alpha 0,05

Number of families	1
Number of comparisons per family	28
Alpha	0,05

Dunn's multiple comparisons test	Mean rank diff,	Significant?	Summary	Adjusted P Value	
ctrl vs. lgg-1(tm3489)	-76,69	Yes	**	0,0028	A-B
ctrl vs. lgg-1(G116A)	21,55	No	ns	>0,9999	A-C
ctrl vs. lgg-1(G116AG117*)	-37,98	No	ns	0,4517	A-D
ctrl vs. RNAi unc-51	33,43	No	ns	0,8686	A-E
ctrl vs. lgg-1(tm3489) RNAI unc-51	60,80	No	ns	0,1943	A-F
ctrl vs. lgg-1(G116A) RNAi unc-51	39,70	No	ns	0,1966	A-G
ctrl vs. lgg-1(G116AG117*) RNAi unc-51	-9,650	No	ns	>0,9999	A-H
lgg-1(tm3489) vs. lgg-1(G116A)	98,25	Yes	****	<0,0001	B-C
lgg-1(tm3489) vs. lgg-1(G116AG117*)	38,72	No	ns	>0,9999	B-D
lgg-1(tm3489) vs. RNAi unc-51	110,1	Yes	****	<0,0001	B-E
lgg-1(tm3489) vs. lgg-1(tm3489) RNAI unc-51	137,5	Yes	****	<0,0001	B-F
lgg-1(tm3489) vs. lgg-1(G116A) RNAi unc-51	116,4	Yes	****	<0,0001	B-G
lgg-1(tm3489) vs. lgg-1(G116AG117*) RNAi unc-51	67,05	Yes	*	0,0200	B-H
lgg-1(G116A) vs. lgg-1(G116AG117*)	-59,53	Yes	**	0,0028	C-D
lgg-1(G116A) vs. RNAi unc-51	11,88	No	ns	>0,9999	C-E
lgg-1(G116A) vs. lgg-1(tm3489) RNAI unc-51	39,25	No	ns	>0,9999	C-F
lgg-1(G116A) vs. lgg-1(G116A) RNAi unc-51	18,15	No	ns	>0,9999	C-G
lgg-1(G116A) vs. lgg-1(G116AG117*) RNAi unc-51	-31,20	No	ns	>0,9999	C-H
lgg-1(G116AG117*) vs. RNAi unc-51	71,41	Yes	***	0,0002	D-E
lgg-1(G116AG117*) vs. lgg-1(tm3489) RNAI unc-51	98,78	Yes	***	0,0004	D-F
lgg-1(G116AG117*) vs. lgg-1(G116A) RNAi unc-51	77,68	Yes	****	<0,0001	D-G
lgg-1(G116AG117*) vs. lgg-1(G116AG117*) RNAi unc-51	28,33	No	ns	>0,9999	D-H
RNAi unc-51 vs. lgg-1(tm3489) RNAI unc-51	27,37	No	ns	>0,9999	E-F
RNAi unc-51 vs. lgg-1(G116A) RNAi unc-51	6,273	No	ns	>0,9999	E-G
RNAi unc-51 vs. lgg-1(G116AG117*) RNAi unc-51	-43,08	No	ns	0,1644	E-H
lgg-1(tm3489) RNAI unc-51 vs. lgg-1(G116A) RNAi unc-51	-21,10	No	ns	>0,9999	F-G
lgg-1(tm3489) RNAI unc-51 vs. lgg-1(G116AG117*) RNAi unc-51	-70,45	No	ns	0,0515	F-H
lgg-1(G116A) RNAi unc-51 vs. lgg-1(G116AG117*) RNAi unc-51	-49,35	Yes	*	0,0253	G-H

Test details	Mean rank 1	Mean rank 2	Mean rank diff,	n1	n2	Z
ctrl vs. lgg-1(tm3489)	109,2	185,9	-76,69	29	13	3,893
ctrl vs. lgg-1(G116A)	109,2	87,64	21,55	29	33	1,435
ctrl vs. lgg-1(G116AG117*)	109,2	147,2	-37,98	29	27	2,406
ctrl vs. RNAi unc-51	109,2	75,76	33,43	29	29	2,157
ctrl vs. lgg-1(tm3489) RNAI unc-51	109,2	48,39	60,80	29	9	2,700
ctrl vs. lgg-1(G116A) RNAi unc-51	109,2	69,49	39,70	29	36	2,696
ctrl vs. lgg-1(G116AG117*) RNAi unc-51	109,2	118,8	-9,650	29	28	0,6171
lgg-1(tm3489) vs. lgg-1(G116A)	185,9	87,64	98,25	13	33	5,083
lgg-1(tm3489) vs. lgg-1(G116AG117*)	185,9	147,2	38,72	13	27	1,943
lgg-1(tm3489) vs. RNAi unc-51	185,9	75,76	110,1	13	29	5,590
lgg-1(tm3489) vs. lgg-1(tm3489) RNAI unc-51	185,9	48,39	137,5	13	9	5,372
lgg-1(tm3489) vs. lgg-1(G116A) RNAi unc-51	185,9	69,49	116,4	13	36	6,095
lgg-1(tm3489) vs. lgg-1(G116AG117*) RNAi unc-51	185,9	118,8	67,05	13	28	3,385
lgg-1(G116A) vs. lgg-1(G116AG117*)	87,64	147,2	-59,53	33	27	3,887
lgg-1(G116A) vs. RNAi unc-51	87,64	75,76	11,88	33	29	0,7906
lgg-1(G116A) vs. lgg-1(tm3489) RNAI unc-51	87,64	48,39	39,25	33	9	1,768
lgg-1(G116A) vs. lgg-1(G116A) RNAi unc-51	87,64	69,49	18,15	33	36	1,276
lgg-1(G116A) vs. lgg-1(G116AG117*) RNAi unc-51	87,64	118,8	-31,20	33	28	2,058
lgg-1(G116AG117*) vs. RNAi unc-51	147,2	75,76	71,41	27	29	4,524
lgg-1(G116AG117*) vs. lgg-1(tm3489) RNAI unc-51	147,2	48,39	98,78	27	9	4,348
lgg-1(G116AG117*) vs. lgg-1(G116A) RNAi unc-51	147,2	69,49	77,68	27	36	5,170
lgg-1(G116AG117*) vs. lgg-1(G116AG117*) RNAi unc-51	147,2	118,8	28,33	27	28	1,779
RNAi unc-51 vs. lgg-1(tm3489) RNAI unc-51	75,76	48,39	27,37	29	9	1,215
RNAi unc-51 vs. lgg-1(G116A) RNAi unc-51	75,76	69,49	6,273	29	36	0,4259
RNAi unc-51 vs. lgg-1(G116AG117*) RNAi unc-51	75,76	118,8	-43,08	29	28	2,755
lgg-1(tm3489) RNAI unc-51 vs. lgg-1(G116A) RNAi unc-51	48,39	69,49	-21,10	9	36	0,9591
lgg-1(tm3489) RNAI unc-51 vs. lgg-1(G116AG117*) RNAi unc-51	48,39	118,8	-70,45	9	28	3,115
lgg-1(G116A) RNAi unc-51 vs. lgg-1(G116AG117*) RNAi unc-51	69,49	118,8	-49,35	36	28	3,318
