# Supplementary material for: LGG-1/GABARAP lipidation is not required for autophagy and development in Caenorhabditis elegans
Source: eLife. 2023 Jul 3;12:e85748. doi: 10.7554/eLife.85748 (PMC10338037; doi:10.7554/eLife.85748)
Supplement: Figure 7—source data 1. [file elife-85748-fig7-data1.zip › Figure7-Source_Data1/F-K/Tests stat quantif SEPA novembre 2022 avec lgg-1(tm3489).pdf]

Quantification nombre de points

croisements mutants lgg-1 avec SEAP-1 ::GFP sur <u>RNAi l4440 et epg-2</u>

GAL2=c(100,108,95,138,109,92,187,202,166)
GA2=c(269,263,224,211,207,197,244,262,231,292)
GASTOPL2=c(271,321,274,275,298,214,248,229,262,282)
GASTOP2=c(282,230,284,213,247,244,245,218,246,178)
GAGAL2=c(272,259,262,265,241,274,225,241,247,298)
GAGA2=c(286,262,248,248,290,248,240,247,278,238)
SEPAL2=c(21,6,1,54,61,8,26,2,4,11)
SEPA2=c(286,296,271,221,156,136,129,172,126,196)
LGG1=c(283,245,266,314,281,282,284,256,270,276)

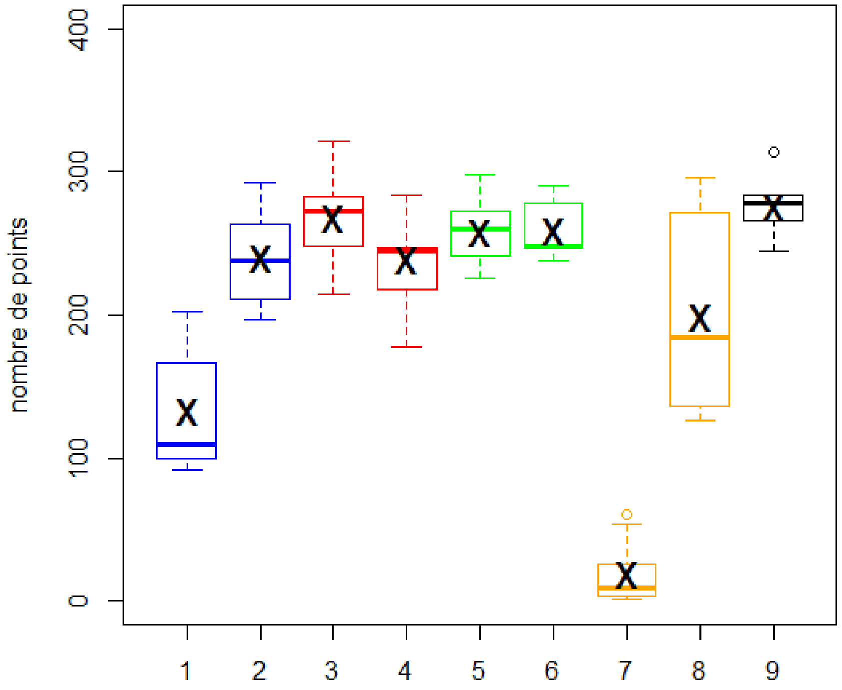

boxplot(list(GAL2,GA2,GASTOPL2,GASTOP2,GAGAL2,GAGA2,SEPAL2,SEPA2,LGG1), method="jitter", vertical=T, ylim=c(0,400), ylab="nombre de points",border=c("blue","blue","red","red","green","green","orange","orange","black"))
points(c(1,2,3,4,5,6,7,8,9),c(mean(GAL2), mean(GA2), mean(GASTOPL2), mean(GASTOP2),mean(GAGAL2),mean(GAGA2),mean(SEPAL2),mean(SEPA2), mean (LGG1)),pch="x",cex=2,col=c("black"))

* inf. 0,05; ** inf. 0,01; *** inf. 0,001; **** inf. 0,0001

wilcox.test(GAL2, GA2) p-value = 4.33e-05 ****
wilcox.test(GASTOPL2, GASTOP2) p-value = 0.08198 n.s.
wilcox.test(GAGAL2, GAGA2) p-value = 1 n.s.
wilcox.test(SEPAL2, SEPA2) p-value = 1.083e-05 ****
wilcox.test(SEPAL2, LGG1) p-value = 1.083e-05 ****

wilcox.test(GAL2, GASTOPL2) p-value = 2.165e-05 ****
wilcox.test(GAL2, GAGAL2) p-value = 0.000278 ***
wilcox.test(GAL2, SEPAL2) p-value = 2.165e-05 ****
wilcox.test(GAL2, LGG1) p-value = 2.165e-05 ****

wilcox.test(GASTOPL2, GAGAL2) p-value = 0.325 n.s.
wilcox.test(GASTOPL2, SEPAL2) p-value = 1.083e-05 ****
wilcox.test(GASTOPL2, LGG1) p-value = 0.4961 n.s.

wilcox.test(GAGAL2, SEPAL2) p-value = 0.0001817 ***
wilcox.test(GAGAL2, LGG1) p-value = 0.05381 n.s.

wilcox.test(GA2, GASTOP2) p-value = 0.9397 n.s.
wilcox.test(GA2, GAGA2) p-value = 0.1979 n.s.
wilcox.test(GA2, SEPA2) p-value = 0.1655 n.s.
wilcox.test(GA2, LGG1) p-value = 0.008931 **

wilcox.test(GASTOP2, GAGA2) p-value = 0.05831 n.s.
wilcox.test(GASTOP2, SEPA2) p-value = 0.1903 n.s.
wilcox.test(GASTOP2, LGG1) p-value = 0.01545 *

wilcox.test(GAGA2, SEPA2) p-value = 0.06912 n.s.
wilcox.test(GAGA2, LGG1) p-value = 0.1207 n.s.

wilcox.test(SEPA2, LGG1) p-value = 0.03546 *

Quantification intensité moyenne de l'embryon

croisements mutants lgg-1 avec SEAP-1 ::GFP sur <u>RNAi l4440 et epg-2</u>

GAL2=c(2300,2652,1852,3067,1960,1720,4312,4416,3477)
GA2=c(3630,3263,2068,1795,9397,6360,9913,5959,6115,7135)
GASTOPL2=c(4828,6641,8430,6041,7469,5110,4555,5600,7192,4968)
GASTOP2=c(2843,1786,2375,2392,7360,6051,5757,5359,8367,10338)
GAGAL2=c(5567,5301,6691,7585,8477,6151,6548,5854,6488,7184)
GAGA2=c(8488,7015,6883,8355,7520,5034,6548,6945,9130,8623)
SEPAL2=c(890,420,337,952,1136,398,734,548,707,934)
SEPA2=c(2928,3854,2704,2185,3048,2615,3546,1797,2410,2055)
LGG1=c(5575,4928,9781,8330,6089,5501,7079,6726,6343,8394)

boxplot(list(GAL2,GA2,GASTOPL2,GASTOP2,GAGAL2,GAGA2,SEPAL2,SEPA2,LGG1), method="jitter", vertical=T, ylim=c(0,11000), ylab="nombre de points",border=c("blue","blue","red","red","green","green","orange","orange","black"))
points(c(1,2,3,4,5,6,7,8,9),c(mean(GAL2), mean(GA2), mean(GASTOPL2), mean(GASTOP2),mean(GAGAL2),mean(GAGA2),mean(SEPAL2),mean(SEPA2), mean (LGG1)),pch="x",cex=2,col=c("black"))

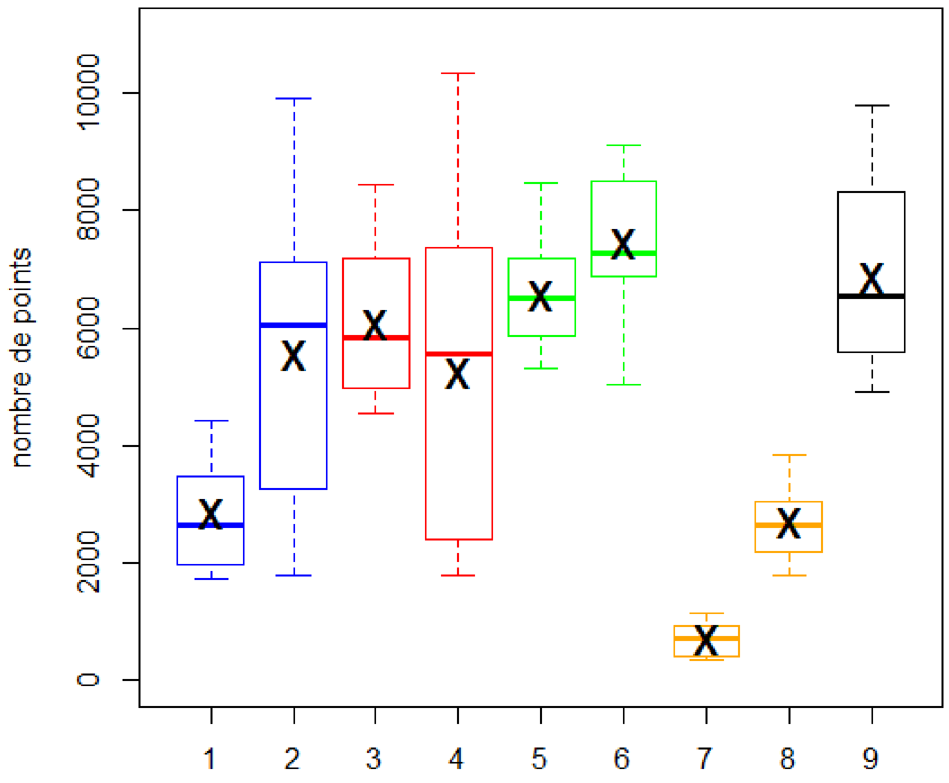

* inf. 0,05; ** inf. 0,01; *** inf. 0,001; **** inf. 0,0001

wilcox.test(GAL2, GA2) p-value = 0.03499 *
wilcox.test(GASTOPL2, GASTOP2) p-value = 0.5787 n.s.
wilcox.test(GAGAL2, GAGA2) p-value = 0.08198 n.s.
wilcox.test(SEPAL2, SEPA2) p-value = 1.083e-05 ****
wilcox.test(SEPAL2, LGG1) p-value = 1.083e-05 ****

wilcox.test(GAL2, GASTOPL2) p-value = 2.165e-05 ****
wilcox.test(GAL2, GAGAL2) p-value = 2.165e-05 ****
wilcox.test(GAL2, SEPAL2) p-value = 2.165e-05 ****
wilcox.test(GAL2, LGG1) p-value = 2.165e-05 ****

wilcox.test(GASTOPL2, GAGAL2) p-value = 0.315 n.s.
wilcox.test(GASTOPL2, SEPAL2) p-value = 1.083e-05 ****
wilcox.test(GASTOPL2, LGG1) p-value = 0.315 n.s.

wilcox.test(GAGAL2, SEPAL2) p-value = 1.083e-05 ****
wilcox.test(GAGAL2, LGG1) p-value = 0.9118 n.s.

wilcox.test(GA2, GASTOP2) p-value = 0.7394 n.s.
wilcox.test(GA2, GAGA2) p-value = 0.1051 n.s.
wilcox.test(GA2, SEPA2) p-value = 0.02881*
wilcox.test(GA2, LGG1) p-value = 0.393 n.s.

wilcox.test(GASTOP2, GAGA2) p-value = 0.06301 n.s.
wilcox.test(GASTOP2, SEPA2) p-value = 0.1051 n.s.
wilcox.test(GASTOP2, LGG1) p-value = 0.1903 n.s.

wilcox.test(GAGA2, SEPA2) p-value = 1.083e-05 ****
wilcox.test(GAGA2, LGG1) p-value = 0.2475 n.s.

wilcox.test(SEPA2, LGG1) p-value = 1.083e-05 ****
