# Supplementary material for: LGG-1/GABARAP lipidation is not required for autophagy and development in Caenorhabditis elegans
Source: eLife. 2023 Jul 3;12:e85748. doi: 10.7554/eLife.85748 (PMC10338037; doi:10.7554/eLife.85748)
Supplement: Figure 7—source data 1. [file elife-85748-fig7-data1.zip › Figure7-Source_Data1/L-P/Colocalisation-Jacop.pdf]

CONTROLES *lgg-1(wt)* CO-LOCALISATION SEPA-1::GFP et LGG-2

****
Image A: C1-221007_LGG2_SEPAGFP-HZ455-1.lif
Image B: C2-221007_LGG2_SEPAGFP-HZ455-1.lif

Pearson's Coefficient:
r=0.497

Overlap Coefficient:
r=0.726

r^2=k1xk2:
k1=1.58
k2=0.334

Using thresholds (thrA=65 and thrB=114)

Overlap Coefficient:
r=0.906

r^2=k1xk2:
k1=1.255
k2=0.655

Manders' Coefficients (original):
M1=0.999 (fraction of A overlapping B)
M2=1.0 (fraction of B overlapping A)

Manders' Coefficients (using threshold value of 65 for imgA and 114 for imgB):
M1=0.153 (fraction of A overlapping B)
M2=0.172 (fraction of B overlapping A)

Costes' automatic threshold set to 4 for imgA & 15 for imgB
Pearson's Coefficient:
r=0.261 (0.0 below thresholds)
M1=0.879 & M2=0.858

****
Image A: C1-221007_LGG2_SEPAGFP-HZ455-2.lif - Series001-1
Image B: C2-221007_LGG2_SEPAGFP-HZ455-2.lif - Series001-1

Pearson's Coefficient:
r=0.36

Overlap Coefficient:
r=0.618

r^2=k1xk2:
k1=1.598
k2=0.238

****
Image A: C1-221007_LGG2_SEPAGFP-HZ455-2.lif - Series002-1
Image B: C2-221007_LGG2_SEPAGFP-HZ455-2.lif - Series002-1

Pearson's Coefficient:
r=0.405

Overlap Coefficient:
r=0.655

r^2=k1xk2:
k1=0.998
k2=0.43

Using thresholds (thrA=108 and thrB=89)

Overlap Coefficient:
r=0.943

r^2=k1xk2:
k1=0.572
k2=1.557

Manders' Coefficients (original):
M1=0.999 (fraction of A overlapping B)
M2=1.0 (fraction of B overlapping A)

Manders' Coefficients (using threshold value of 108 for imgA and 89 for imgB):
M1=0.223 (fraction of A overlapping B)
M2=0.176 (fraction of B overlapping A)

Costes' automatic threshold set to 3 for imgA & 18 for imgB
Pearson's Coefficient:
r=0.296 (0.0 below thresholds)
M1=0.943 & M2=0.8
Using thresholds (thrA=73 and thrB=121)

Overlap Coefficient:
r=0.953

r^2=k1xk2:
k1=0.587
k2=1.548

Manders' Coefficients (original):
M1=1.0 (fraction of A overlapping B)
M2=0.999 (fraction of B overlapping A)

Manders' Coefficients (using threshold value of 73 for imgA and 121 for imgB):
M1=0.034 (fraction of A overlapping B)

M2=0.044 (fraction of B overlapping A)

Costes' automatic threshold set to 3 for imgA & 25 for imgB
Pearson's Coefficient:
r=0.064 (0.0 below thresholds)
M1=0.901 & M2=0.901
****
Image A: C1-221007_LGG2_SEPAGFP-HZ455-2.lif - Series003-1
Image B: C2-221007_LGG2_SEPAGFP-HZ455-2.lif - Series003-1

Pearson's Coefficient:
r=0.446

Overlap Coefficient:
r=0.673

r^2=k1xk2:
k1=1.701
k2=0.266

Using thresholds (thrA=71 and thrB=117)

Overlap Coefficient:
r=0.928

r^2=k1xk2:
k1=0.75
k2=1.147

Manders' Coefficients (original):
M1=1.0 (fraction of A overlapping B)
M2=1.0 (fraction of B overlapping A)

Manders' Coefficients (using threshold value of 71 for imgA and 117 for imgB):
M1=0.285 (fraction of A overlapping B)
M2=0.142 (fraction of B overlapping A)

Costes' automatic threshold set to 3 for imgA & 25 for imgB
Pearson's Coefficient:
r=0.241 (0.0 below thresholds)
M1=0.919 & M2=0.911

****
Image A: C1-221007_LGG2_SEPAGFP-HZ455-2.lif - Series004-1
Image B: C2-221007_LGG2_SEPAGFP-HZ455-2.lif - Series004-1

Pearson's Coefficient:
r=0.391

Overlap Coefficient:
r=0.6

r^2=k1xk2:
k1=1.112
k2=0.324

Using thresholds (thrA=83 and thrB=99)

Overlap Coefficient:
r=0.945

r^2=k1xk2:
k1=0.589
k2=1.515

Manders' Coefficients (original):
M1=1.0 (fraction of A overlapping B)
M2=1.0 (fraction of B overlapping A)

Manders' Coefficients (using threshold value of 83 for imgA and 99 for imgB):
M1=0.244 (fraction of A overlapping B)
M2=0.507 (fraction of B overlapping A)

Costes' automatic threshold set to 4 for imgA & 22 for imgB
Pearson's Coefficient:
r=0.324 (0.0 below thresholds)
M1=0.871 & M2=0.843

****
Image A: C1-221007_LGG2_SEPAGFP-HZ455-2.lif - Series005-1
Image B: C2-221007_LGG2_SEPAGFP-HZ455-2.lif - Series005-1

Pearson's Coefficient:
r=0.455

Overlap Coefficient:
r=0.693

r^2=k1xk2:
k1=0.906
k2=0.53

Using thresholds (thrA=67 and thrB=58)

Overlap Coefficient:
r=0.888

r^2=k1xk2:
k1=0.458
k2=1.718

Manders' Coefficients (original):
M1=1.0 (fraction of A overlapping B)
M2=1.0 (fraction of B overlapping A)

Manders' Coefficients (using threshold value of 67 for imgA and 58 for imgB):
M1=0.127 (fraction of A overlapping B)
M2=0.096 (fraction of B overlapping A)

Costes' automatic threshold set to 4 for imgA & 11 for imgB
Pearson's Coefficient:
r=0.239 (0.0 below thresholds)
M1=0.885 & M2=0.851
****
Image A: C1-220919_LGG2_SEPA1GFP-N2001.lif - Series001-1
Image B: C2-220919_LGG2_SEPA1GFP-N2001.lif - Series001-1

Pearson's Coefficient:
r=0.457

Overlap Coefficient:
r=0.637

r^2=k1xk2:
k1=1.366
k2=0.297

Using thresholds (thrA=45 and thrB=76)

Overlap Coefficient:
r=0.889

r^2=k1xk2:
k1=0.819
k2=0.965

Manders' Coefficients (original):
M1=1.0 (fraction of A overlapping B)
M2=1.0 (fraction of B overlapping A)

Manders' Coefficients (using threshold value of 45 for imgA and 76 for imgB):
M1=0.399 (fraction of A overlapping B)
M2=0.391 (fraction of B overlapping A)

Costes' automatic threshold set to 4 for imgA & 15 for imgB
Pearson's Coefficient:
r=0.393 (0.0 below thresholds)
M1=0.737 & M2=0.865

****
Image A: C1-220919_LGG2_SEPA1GFP-N2001.lif - Series002-1
Image B: C2-220919_LGG2_SEPA1GFP-N2001.lif - Series002-1

Pearson's Coefficient:
r=0.332

Overlap Coefficient:
r=0.623

r^2=k1xk2:
k1=3.044
k2=0.127

Using thresholds (thrA=56 and thrB=105)

Overlap Coefficient:
r=0.89

r^2=k1xk2:
k1=1.211
k2=0.655

Manders' Coefficients (original):
M1=1.0 (fraction of A overlapping B)
M2=1.0 (fraction of B overlapping A)

Manders' Coefficients (using threshold value of 56 for imgA and 105 for imgB):
M1=0.408 (fraction of A overlapping B)
M2=0.082 (fraction of B overlapping A)

Costes' automatic threshold set to 4 for imgA & 27 for imgB
Pearson's Coefficient:
r=0.218 (0.0 below thresholds)
M1=0.661 & M2=0.819

****
Image A: C1-220919_LGG2_SEPA1GFP-N2001.lif - Series003-1
Image B: C2-220919_LGG2_SEPA1GFP-N2001.lif - Series003-1

Pearson's Coefficient:
r=0.322

Overlap Coefficient:
r=0.628

r^2=k1xk2:
k1=1.821
k2=0.217

Using thresholds (thrA=36 and thrB=80)

Overlap Coefficient:

r=0.861

r^2=k1xk2:
k1=0.651
k2=1.138

Manders' Coefficients (original):
M1=1.0 (fraction of A overlapping B)
M2=1.0 (fraction of B overlapping A)

Manders' Coefficients (using threshold value of 36 for imgA and 80 for imgB):
M1=0.312 (fraction of A overlapping B)
M2=0.152 (fraction of B overlapping A)

Costes' automatic threshold set to 4 for imgA & 18 for imgB
Pearson's Coefficient:
r=0.237 (0.0 below thresholds)
M1=0.704 & M2=0.8
****
Image A: C1-220919_LGG2_SEPA1GFP-N2001.lif - Series004-1
Image B: C2-220919_LGG2_SEPA1GFP-N2001.lif - Series004-1

Pearson's Coefficient:
r=0.232

Overlap Coefficient:
r=0.573

r^2=k1xk2:
k1=3.579
k2=0.091

Using thresholds (thrA=54 and thrB=119)

Overlap Coefficient:
r=0.919

r^2=k1xk2:
k1=0.664
k2=1.273

Manders' Coefficients (original):
M1=1.0 (fraction of A overlapping B)
M2=1.0 (fraction of B overlapping A)

Manders' Coefficients (using threshold value of 54 for imgA and 119 for imgB):
M1=0.205 (fraction of A overlapping B)
M2=0.03 (fraction of B overlapping A)

Costes' automatic threshold set to 255 for imgA & 255 for imgB
Pearson's Coefficient:

r=0.0 (0.0 below thresholds)
M1=0.0 & M2=0.0
****
Image A: C1-220919_LGG2_SEPA1GFP-N2001.lif - Series005-1
Image B: C2-220919_LGG2_SEPA1GFP-N2001.lif - Series005-1

Pearson's Coefficient:
r=0.449

Overlap Coefficient:
r=0.661

r^2=k1xk2:
k1=1.08
k2=0.405

Using thresholds (thrA=65 and thrB=78)

Overlap Coefficient:
r=0.871

r^2=k1xk2:
k1=0.939
k2=0.807

Manders' Coefficients (original):
M1=1.0 (fraction of A overlapping B)
M2=1.0 (fraction of B overlapping A)

Manders' Coefficients (using threshold value of 65 for imgA and 78 for imgB):
M1=0.232 (fraction of A overlapping B)
M2=0.176 (fraction of B overlapping A)

Costes' automatic threshold set to 4 for imgA & 12 for imgB
Pearson's Coefficient:
r=0.309 (0.0 below thresholds)
M1=0.845 & M2=0.852

MUTANTS *lgg-1(ga)* CO-LOCALISATION SEPA-1::GFP et LGG-2

****
Image A: C1-220919_LGG2_SEPA1GFP-RD448001.lif - Series001-1
Image B: C2-220919_LGG2_SEPA1GFP-RD448001.lif - Series001-1

Pearson's Coefficient:
r=0.371

Overlap Coefficient:
r=0.531

r^2=k1xk2:

k1=0.725
k2=0.389

Using thresholds (thrA=53 and thrB=116)

Overlap Coefficient:
r=0.865

r^2=k1xk2:
k1=0.98
k2=0.764

Manders' Coefficients (original):
M1=0.999 (fraction of A overlapping B)
M2=1.0 (fraction of B overlapping A)

Manders' Coefficients (using threshold value of 53 for imgA and 116 for imgB):
M1=0.071 (fraction of A overlapping B)
M2=0.213 (fraction of B overlapping A)

Costes' automatic threshold set to 4 for imgA & 16 for imgB
Pearson's Coefficient:
r=0.281 (0.0 below thresholds)
M1=0.808 & M2=0.74
****
Image A: C1-220919_LGG2_SEPA1GFP-RD448001.lif - Series002-1
Image B: C2-220919_LGG2_SEPA1GFP-RD448001.lif - Series002-1

Pearson's Coefficient:
r=0.377

Overlap Coefficient:
r=0.516

r^2=k1xk2:
k1=1.521
k2=0.175

Using thresholds (thrA=58 and thrB=162)

Overlap Coefficient:
r=0.937

r^2=k1xk2:
k1=0.926
k2=0.948

Manders' Coefficients (original):
M1=0.999 (fraction of A overlapping B)
M2=1.0 (fraction of B overlapping A)

Manders' Coefficients (using threshold value of 58 for imgA and 162 for imgB):
M1=0.328 (fraction of A overlapping B)
M2=0.246 (fraction of B overlapping A)

Costes' automatic threshold set to 3 for imgA & 39 for imgB
Pearson's Coefficient:
r=0.313 (0.0 below thresholds)
M1=0.867 & M2=0.82
****
Image A: C1-220919_LGG2_SEPA1GFP-RD448001.lif - Series003-1
Image B: C2-220919_LGG2_SEPA1GFP-RD448001.lif - Series003-1

Pearson's Coefficient:
r=0.249

Overlap Coefficient:
r=0.493

r^2=k1xk2:
k1=1.293
k2=0.188

Using thresholds (thrA=53 and thrB=112)

Overlap Coefficient:
r=0.847

r^2=k1xk2:
k1=1.179
k2=0.609

Manders' Coefficients (original):
M1=0.999 (fraction of A overlapping B)
M2=1.0 (fraction of B overlapping A)

Manders' Coefficients (using threshold value of 53 for imgA and 112 for imgB):
M1=0.049 (fraction of A overlapping B)
M2=0.039 (fraction of B overlapping A)

Costes' automatic threshold set to 4 for imgA & 22 for imgB
Pearson's Coefficient:
r=0.096 (0.0 below thresholds)
M1=0.768 & M2=0.792
****
Image A: C1-221007_LGG2_SEPAGFP-RD448.lif - Series002-1
Image B: C2-221007_LGG2_SEPAGFP-RD448.lif - Series002-1

Pearson's Coefficient:
r=0.389

Overlap Coefficient:
r=0.516

r^2=k1xk2:
k1=0.834
k2=0.319

Using thresholds (thrA=65 and thrB=114)

Overlap Coefficient:
r=0.932

r^2=k1xk2:
k1=0.651
k2=1.334

Manders' Coefficients (original):
M1=0.999 (fraction of A overlapping B)
M2=1.0 (fraction of B overlapping A)

Manders' Coefficients (using threshold value of 65 for imgA and 114 for imgB):
M1=0.206 (fraction of A overlapping B)
M2=0.277 (fraction of B overlapping A)

Costes' automatic threshold set to 3 for imgA & 20 for imgB
Pearson's Coefficient:
r=0.316 (0.0 below thresholds)
M1=0.85 & M2=0.81
****
Image A: C1-221007_LGG2_SEPAGFP-RD448.lif - Series003-1
Image B: C2-221007_LGG2_SEPAGFP-RD448.lif - Series003-1

Pearson's Coefficient:
r=0.322

Overlap Coefficient:
r=0.472

r^2=k1xk2:
k1=0.91
k2=0.245

Using thresholds (thrA=49 and thrB=117)

Overlap Coefficient:
r=0.914

r^2=k1xk2:
k1=0.649
k2=1.286

Manders' Coefficients (original):
M1=1.0 (fraction of A overlapping B)
M2=1.0 (fraction of B overlapping A)

Manders' Coefficients (using threshold value of 49 for imgA and 117 for imgB):
M1=0.138 (fraction of A overlapping B)
M2=0.147 (fraction of B overlapping A)

Costes' automatic threshold set to 3 for imgA & 23 for imgB
Pearson's Coefficient:
r=0.214 (0.0 below thresholds)
M1=0.835 & M2=0.784
****
Image A: C1-221007_LGG2_SEPAGFP-RD448.lif - Series005-1
Image B: C2-221007_LGG2_SEPAGFP-RD448.lif - Series005-1

Pearson's Coefficient:
r=0.276

Overlap Coefficient:
r=0.447

r^2=k1xk2:
k1=1.003
k2=0.199

Using thresholds (thrA=67 and thrB=132)

Overlap Coefficient:
r=0.95

r^2=k1xk2:
k1=0.671
k2=1.344

Manders' Coefficients (original):
M1=0.999 (fraction of A overlapping B)
M2=1.0 (fraction of B overlapping A)

Manders' Coefficients (using threshold value of 67 for imgA and 132 for imgB):
M1=0.122 (fraction of A overlapping B)
M2=0.144 (fraction of B overlapping A)

Costes' automatic threshold set to 3 for imgA & 40 for imgB
Pearson's Coefficient:
r=0.182 (0.0 below thresholds)
M1=0.892 & M2=0.83
****
Image A: C1-221007_LGG2_SEPAGFP-RD448.lif - Series006
Image B: C2-221007_LGG2_SEPAGFP-RD448.lif - Series006

Pearson's Coefficient:
r=0.276

Overlap Coefficient:
r=0.418

r^2=k1xk2:
k1=1.01
k2=0.173

Using thresholds (thrA=46 and thrB=118)

Overlap Coefficient:
r=0.895

r^2=k1xk2:
k1=0.667
k2=1.201

Manders' Coefficients (original):
M1=0.999 (fraction of A overlapping B)
M2=1.0 (fraction of B overlapping A)

Manders' Coefficients (using threshold value of 46 for imgA and 118 for imgB):
M1=0.082 (fraction of A overlapping B)
M2=0.083 (fraction of B overlapping A)

Costes' automatic threshold set to 4 for imgA & 14 for imgB
Pearson's Coefficient:
r=0.185 (0.0 below thresholds)
M1=0.456 & M2=0.796
****
Image A: C1-221007_LGG2_SEPAGFP-RD448.lif - Series007-1
Image B: C2-221007_LGG2_SEPAGFP-RD448.lif - Series007-1

Pearson's Coefficient:
r=0.259

Overlap Coefficient:
r=0.458

r^2=k1xk2:
k1=0.665
k2=0.315

Using thresholds (thrA=49 and thrB=116)

Overlap Coefficient:
r=0.832

r^2=k1xk2:
k1=0.781
k2=0.886

Manders' Coefficients (original):
M1=0.999 (fraction of A overlapping B)
M2=1.0 (fraction of B overlapping A)

Manders' Coefficients (using threshold value of 49 for imgA and 116 for imgB):
M1=0.02 (fraction of A overlapping B)
M2=0.074 (fraction of B overlapping A)

Costes' automatic threshold set to 3 for imgA & 18 for imgB
Pearson's Coefficient:
r=0.184 (0.0 below thresholds)
M1=0.855 & M2=0.741
****
Image A: C1-221007_LGG2_SEPAGFP-RD448.lif - Series008-1
Image B: C2-221007_LGG2_SEPAGFP-RD448.lif - Series008-1

Pearson's Coefficient:
r=0.22

Overlap Coefficient:
r=0.414

r^2=k1xk2:
k1=1.119
k2=0.153

Using thresholds (thrA=55 and thrB=116)

Overlap Coefficient:
r=0.942

r^2=k1xk2:
k1=0.577
k2=1.538

Manders' Coefficients (original):
M1=0.999 (fraction of A overlapping B)
M2=1.0 (fraction of B overlapping A)

Manders' Coefficients (using threshold value of 55 for imgA and 116 for imgB):
M1=0.079 (fraction of A overlapping B)
M2=0.09 (fraction of B overlapping A)

Costes' automatic threshold set to 3 for imgA & 29 for imgB
Pearson's Coefficient:
r=0.15 (0.0 below thresholds)

M1=0.754 & M2=0.796
****
Image A: C1-221007_LGG2_SEPAGFP-RD448.lif - Series009-1
Image B: C2-221007_LGG2_SEPAGFP-RD448.lif - Series009-1

Pearson's Coefficient:
r=0.369

Overlap Coefficient:
r=0.524

r^2=k1xk2:
k1=0.763
k2=0.36

Using thresholds (thrA=65 and thrB=119)

Overlap Coefficient:
r=0.907

r^2=k1xk2:
k1=0.714
k2=1.151

Manders' Coefficients (original):
M1=1.0 (fraction of A overlapping B)
M2=1.0 (fraction of B overlapping A)

Manders' Coefficients (using threshold value of 65 for imgA and 119 for imgB):
M1=0.132 (fraction of A overlapping B)
M2=0.268 (fraction of B overlapping A)

Costes' automatic threshold set to 3 for imgA & 24 for imgB
Pearson's Coefficient:
r=0.293 (0.0 below thresholds)
M1=0.916 & M2=0.798
****
Image A: C1-221007_LGG2_SEPAGFP-RD448.lif - Series010-1
Image B: C2-221007_LGG2_SEPAGFP-RD448.lif - Series010-1

Pearson's Coefficient:
r=0.299

Overlap Coefficient:
r=0.541

r^2=k1xk2:
k1=1.585
k2=0.184

Using thresholds (thrA=49 and thrB=118)

Overlap Coefficient:
r=0.849

r^2=k1xk2:
k1=1.286
k2=0.56

Manders' Coefficients (original):
M1=0.999 (fraction of A overlapping B)
M2=1.0 (fraction of B overlapping A)

Manders' Coefficients (using threshold value of 49 for imgA and 118 for imgB):
M1=0.098 (fraction of A overlapping B)
M2=0.082 (fraction of B overlapping A)

Costes' automatic threshold set to 4 for imgA & 24 for imgB
Pearson's Coefficient:
r=0.162 (0.0 below thresholds)
M1=0.746 & M2=0.757
****
Image A: C1-221007_LGG2_SEPAGFP-RD448.lif - Series011-1
Image B: C2-221007_LGG2_SEPAGFP-RD448.lif - Series011-1

Pearson's Coefficient:
r=0.262

Overlap Coefficient:
r=0.446

r^2=k1xk2:
k1=0.699
k2=0.284

Using thresholds (thrA=53 and thrB=114)

Overlap Coefficient:
r=0.963

r^2=k1xk2:
k1=0.54
k2=1.717

Manders' Coefficients (original):
M1=1.0 (fraction of A overlapping B)
M2=1.0 (fraction of B overlapping A)

Manders' Coefficients (using threshold value of 53 for imgA and 114 for imgB):
M1=0.035 (fraction of A overlapping B)
M2=0.143 (fraction of B overlapping A)

Costes' automatic threshold set to 3 for imgA & 16 for imgB
Pearson's Coefficient:
r=0.249 (0.0 below thresholds)
M1=0.767 & M2=0.745
****
Image A: C1-221007_LGG2_SEPAGFP-RD448.lif - Series012-1
Image B: C2-221007_LGG2_SEPAGFP-RD448.lif - Series012-1

Pearson's Coefficient:
r=0.388

Overlap Coefficient:
r=0.509

r^2=k1xk2:
k1=0.671
k2=0.386

Using thresholds (thrA=60 and thrB=117)

Overlap Coefficient:
r=0.974

r^2=k1xk2:
k1=0.561
k2=1.694

Manders' Coefficients (original):
M1=1.0 (fraction of A overlapping B)
M2=1.0 (fraction of B overlapping A)

Manders' Coefficients (using threshold value of 60 for imgA and 117 for imgB):
M1=0.09 (fraction of A overlapping B)
M2=0.427 (fraction of B overlapping A)

Costes' automatic threshold set to 3 for imgA & 20 for imgB
Pearson's Coefficient:
r=0.365 (0.0 below thresholds)
M1=0.866 & M2=0.813
