# Supplementary material for: LGG-1/GABARAP lipidation is not required for autophagy and development in Caenorhabditis elegans
Source: eLife. 2023 Jul 3;12:e85748. doi: 10.7554/eLife.85748 (PMC10338037; doi:10.7554/eLife.85748)
Supplement: Figure 7—figure supplement 1—source data 1. [file elife-85748-fig7-figsupp1-data1.zip › Figure7-figure-supplement1-Source_Data1/A-J/Mean gray value signal diffus +points .pdf]

Number of families	1
Number of comparisons per family	28
Alpha	0,05

Dunn's multiple comparisons test	Mean rank diff,	Significant?	Summary	Adjusted P Value	
atg18GFP vs. GA atg18GFPL4440	-71,20	No	ns	>0,9999	A-B
atg18GFP vs. GAstop atg18GFPL4440	-34,57	No	ns	>0,9999	A-C
atg18GFP vs. lgg-1(tm3489) atg18GFP L4440	-254,1	Yes	****	<0,0001	A-D
atg18GFP vs. unc-51	169,0	Yes	***	0,0004	A-E
atg18GFP vs. GA atg18GFP RNAi unc-51	269,9	Yes	****	<0,0001	A-F
atg18GFP vs. GAstop atg18GFP unc-51	-132,5	Yes	*	0,0275	A-G
atg18GFP vs. lgg-1(tm3489) atg18GFP unc-51	157,1	Yes	**	0,0036	A-H
GA atg18GFPL4440 vs. GAstop atg18GFPL4440	36,63	No	ns	>0,9999	B-C
GA atg18GFPL4440 vs. lgg-1(tm3489) atg18GFP L4440	-182,9	Yes	****	<0,0001	B-D
GA atg18GFPL4440 vs. unc-51	240,2	Yes	****	<0,0001	B-E
GA atg18GFPL4440 vs. GA atg18GFP RNAi unc-51	341,1	Yes	****	<0,0001	B-F
GA atg18GFPL4440 vs. GAstop atg18GFP unc-51	-61,31	No	ns	>0,9999	B-G
GA atg18GFPL4440 vs. lgg-1(tm3489) atg18GFP unc-51	228,3	Yes	****	<0,0001	B-H
GAstop atg18GFPL4440 vs. lgg-1(tm3489) atg18GFP L4440	-219,5	Yes	****	<0,0001	C-D
GAstop atg18GFPL4440 vs. unc-51	203,6	Yes	****	<0,0001	C-E
GAstop atg18GFPL4440 vs. GA atg18GFP RNAi unc-51	304,5	Yes	****	<0,0001	C-F
GAstop atg18GFPL4440 vs. GAstop atg18GFP unc-51	-97,94	No	ns	0,2068	C-G
GAstop atg18GFPL4440 vs. lgg-1(tm3489) atg18GFP unc-51	191,7	Yes	****	<0,0001	C-H
lgg-1(tm3489) atg18GFP L4440 vs. unc-51	423,1	Yes	****	<0,0001	D-E
lgg-1(tm3489) atg18GFP L4440 vs. GA atg18GFP RNAi unc-51	524,1	Yes	****	<0,0001	D-F
lgg-1(tm3489) atg18GFP L4440 vs. GAstop atg18GFP unc-51	121,6	Yes	***	0,0004	D-G
lgg-1(tm3489) atg18GFP L4440 vs. lgg-1(tm3489) atg18GFP unc-51	411,2	Yes	****	<0,0001	D-H
unc-51 vs. GA atg18GFP RNAi unc-51	100,9	Yes	**	0,0050	E-F
unc-51 vs. GAstop atg18GFP unc-51	-301,5	Yes	****	<0,0001	E-G
unc-51 vs. lgg-1(tm3489) atg18GFP unc-51	-11,92	No	ns	>0,9999	E-H
GA atg18GFP RNAi unc-51 vs. GAstop atg18GFP unc-51	-402,4	Yes	****	<0,0001	F-G
GA atg18GFP RNAi unc-51 vs. lgg-1(tm3489) atg18GFP unc-51	-112,8	Yes	**	0,0040	F-H
GAstop atg18GFP unc-51 vs. lgg-1(tm3489) atg18GFP unc-51	289,6	Yes	****	<0,0001	G-H

Test details	Mean rank 1	Mean rank 2	Mean rank diff,	n1	n2
atg18GFP vs. GA atg18GFPL4440	411,6	482,8	-71,20	42	45
atg18GFP vs. GAstop atg18GFPL4440	411,6	446,2	-34,57	42	55
atg18GFP vs. lgg-1(tm3489) atg18GFP L4440	411,6	665,7	-254,1	42	140
atg18GFP vs. unc-51	411,6	242,6	169,0	42	145
atg18GFP vs. GA atg18GFP RNAi unc-51	411,6	141,7	269,9	42	130
atg18GFP vs. GAstop atg18GFP unc-51	411,6	544,1	-132,5	42	115
atg18GFP vs. lgg-1(tm3489) atg18GFP unc-51	411,6	254,5	157,1	42	100
GA atg18GFPL4440 vs. GAstop atg18GFPL4440	482,8	446,2	36,63	45	55
GA atg18GFPL4440 vs. lgg-1(tm3489) atg18GFP L4440	482,8	665,7	-182,9	45	140
GA atg18GFPL4440 vs. unc-51	482,8	242,6	240,2	45	145
GA atg18GFPL4440 vs. GA atg18GFP RNAi unc-51	482,8	141,7	341,1	45	130
GA atg18GFPL4440 vs. GAstop atg18GFP unc-51	482,8	544,1	-61,31	45	115
GA atg18GFPL4440 vs. lgg-1(tm3489) atg18GFP unc-51	482,8	254,5	228,3	45	100
GAstop atg18GFPL4440 vs. lgg-1(tm3489) atg18GFP L4440	446,2	665,7	-219,5	55	140
GAstop atg18GFPL4440 vs. unc-51	446,2	242,6	203,6	55	145
GAstop atg18GFPL4440 vs. GA atg18GFP RNAi unc-51	446,2	141,7	304,5	55	130
GAstop atg18GFPL4440 vs. GAstop atg18GFP unc-51	446,2	544,1	-97,94	55	115
GAstop atg18GFPL4440 vs. lgg-1(tm3489) atg18GFP unc-51	446,2	254,5	191,7	55	100
lgg-1(tm3489) atg18GFP L4440 vs. unc-51	665,7	242,6	423,1	140	145
lgg-1(tm3489) atg18GFP L4440 vs. GA atg18GFP RNAi unc-51	665,7	141,7	524,1	140	130
lgg-1(tm3489) atg18GFP L4440 vs. GAstop atg18GFP unc-51	665,7	544,1	121,6	140	115
lgg-1(tm3489) atg18GFP L4440 vs. lgg-1(tm3489) atg18GFP unc-51	665,7	254,5	411,2	140	100
unc-51 vs. GA atg18GFP RNAi unc-51	242,6	141,7	100,9	145	130
unc-51 vs. GAstop atg18GFP unc-51	242,6	544,1	-301,5	145	115
unc-51 vs. lgg-1(tm3489) atg18GFP unc-51	242,6	254,5	-11,92	145	100
GA atg18GFP RNAi unc-51 vs. GAstop atg18GFP unc-51	141,7	544,1	-402,4	130	115
GA atg18GFP RNAi unc-51 vs. lgg-1(tm3489) atg18GFP unc-51	141,7	254,5	-112,8	130	100
GAstop atg18GFP unc-51 vs. lgg-1(tm3489) atg18GFP unc-51	544,1	254,5	289,6	115	100
